# *Ac*NR1F orchestrates epidermal remodeling to enable host adaptation in *Angiostrongylus cantonensis*

**DOI:** 10.1371/journal.ppat.1014691

**Published:** 2026-10-12

**Authors:** Jinwei Lv, Yanling Ma, Jiaying Lai, Song Li, Ke Ning, Kangping Lu, Lihua Xiao, Yaoyu Feng, Dongjuan Yuan

**Affiliations:** State Key Laboratory for Animal Disease Control and Prevention, Center for Emerging and Zoonotic Diseases, College of Veterinary Medicine, South China Agricultural University, Guangzhou, China; Queen’s University Belfast, UNITED KINGDOM OF GREAT BRITAIN AND NORTHERN IRELAND

## Abstract

Nuclear receptor NR1F regulates circadian rhythms in mammals, metamorphosis in insects, and molting in free-living *Caenorhabditis elegans*; however, its role in parasitic nematode development remains elusive. *Angiostrongylus cantonensis,* a zoonotic pathogen that causes eosinophilic meningitis in humans, exhibits distinct host adaptation, with mice and most mammals serving as non-permissive hosts and rats as the permissive host. We combined multi-omic analyses, lentivirus-mediated RNAi, LC-MS/MS, and dual-luciferase assays to elucidate *Ac*NR1F functions in development and host adaptation. *Acnr1f* was expressed in the intestine, uterus, and seminal vesicle by *in situ* hybridization. *Acnr1f* showed high expression in L5 larvae and adults but low expression in L1-L4 larvae. *Acnr1f* expression was significantly higher in worms derived from rats than from mice; approximately 25% of co-expressed genes (918) were identified as potential downstream targets that may be integrated to regulate parasitic adaptation. RNAi reduced *Acnr1f* transcripts by 38.11% in female adults, resulting in significantly decreased recovery of L4 and L5 larvae and adults, a 64% reduction in egg production, and abnormal eggs with low hatching rate. Histological and ultrastructural analyses revealed thinned cuticle, reduced collagen fibers, and disrupted epidermal integrity. Transcriptomic analysis showed that differentially expressed genes were enriched in structural constituent of cuticle, metalloendopeptidase activity, extracellular matrix structural constituent. Dual-luciferase assays showed that the down-regulated collagen genes *col-12* and *col-144* were direct *Ac*NR1F targets (>300-fold and >40-fold induction, respectively). Differential levels of cholesterol, corticosterone, and 7α,25-dihydroxycholesterol between worms from rats and mice were identified as *Ac*NR1F ligands by LC-MS/MS and ligand-binding assays. Thus, *Ac*NR1F is a ligand-activated nuclear receptor that directly regulates target genes to influence development and reproduction of *A. cantonensis*. The distinct availability of host-derived ligands and differential *Ac*NR1F expressive level between worms from rodents highlights this receptor as a critical molecular switch for parasitic adaptation, providing mechanistic insights into host-pathogen interactions.

## Author summary

NR1F, a member of the nuclear receptor superfamily, regulates circadian rhythms in mammals, directs metamorphosis in insects, and controls molting in the free-living nematode *Caenorhabditis elegans*. However, its functional significance in parasitic nematodes has yet to be determined. *Angiostrongylus cantonensis*, the rat lungworm, is a zoonotic pathogen that causes eosinophilic meningitis in humans and exhibits striking host-dependent developmental differences between permissive (rat) and non-permissive (mouse) hosts. Elucidating the molecular mechanisms underlying this host adaptation is critical for developing novel intervention strategies. In this study, we characterized *Ac*NR1F, which possesses canonical DNA-binding and ligand-binding domains. Using lentivirus-mediated RNA interference combined with transcriptomic analyses, we demonstrated that *Ac*NR1F regulated development and reproduction, maintained epidermal integrity, and associated with host adaptation in *A. cantonensis*. We further showed that cholesterol, corticosterone, and 7α,25-dihydroxycholesterol from rodent hosts served as ligands for *Ac*NR1F, and identified the collagen genes *col-12* and *col-144* as direct downstream targets. These findings illuminate how helminth parasites sense and respond to host environments through *Ac*NR1F. This work identifies a druggable nuclear receptor pathway that may be exploited to disrupt parasite survival across different host species.

## Introduction

*Angiostrongylus cantonensis* is a parasitic nematode that infects a broad range of mammalian hosts, including humans, rhesus monkeys, guinea pigs, horses, dogs, rabbits, rats, and mice. Infection with *A. cantonensis* causes eosinophilic meningitis or eosinophilic meningoencephalitis in humans and most animals [1–3]. The rat serves as the permissive host for *A. cantonensis*. L5 larvae migrate to the pulmonary arteries, develop into adult worms, and lay eggs. In contrast, humans, mice, and other mammals are non-permissive hosts. In these hosts, worms exhibit developmental delay and stunted growth, cuticular damage, and failure to reach the lungs, remaining trapped in the brain and causing severe pathological damage [4,5]. These observations suggest that stage-specific developmental programs guide the parasite through the host central nervous system (CNS) and ultimately determine whether the parasite matures in the pulmonary arteries (permissive host) or becomes sequestered in the brain (non-permissive host). However, the molecular mechanisms governing this host-dependent developmental plasticity remain poorly understood.

Nuclear hormone receptors (NHRs) constitute an evolutionarily conserved superfamily of ligand-activated transcription factors that govern metabolism, development, circadian rhythms, inflammation, and immunity [6]. NR1F, a member of the NR1 subfamily, is widely distributed across mammals, insects, and nematodes. In humans, NR1F comprises three retinoic acid receptor–related orphan receptors (RORα, RORβ, and RORγ), each displaying tissue-restricted expression patterns [7]. These receptors bind RORE DNA elements within target-gene promoters to regulate circadian rhythms, lipid metabolism, and immune responses [8,9]. In *Drosophila melanogaster*, the single NR1F ortholog hormone receptor 3 (HR3) integrates 20-hydroxyecdysone (20E) signaling to coordinate embryogenesis and larval stage transitions [10].

In the free-living *Caenorhabditis elegans*, the NR1F homolog NHR-23 is predominantly expressed in hypodermal cells. Its transcript levels fluctuate in a reiterated pattern across larval molts [11], and RNAi-mediated knockdown disrupts ecdysis, causing phenotypes characterized by down-regulation of key molting genes (*mlt-8–11*) and cuticular collagens (*dpy-2, dpy-3, dpy-5, dpy-7, dpy-8, dpy-10*) [12]. NHR-23 further controls seam-cell and hypodermal development, maintaining cuticular morphology and function [13]. The receptor is modulated by microRNAs (*let-7* and *lin-4*) and interacts with the rhythmic ortholog *LIN-42* to regulate developmental rhythms reminiscent of circadian oscillations [14–16].

Given the critical role of NR1F in regulating molting and development in free-living nematodes, we hypothesized that *Ac*NR1F may function as a key regulator of stage-specific development and host adaptation in *A. cantonensis*. To test this hypothesis, we analyzed transcriptomic data from worms recovered from permissive (rat) and non-permissive (mouse) hosts, characterized *Ac*NR1F expression dynamics across developmental stages, and identified downstream regulatory targets and cognate ligands. This study establishes a mechanistic foundation for understanding NR1F-mediated regulation of parasitic development and host adaptation.

## Results

### *Acnr1f* is a nuclear receptor highly expressed in L5 larvae and female adults

Phylogenetic analysis showed that NR1F receptors are evolutionarily conserved across nematodes (Fig 1A). We cloned and expressed the *A. cantonensis nr1f* gene (GenBank accession: PX661152), which encodes a 451-amino-acid protein (50 kDa) sharing ~50% identity with *C. elegans* NHR-23 (Fig 1B and Fig A in S1 Appendix). Expression profiling showed that *Acnr1f* transcript levels were low during L1-L4 larval stages but markedly increased in L5 larvae and adult worms, with female adults exhibiting higher expression than male adults (Fig 1C and Table A in S1 Appendix). This expression profile was derived from RNA-seq data deposited in NCBI SRA under accession SRP127889 (BioProject PRJNA350405). Chromogenic *in situ* hybridization (CISH) further revealed that *Acnr1f* mRNA was predominantly localized to the cytoplasm of intestinal, uterine, muscle, and seminal vesicle cells (Fig 1D). CISH result revealed substantially higher *Acnr1f* mRNA levels in eggs within the uterus compared with the seminal vesicle. Cell-PLoc prediction identified typical nuclear localization signals in *Ac*NR1F. Conserved domain analysis using CD-search and SMART revealed a canonical ZnF_C4 DNA-binding domain (DBD) and a HOLI ligand-binding domain (LBD) (Fig 1E). NR1F orthologs from *A. cantonensis*, *C. elegans*, and *D. melanogaster* exhibited high sequence similarity in the DBD but low similarity in the LBD (Fig 1E).

**Fig 1:**
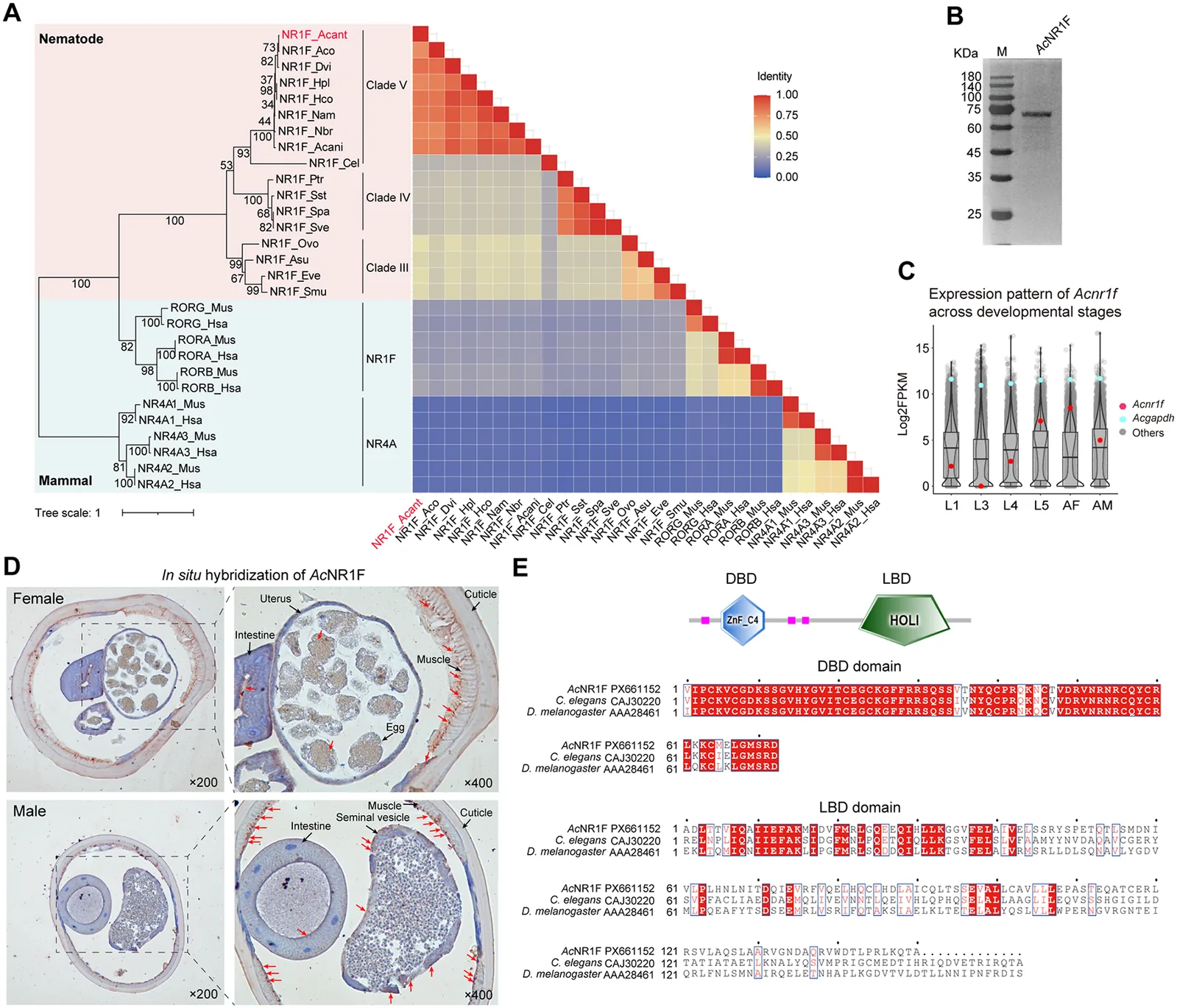
Identification and analysis of expression pattern of *Acnr1f.* **(A)** Phylogenetic analysis and sequence identity of NR1F from human, mouse and nematodes. Bootstrap values are shown in the nodes. The scale bar represents the number of amino acid substitutions per site. **(B)** SDS-PAGE of purified recombinant *Ac*NR1F with his-tag by prokaryotic expression. **(C)** Expression pattern of *Acnr1f* across developmental stages based on RNA-seq. **(D)** Analysis of *Acnr1f* gene expression by chromogenic *in situ* hybridization. Red arrow showed the expression of *Acnr1f* mRNA in the cytoplasm. **(E)** Schematic of the DBD and LBD in *Ac*NR1F and alignment analysis of LBD and DBD domains of *A. cantonensis*, *C. elegans*, and *D. melanogaster*. The raw data underlying this Figure are available in S1 Appendix and S1 Rawgel.

### *Acnr1f* potentially regulates downstream gene expression

Hierarchical clustering of transcriptomic data from worms recovered from rats and mice showed that mouse-derived larvae at 28 days post-infection (dpi) clustered with rat-derived larvae at 21 dpi, indicating developmental delay of *A. cantonensis* in the non-permissive host (Fig 2A). Differential expression analysis identified 314, 511, and 1,800 differentially expressed genes (DEGs) at early L4 (14 dpi), L4 (21 dpi), and L5 (28 dpi) stages, respectively (Fig 2B). Comparative transcriptomics showed that DEGs between mouse- and rat-derived worms at 28 dpi were enriched in functional categories including phosphorylation, proteolysis, innate immune response, collagen and cuticulin-based cuticle, transmembrane transport, determination of adult lifespan, spermatogenesis, embryo development ending in birth or egg hatching, lipid metabolic process, and oviposition (Fig 2C and Table B in S1 Appendix). Compared with rat-derived worms, up-regulated DEGs in mouse-derived worms were enriched in Gene Ontology (GO) terms associated with proteolysis, molting cycle, collagen and cuticulin-based cuticle, neuropeptide signaling pathway, and determination of adult lifespan (Fig B in S1 Appendix). Conversely, down-regulated DEGs were enriched in GO terms related to phosphorylation, protein dephosphorylation, regulation of cell population proliferation, spermatogenesis, transmembrane transport, and collagen trimer (Fig B in S1 Appendix). Scanning electron microscopy (SEM) analysis showed that mouse-derived larvae exhibited epidermal damage with deformed cuticular annuli compared to rat-derived counterparts (Fig 2D). *Acnr1f* expression was significantly higher in rat-derived larvae compared to mouse-derived larvae, suggesting its involvement in host-specific developmental regulation (Fig 2E and Table C in S1 Appendix). Weighted gene co-expression network analysis (WGCNA) identified a turquoise module comprising 3,116 genes showing stage-dependent up-regulation across development stages (Fig 2F).

**Fig 2:**
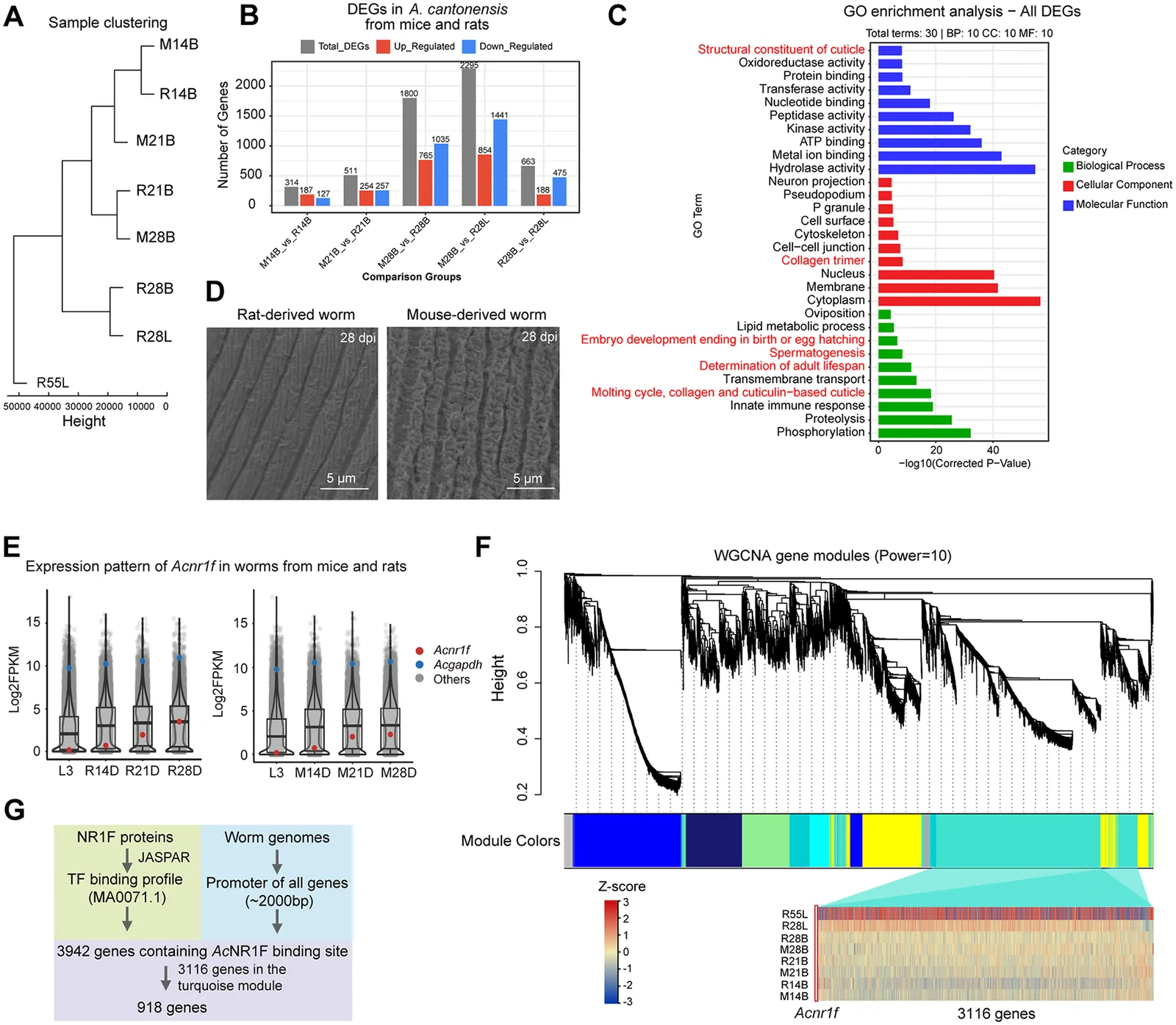
*Acnr1f* may be associated with host adaptation of *A. cantonensis* from mice and rats. **(A)** Sample clustering of the transcriptome data of *A. cantonensis* from mice (14, 21 and 28 dpi) and rats (14, 21, 28 and 55 dpi). Height represents dissimilarity between samples. M14B, M21B and M28B: worms from mouse brain at 14, 21 and 28 dpi; R14B: worms from rat brain at 14, 21 and 28 dpi; R28L and R55L: worms from rat lung at 28 and 55 dpi. **(B)** The number of differentially expressed genes in *A. cantonensis* from mice and rats. **(C)** Functional annotations of the main DEGs in *A. cantonensis* from mice and rats at 28 dpi. **(D)** SEM observation of epidermis of *A. cantonensis* from mice and rats*.* **(E)** Expression pattern of *Acnr1f* in *A. cantonensis* from mice and rats. **(F)** Cluster dendrogram of co-expression module clustering constructed by WGCNA method on the transcriptome data of *A. cantonensis*. Gene expression pattern of 3116 co-expressed genes in turquoise module. **(G)** The potential *Ac*NR1F downstream genes by genome-wide analysis were mainly found in co-expressed genes in turquoise module by WGCNA analysis. The raw data underlying this Figure are available in S1 Appendix and S1 Rawgel.

To identify transcriptional targets, we employed the JASPAR database to predict RORα binding motifs (MA0071.1) and screened ~2 kb promoter regions upstream of all genes in the *A. cantonensis* genome (CNSA accession: CNP0000467). MEME analysis identified 3,942 genes containing ≥1 MA0071.1 motif, of which 918 overlapped with the turquoise WGCNA module (Fig 2G and Table D in S1 Appendix). This indicates that approximately 25% of co-expressed genes are potential downstream targets of *Ac*NR1F.

### Lentivirus-mediated RNAi of *Acnr1f* impairs worm development and female reproduction

Three dsRNA interference fragments (nr1f-94, nr1f-894, and nr1f-12519) were synthesized (Table E in S1 Appendix) and administered separately to L5 larvae. Quantitative PCR (qPCR) analysis demonstrated that nr1f-894 and nr1f-1219 siRNAs effectively suppressed *Acnr1f* transcript expression after 24 h (Fig 3A and Table F in S1 Appendix). To achieve sustained RNAi in the parasitic environment, these fragments were cloned into a lentiviral vector system. Recombinant lentiviruses (LVs) carrying shRNAmir-nr1f-894 and shRNAmir-nr1f-1219, as well as empty vector control, were packaged in 293T cells; these vectors co-expressed TurboGFP from the same CMV-driven transcript as the shRNAmir, so green fluorescence marks transduced cells (Fig 3B). The shRNAmir, inserted between mir30a’ and mir30b’ in the pGIPZ vector, was transcribed as a ~ 300–400 bp mir30a’-shRNAmir-mir30b’ fragment (Fig 3C). RT-PCR detection of mir30a’-shRNAmir-mir30b’ from total RNA identified the fragment in LV-Acnr1f-894 and LV-Acnr1f-1219 groups, with no amplification in the wild-type (WT) group and a smaller fragment (~200–300 bp) in the empty vector (EV) control (Fig 3D). Notably, *Acnr1f* expression was significantly down-regulated in female adult worms from the *Acnr1f*-894 group but not the *Acnr1f*-1219 group (Fig 3E and Table G in S1 Appendix); therefore, subsequent functional analyses focused on the *Acnr1f*-894 group.

**Fig 3:**
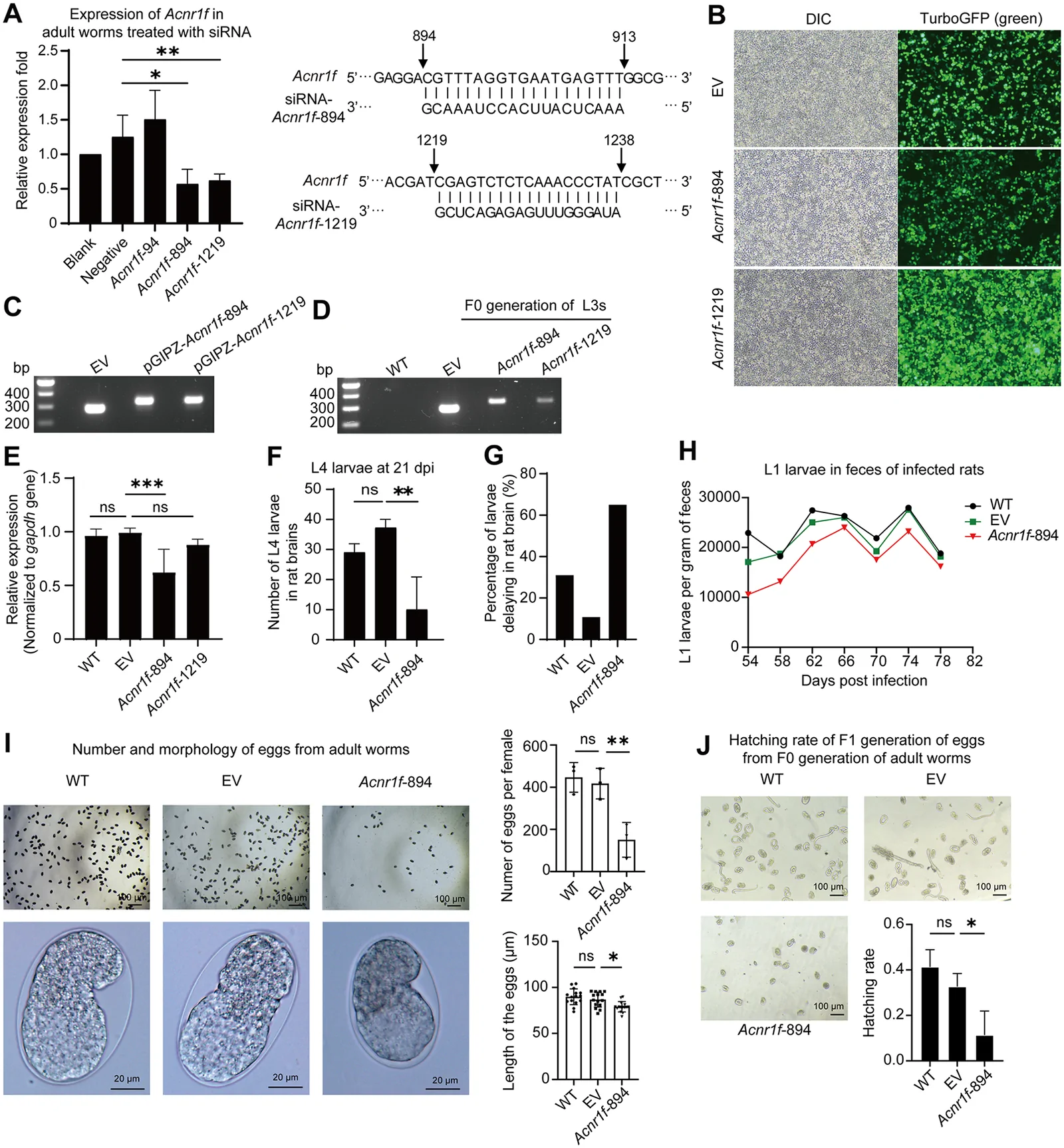
Lentivirus-mediated RNAi of *Acnr1f* inhibits the development and reproduction of *A. cantonensis.* **(A)** Identification of RNAi effects of three siRNAs on adult worms by qPCR analysis and alignment of the nucleotide sequences of siRNA-*Acnr1f*-894 and siRNA-*Acnr1f*-1219 with *Acnr1f* gene. **(B)** Packaging of recombinant lentiviruses in 293T cells co-transfected with the pGIPZ shRNAmir vector (nr1f-894, nr1f-1219, or empty-vector control) and packaging plasmids. Green fluorescence is TurboGFP, co-expressed with the shRNAmir from a single CMV-driven transcript, marking transfected cells. **(C)** PCR identification of shRNAmir-*Acnr1f*-894 and shRNAmir-*Acnr1f*-1219 in the lentivirus from the supernatant of 293T cells. **(D)** PCR analysis of total DNA to identify shRNAmir-*Acnr1f*-894 and shRNAmir-*Acnr1f*-1219 integrated into F0 generation of L3s. **(E)** *Acnr1f* gene transcription in F0 generation of adult worms treated with lentivirus LV-*Acnr1f*-894 and LV-*Acnr1f*-1219 at MOI of 400 by qPCR analysis (n = 3). **(F)** Number of L4 larvae (21 dpi) from rat brains (n = 4). **(G)** Percentage of L5 larvae (28 dpi) delaying in rat brain treatment with LV-*Acnr1f*-894 (n = 4). **(H)** Number of L1 larvae from rat feces (n = 3). **(I)** Number and morphology of F1 generation of eggs from F0 generation of adult worms. **(J)** Hatching rate of F1 generation of eggs to L1 larvae from F0 generation of adult worms. WT: wild type; NC: non-specific control siRNA; siRNA-*Acnr1f*-894 and siRNA-*Acnr1f*-1219: the positions of the siRNAs on the *Acnr1f* gene. Data were mean ± SD of three independent experiments (n = 3). *p < 0.05, **p < 0.005. The raw data underlying this Figure are available in S1 Appendix and S1 Rawgel.

RNAi-mediated *Acnr1f* knockdown resulted in significant developmental defects: (i) reduced L4 larval burdens in infected rat brains (Fig 3F and Table H in S1 Appendix); (ii) higher percentages of residual larvae in brains with impaired migration to the lungs at 28 dpi (Fig 3G and Table I in S1 Appendix); (iii) decreased L1 larval output in feces (Fig 3H and Table J in S1 Appendix); (iv) reduced egg production by adult worms isolated from pulmonary blood vessels, accompanied by morphological abnormalities including spherical deformation (Fig 3I and Tables K-L in S1 Appendix); and (v) compromised egg hatchability (Fig 3J and Table M in S1 Appendix). These results demonstrate that *Acnr1f* is essential for larval development and female reproduction.

### *Acnr1f* RNAi disrupts cuticle formation and *collagen* expression

SEM revealed aberrant cuticular morphology in larvae (28 dpi) and adult worms (50 dpi) from the *Acnr1f*-894 group, characterized by irregular annuli and furrows and rough surface texture (Fig 4A). Transmission electron microscopy (TEM) showed ultrastructural defects in both larvae and adults (Fig 4A). Masson staining further validated cuticular thinning in the *Acnr1f*-894 group compared to controls (Fig 4B and Table N in S1 Appendix). Principal component analysis (PCA) of transcriptomic data of the adult worms from rat lung showed clear separation among WT, EV and *Acnr1f*-894 groups (Fig 4C). A total of 603 DEGs were identified exclusively in the *Acnr1f*-894 group compared to the EV group (Fig 4D), comprising 258 up-regulated and 345 down-regulated genes. Many more genes showed down-regulated expression in the *Acnr1f*-894 group compared to the EV group (Fig 4E). These DEGs were enriched in GO terms related to cuticulin-based cuticle molting cycle, post-embryonic body morphogenesis, cell differentiation, cell adhesion, and intracellular signal transduction (Fig 4F and Table O in S1 Appendix). Pfam domain searches identified 92 collagen genes in the *A. cantonensis* genome, 36 were expressed at Log2(FPKM) > 1, and 12 of these were down-regulated in the *Acnr1f*-894 group compared with the control (Fig 4G and Table P in S1 Appendix). Among the top ten highly expressed genes, five collagen genes (Angca_01175, Angca_03162, Angca_12810, Angca_01778, and Angca_10659) were identified as predicted transcriptional targets. Two collagen genes, Angca_01175 (*Accol-12*) and Angca_01778 (*Accol-144*), were selected for validation by dual-luciferase reporter assays. *Ac*NR1F bound to the promoters of both genes, activating *Accol-12* (>300-fold) and *Accol-144* (>40-fold) (Fig 4H and Figs C-D in S1 Appendix and Table Q in S1 Appendix). The expression patterns of *Accol-12* and *Accol-144* across developmental stages were similar to that of *Acnr1f* (Fig 1C and 4I and Table R in S1 Appendix).

**Fig 4:**
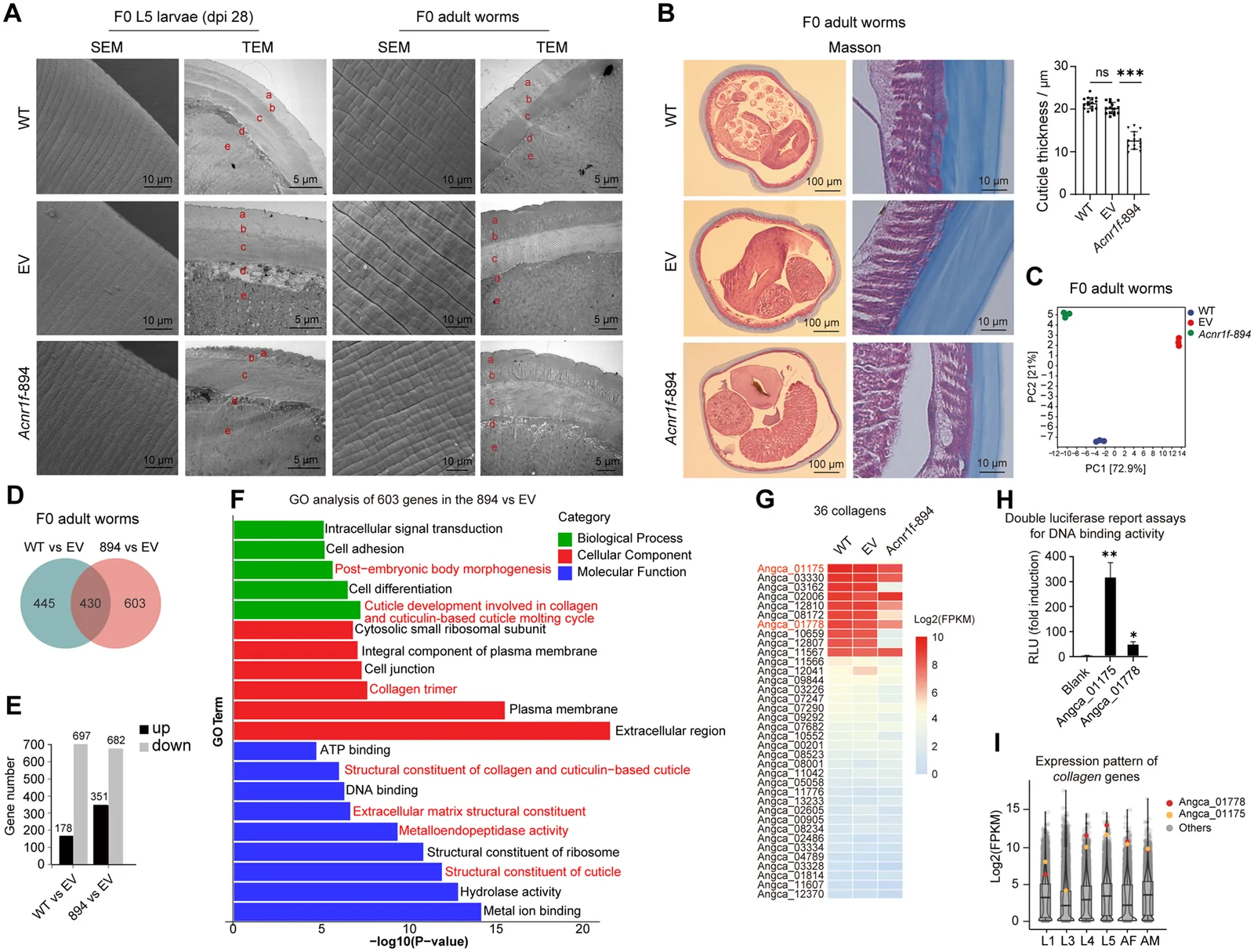
Interference of *Acnr1f* affected cuticle formation by regulating *collagen* expression. **(A)** SEM and TEM observations of F0 generation of L5 larvae (28 dpi) and adult worms from infected rats. a: Epicuticle, b: Medial region, c: Hypodermis, d: Basal lamina, e: Muscle. **(B)** Masson staining of F0 generation of adult worms for observing cuticle thickness. **(C)** PCA analysis of transcriptomic data of F0 generation of adult worms. **(D)** Venn diagram analysis of DEGs of F0 generation of adult worms among WT, EV and RNAi groups. **(E)** Gene numbers of up-regulated and down-regulated DEGs of F0 generation of adult worms among WT, EV and RNAi groups. **(F)** GO enrichment of DEGs of F0 generation of adult worms between EV and RNAi groups. **(G)** Heatmap analysis of expression pattern of 36 collagens in the F0 generation of adult worms with Log2(FPKM)>1 among WT, EV and RNAi groups. **(H)** Identification of two *collagen* genes as downstream targets of *Ac*NR1F by dual-luciferase reporter assays (n = 3). **(I)** Expression profiles of these two *collagen* genes across developmental stages of *A. cantonensis*. Significance versus pGL3-Basic control: *p < 0.05, **p < 0.01, ***p < 0.001, ****p < 0.0001. The raw data underlying this Figure are available in S1 Appendix and S1 Rawgel.

### Distinct availability of rodent-derived steroids serves as ligands for *Ac*NR1F

Liquid chromatography-tandem mass spectrometry (LC-MS/MS)-based steroidomics identified 43 distinct steroids across non-infected and infected rat/mouse brains and larvae at 28 dpi. Twenty-two steroids were quantifiable, with six showing higher abundance in larvae than in infected rat brains at 28 dpi (n = 6): 7α,25-dihydroxycholesterol, 25-hydroxycholesterol, corticosterone, 11-dehydrocorticosterone, β-sitosterol, and 7α,27-dihydroxycholesterol (Fig 5A and Table S in S1 Appendix). Lathosterol, cholesterol, corticosterone, and 7α,25-dihydroxycholesterol were the predominant steroids in larvae, with significant quantitative differences between rat- and mouse-derived larvae (Fig 5B). Conversely, 24-hydroxycholesterol was significantly lower in larvae than in host brains (Table S in S1 Appendix). Seven steroids (desmosterol, pregnenolone, cortisol, 16α-hydroxyestrone, 5α-pregnane-3,20-dione, 5β-androsterone, and 11-hydroxyandrosterone) were undetectable in larvae but accumulated in infected rat brains (Fig 5A and Table S in S1 Appendix). These results indicate partial sharing yet distinct maintenance of host- and parasite-specific steroidomes.

**Fig 5:**
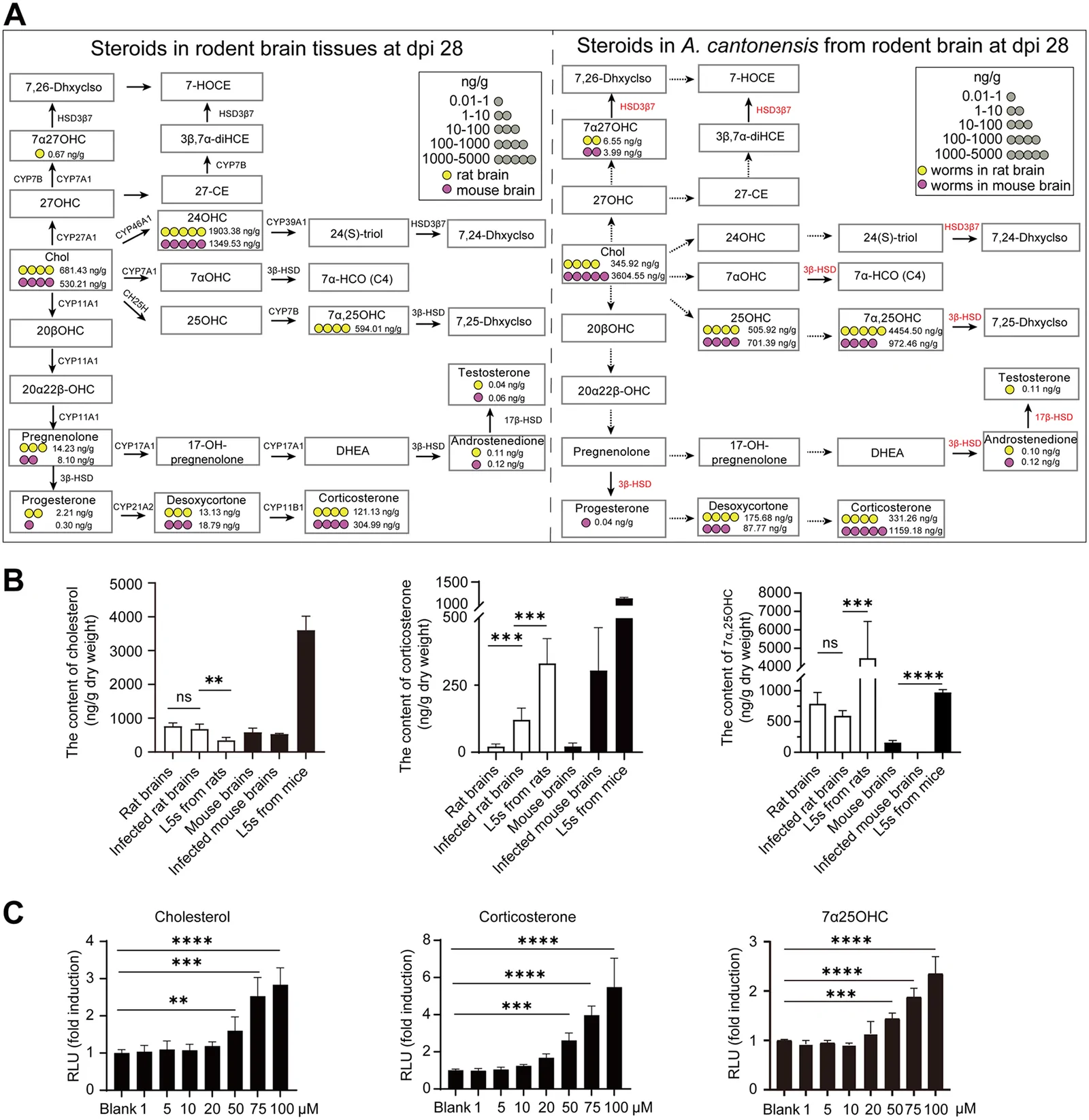
The ligand binding domain of *Ac*NR1F could be induced by steroids assessed by dual-luciferase reporter assays. **(A)** Steroid biosynthesis pathway in worms from rodent brains and in infected rodent brains. Steroid levels in the brain of SD rats, BALB/c mice, and larvae at 28 dpi (n = 6), as determined by LC-MS/MS. **(B)** Cholesterol, corticosterone, and 7α25-OHC contents in worms and rodent brains at 28 dpi by LC-MS/MS. **(C)** Binding activities of cholesterol, corticosterone and 7α25-OHC to *Ac*NR1F assessed by dual-luciferase reporter assays. The blank control group was treated identically to the other experimental groups, except that no ligand was added. Chol, cholesterol; 27OHC, 27-hydroxycholesterol; 7α27-OHC, 7α,27-Dihydroxycholesterol; 7,26-Dhxyclso, 7α,26-Dihydroxy-4-cholesten-3-one; 7-HOCE, 7α-Hydroxy-3-oxo-4-cholestenoate; 3β,7α-diHCE, 3β,7α-Dihydroxy-5-cholestenoate; 27-CE, 3β-Hydroxy-5-cholestenoate; 24OHC, 24-Hydroxycholesterol; 24(S)-triol, (24S)-Cholest-5-ene-3β,7α,24-triol; 7,24-Dhxyclso, 7α,24-Dihydroxy-4-cholesten-3-one; 7α-OHC, 7α-Hydroxycholesterol; 7α-HCO, 7α-Hydroxy-4-cholesten-3-one; 25OHC, 25-Hydroxycholesterol; 7α25-OHC, 7α,25-Dihydroxycholesterol; 7,25-Dhxyclso, 7 α,25-Dihydroxy-4-cholesten-3-one; 20β-OHC, 20β-Hydroxycholesterol; 20α22β-OHC, 20α,22β-Dihydroxycholesterol; 17-OH-pregnenolone, 17α-Hydroxypregnenolone; DHEA, Dehydroepiandrosterone; Deoxycortone, 11-Deoxycorticosterone. **p < 0.01, ***p < 0.001, ****p < 0.0001. The raw data underlying this Figure are available in S1 Appendix and S1 Rawgel.

Comparative analysis revealed that cholesterol, lathosterol, and corticosterone were ~10-, ~ 10-, and ~4-fold higher, respectively, in mouse-derived larvae compared to rat-derived larvae, whereas 7α,25-dihydroxycholesterol was ~ 4.5-fold lower (Fig 5A-5B and Table S in S1 Appendix). Lathosterol is an intermediate in cholesterol biosynthesis, and cholesterol serves as the substrate for steroid biosynthesis—supported by the presence of HSD3β7, 3β-HSD, and 17β-HSD in larvae. Thus, the levels of lathosterol and cholesterol in larvae from mice and rats were inconsistent with their respective levels in host brains, however, the distinct levels of 7α,25-dihydroxycholesterol and corticosterone in larvae from mice and rats were consistent with their respective levels in host brains. Thus, the interactions of cholesterol, corticosterone, and 7α,25-dihydroxycholesterol with *Ac*NR1F was conducted using dual-luciferase reporter assays. Dose-response experiments (1–100 μM) showed that all three steroids bound *Ac*NR1F-LBD and activated transcription in a dose-dependent manner, with responses first detectable at 50 μM and increasing thereafter (Fig 5C and Fig E in S1 Appendix and Table T in S1 Appendix). These results show that *Ac*NR1F is a ligand-activated nuclear receptor that utilizes host-derived cholesterol, corticosterone, and 7α,25-dihydroxycholesterol to exert its functions.

## Discussion

### Developmental arrest of *A. cantonensis* in non-permissive hosts

Rats serve as permissive definitive hosts where L5 larvae migrate to the lungs and develop into reproductive adults. By contrast, larvae in mice become arrested in the brain, exhibiting stunted growth, epidermal damage, and failure to reach the lungs. Transcriptomic analysis revealed that worms from mice exhibited delayed developmental progression compared to those from rats at the same time, ultimately arresting at a developmental stage at 28 dpi comparable to 21 dpi in rats. Extensive DEGs were observed in both rodent-derived larvae at 28 dpi with enrichment in reproduction, cuticle collagen, and lipid metabolism. These findings establish a molecular foundation for understanding host-dependent developmental plasticity in parasitic nematodes.

### RNAi-mediated functional analysis of *Acnr1f*

We successfully applied LV-mediated RNAi to silence *Acnr1f* expression in F0 generation female adults of *A. cantonensis*, achieving a 38% reduction in transcript levels compared to control group. However, limited recovery of worms from the interference group prevented establishment of a stable line, restricting our analysis to the F1 generation. In *S. ratti*, *piggyBac*-mediated chromosomal integration improves heritability but still yields F1 GFP expression at only 3.5–8.9% and F2 expression at 0.4–2.5%, requiring multiple host passages and manual screening of thousands of larvae to establish stable lines [17]. Consequently, high-throughput selection using fluorescent reporters is not currently feasible in this system. With respect to drug-selectable markers, we have considered introducing antibiotic resistance cassettes (e.g., NeoR or PuroR). While these markers have proven effective in *C. elegans* [18] and transgenesis has been achieved in the related parasitic nematode *Parastrongyloides trichosuri* [19], species-specific promoter optimisation and drug concentration protocols remain to be established for our target parasite. Previous studies in *Nippostrongylus brasiliensis* demonstrated that proviral DNA can be transmitted to F1 offspring [20] and persist through the F4 generation, with reduced lipid content observable even in F6 worms [21]. Nevertheless, this approach provides a technical foundation for future functional studies of parasitic nematode genes.

### *Ac*NR1F regulates development and reproduction

*Acnr1f* exhibits minimal expression during L1-L3 larval stages, with up-regulation in parasitic stages and peak expression in female adults from rats, suggesting functional association with parasitism. Furthermore, *Acnr1f* knockdown caused the reduced worm recovery that likely reflects developmental and reproductive impairment, coupled with compromised cuticle integrity, resulting in decreased survival to adulthood. GO enrichment analysis of DEGs identified significant terms including “post-embryonic body morphogenesis” following *Acnr1f* knockdown. In *C. elegans*, pulsatile expression of NHR-23 during germ cell development corresponds to its functions in reproductive development [22]. NHR-23 is continuously expressed in primary spermatocytes; its deletion causes spermatocyte arrest at metaphase I, preventing chromosome segregation, cytokinesis, and assembly of fibrous bodies composed of major sperm protein, ultimately leading to immotile sperm [23]. Thus, these functions on regulating reproduction may be conserved across nematode species.

*Acnr1f* exhibits lower expression in mouse-derived larvae than in rat-derived larvae. There is delayed development of larvae in mice compared in rats, that likely explains the lower *Acnr1f* expression. Analysis of transcriptomic data identified 3116 co-expressed genes of *Acnr1f* in worms from mice and rats. Among these, 918 potential *Ac*NR1F downstream target genes showed substantial overlap with co-expressed genes. Furthermore, the GO enrichment of DEGs in larvae from mice and rats was similar to GO enrichment of DEGs in the adult females following *Acnr1f* knockdown. These results suggest that *Ac*NR1F coordinates a regulatory network involving downstream targets that might regulate nematode development, epidermal integrity, and reproduction. The phenotypic consequences of *Acnr1f* interference may reflect a failure of parasitic adaptation.

In *C. elegans*, NR1F has been shown to be modulated by microRNAs (*let-7* and *lin-4*) and to interact with the rhythmic ortholog LIN-42, thereby regulating developmental rhythms that resemble circadian oscillations [14–16]. Using miRanda v3.3a, IntaRNA, and Sfold, we predicted a high-confidence *lin-4-5p* binding site within the 3′ UTR of *Acnr1f* at nucleotides 569–593, as well as a putative *let-7-5p* binding site at nucleotides 327–344. Therefore, we speculate that *Acnr1f* may likewise be subject to microRNA-mediated regulation, thereby modulating nematode development.

### *Ac*NR1F directly activates cuticle collagen genes to drive epidermal remodeling

*Acnr1f* knockdown caused misaligned cuticular annulations, marked disruption of epidermal integrity and disorganized cuticle ultrastructure in *A. cantonensis*. GO enrichment of DEGs in adult females in *Acnr1f*-894 group compared to EV group were enriched in “structural constituent of collagen and cuticulin-based cuticle”, “extracellular matrix structural constituent”, and “metalloendopeptidase activity”. Masson staining and electron microscopy observations showing reduced dermal collagen fibers. Transcriptomic data indicated down-regulation of collagen genes in the interference group, and *col-12* and *col-144* were identified as direct downstream targets of *Ac*NR1F. Thus, *Ac*NR1F is a critical regulator of cuticle formation. Importantly, these pathways overlap substantially with those identified in the *C. elegans nhr-23* RNAi microarray study, where down-regulated genes were strongly enriched for collagens, cuticlins, and aECM components involved in molting and cuticle development [12]. This cross-species comparison supports a conserved regulatory role for NR1F/NHR-23 in coordinating cuticle collagen gene expression.

Epidermal collagens constitute the primary structural components of the nematode cuticle and maintain cuticle elasticity and body wall-muscle attachment, defects in their synthesis and assembly directly compromise epidermal mechanical strength and barrier function [22]. The promoter region of the cuticle collagen gene *dpy-7* contains binding sites for epidermal cell-specific transcription factors [24]. Reduced *nhr-23* expression in *C. elegans* significantly decreases expression of the adult-specific collagen BLI-1 [13]. Genome-wide expression profiling identified a series of NHR-23-dependent target genes, revealing that genes down-regulated following *nhr-23* interference included multiple cuticle collagen genes and hedgehog signaling pathway components [25]. In *C. elegans*, *nhr-23* functions primarily in seam cells and hypodermal cells; its depletion causes structural abnormalities in the apical extracellular matrix, severely compromising cuticle barrier function [13]. RNAi of *Acnr1f* produces molting defect phenotypes resembling those caused by cuticle structural protein synthesis deficiencies, including disorganized cuticle structure, and epidermal detachment. DBDs in nematodes exhibit high conservation (≥75% identity), suggesting that NR1F-mediated regulation of epidermal gene expression represents an evolutionarily conserved function. We hypothesize that *Acnr1f* knockdown reduces transcription factor NR1F-mediated activation of downstream collagen genes, thereby compromising epidermal integrity.

Furthermore, GO enrichment analysis of DEGs from rat- versus mouse-derived larvae (Fig 2C) shared significant enrichment of terms related to cuticle structure, including “structural constituent of cuticle” (molecular function), “collagen trimer” (cellular component), and “molting cycle, collagen and cuticulin-based cuticle” (biological process). However, the DEGs from rat- versus mouse-derived larvae (Fig 2C) additionally showed enrichment in biological processes such as “embryo development,” “spermatogenesis,” and “adult lifespan”—categories not prominently represented in the *Acnr1f* RNAi dataset (Fig 4). This distinction suggests that, beyond the conserved NR1F/NHR-23-mediated cuticle regulatory program, worms adapted to different host environments (rat versus mouse) exhibit more pronounced divergence in overall developmental status, including reproductive and aging-related pathways. In contrast, the *Acnr1f* RNAi DEGs appear more restricted to the direct transcriptional targets of NR1F involved in cuticle biogenesis.

### LBD sequence divergence underlies *Ac*NR1F ligand specificity

Comparative analysis of nuclear receptor evolutionary function suggests that LBD structural conservation responds to oxysterol signaling molecules [26]. This conservation likely reflects the shared evolutionary origin of NHRs as ligand-activated transcription factors that transduce lipophilic signals into genomic responses. In details, the LBD region of *Ac*NR1F shares 47.9% identity with *C. elegans* NHR-23, 41.1% with *Drosophila* HR3, and 31–36% with human RORα/β/γ, indicating extensive remodeling of ligand specificity. Human RORs recognize diverse sterols—including cholesterol, cholesteryl sulfate, retinoic acid, and oxysterols—with isoform-specific preferences that activate distinct downstream pathways [27]. To identify potential ligands of *Ac*NR1F related to parasitic adaptation, we performed steroidomic profiling of infected brain and larvae from mice and rats. The abundant cholesterol, corticosterone, and 7α,25-dihydroxycholesterol had different levels between larvae from mice and rats. Moreover, these three steroids could bind the *Ac*NR1F-LBD and activate transcription.

Cholesterol is essential for *C. elegans* development and must be acquired exogenously [28,29]. The cholesterol level was higher in larvae from mouse than that from rat, however, the activity of enzymes in the pathway to catalyze the production of downstream metabolites is unknown, thus, the reason of such high level of cholesterol in infected mice remains elusive. Corticosterone levels were significantly elevated in both mouse-derived larvae and infected mouse brains compared to rat-derived counterparts. *A. cantonensis* infection enhances endogenous corticosteroid activity of mice to peripheral immunosuppression [30]. Infected mice had much more severe pathological changes than infected rat [31]. Thus, the higher level of corticosterone in infected mice may be used to enhance stress tolerance. 7α,25-dihydroxycholesterol was near or below detection limits in infected mouse brain but maintain the high level in the brain of rat infected with *A. cantonensis*. In mammals, 7α,25-dihydroxycholesterol regulates immune cell tissue localization, coordinating immune responses and maintaining immune homeostasis; dysregulation of its synthesis or function contributes to inflammation and autoimmune diseases [32]. These three ligands may be recruited to regulate expression patterns of *Ac*NR1F target genes, thereby influencing parasitic adaptation of *A. cantonensis* in rodents.

### *Ac*NR1F functions as a ligand-gated transcriptional switch associated with development

The observed collagen down-regulation in mouse-derived larvae can be explained by the following mechanism. Mice mount stronger immune responses than rats, subjecting mouse-derived worms to more intense immunological attack and causing more severe impairment of protein synthesis capacity, including reduced *Acnr1f* expression. Under severe immunological stress, immunosuppressive steroid of corticosterone is produced in large quantities in infected mice to enhance stress tolerance. Mouse-derived worms may utilize the abundant steroids to enhance the compensatory capacity of limited *Acnr1f*, thereby increasing collagen production (Fig 6). Nevertheless, the deficiency of 7α,25-dihydroxycholesterol, combined with overwhelming immunological stress, renders this compensatory response insufficient to achieve collagen expression levels comparable to rat-derived worms, resulting in inadequate collagen synthesis and developmental arrest. Thus, *Ac*NR1F functions as a ligand-gated transcriptional switch at the promoters of downstream genes to orchestrate developmental programs governing metabolism, reproduction, and tissue homeostasis. The differences in ligand availability, *Acnr1f* expression, and downstream gene expression between permissive and non-permissive hosts reflect the innate resistance mechanisms that inhibit larval development in non-permissive hosts.

**Fig 6:**
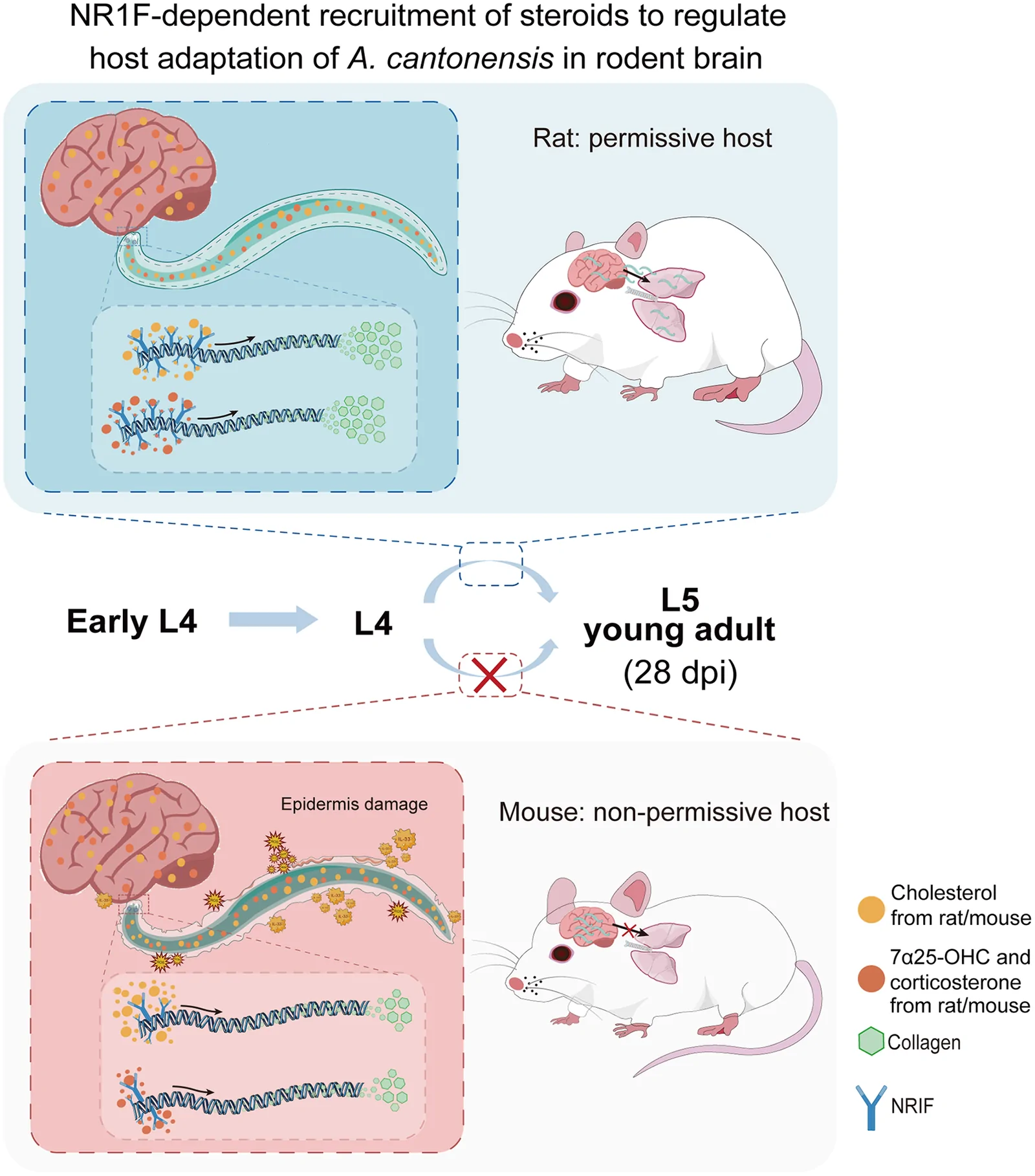
Schematic diagram of potential function of *Ac*NR1F in regulating host adaptation of *A. cantonensis* in rats and mice.

Therefore, *Ac*NR1F directly activates cuticle collagen genes to drive epidermal remodeling, thereby regulating parasitic adaptation of *A. cantonensis* in mice and rats. Up-regulation of epidermal structural proteins and proteases in rat-derived larvae suggests that compromised epidermal integrity in mouse-derived parasites contributes to developmental arrest.

## Conclusion

*Ac*NR1F functions as a ligand-dependent receptor that integrates host-derived steroidal signals (cholesterol, corticosterone, and 7α,25-dihydroxycholesterol) to activate target gene transcription through DNA recognition. By orchestrating developmental programs governing cuticle remodeling, metabolism, reproduction, and tissue homeostasis, *Ac*NR1F may serve as a critical molecular switch enabling *A. cantonensis* to adapt to different host environments. The failure of this adaptive mechanism in non-permissive hosts or upon genetic interference highlights *Ac*NR1F as a regulator of parasitic adaptation.

## Materials and methods

### Ethics statement

Sprague-Dawley (SD) rats and BALB/c mice were obtained from the Guangdong Experimental Animal Center. Animals were anesthetized at the experimental endpoint using 2% isoflurane and were sacrificed exclusively. All animal procedures conformed to the Chinese National Institute of Health Guide for the Care and Use of Laboratory Animals, and the protocol was approved by the South China Agricultural University Committee for Animal Research with approval number 2022F207.

### Worm collection and cultivation

The snail *Biomphalaria glabrata* was cultivated in our laboratory. *A. cantonensis* (Guangzhou strain) was maintained in our laboratory by a life cycle involving *B. glabrata* snails and SD rats. L1 larvae of *A. cantonensis* were obtained from rat feces at 45 dpi using the Baermann method. Isolated L1 larvae were used to infect *B. glabrata* snails that had been fasted for 48 h in advance. L3 larvae were collected from the snails. Rats were infected via oral gavage with approximately 50 L3 larvae. L4 and L5 larvae were collected from the brain of SD rats. L5 larvae and adult worms were collected from the lung of SD rats [1]. To study egg hatchability, adult worms were incubated in PBS buffer at 37 °C for 30 min for egg-laying. L1 larvae were collected after incubating the eggs in PBS buffer at 37 °C under 5% CO_2_ for 7 days [1]. Worm morphology was observed using a stereomicroscopy (Leica, Germany), and the size of eggs was calculated using Image J.

### Cloning and prokaryotic expression

Total RNA was extracted from adult worms using Trizol (Invitrogen, USA) and reverse transcribed into cDNA using the PrimeScript RT Master Mix (TaKaRa, Japan). *A. cantonensis nr1f* gene was amplified using Phanta Max Super-Fidelity DNA Polymerase (Vazyme, China) and cloned into pMD19-T and transformed in *Escherichia coli* DH5α. CDS sequence of *Acnr1f* gene was amplified using the primers of F: 5’- CGCGGATCCATGAGCTCTGCCCGTGTG-3’ and R: 5’-CCGGAATTCTGGATCAATCGAGAACAACTCC-3’. A 50 µL PCR reaction mixture was prepared by combining 25 µL of 2 × Phanta Max buffer, 1.0 µL of Phanta Max Super-Fidelity DNA Polymerase, 2.0 µL of each primer (forward and reverse, 10 µM each), 1.0 µL of template cDNA, 1.0 µL of dNTP Mix (10 µM each), and nuclease-free water to the final volume. The thermal cycling conditions consisted of an initial denaturation step at 95 °C for 3 min, followed by 35 cycles of denaturation at 95 °C for 15 seconds, annealing at 58 °C for 15 seconds, and extension at 72 °C for 90 seconds. The reaction was concluded with a final extension at 72 °C for 5 min to ensure complete product synthesis, followed by a 4 °C hold. *Acnr1f* gene was cloned into pET-28a for expression in *E. coli* Transetta (DE3) under 1 mM isopropylthio-β-galactoside (IPTG) at 16 °C for 18 h (Fig A in S1 Appendix). Recombinant *Ac*NR1F protein was purified using HisSep Ni-NTA Agarose Resin (Yeasen, China).

### Bioinformatic analysis

*Nr1f* genes were collected from human, mouse, and 17 nematode genomes from WormBase ParaSite release 18.0 (https://parasite.wormbase.org/) using OrthoFinder [33]. Conserved domain was identified with SMART (http://smart.embl-heidelberg.de/). A maximum-likelihood phylogeny of NR1F was constructed using domain sequence by RAxML-NG v1.1.0 [34]. Subcellular localization of the deduced NR1F proteins from *A. cantonensis* was predicted using Cell-PLoc 2.0 (http://www.csbio.sjtu.edu.cn/bioinf/Cell-PLoc-2/), and the presence of a signal peptide was evaluated with TargetP-2.0 (https://services.healthtech.dtu.dk/service.php?TargetP-2.0). Berkeley Drosophila Genome Project was used to predict promoter of collagen (http://www.fruitfly.org/seq_tools/promoter.html), JASPAR was used to predict transcription factor binding sites on genes (http://jaspar.genereg.net). Expression profiles of *Acnr1f* across developmental stages were extracted from our previously published RNA-seq data [35].

### Analysis of *Acnr1f* gene expression by chromogenic *in situ* hybridization

Adult worms of *A. cantonensis* were washed with PBS buffer and then fixed in 4% paraformaldehyde containing 0.1% Tween-20 at 4°C for 16 h. The samples were subjected to gradient dehydration using ethanol solutions of 50%, 70%, 95%, and 100% (each for 30 min), followed by two rounds of xylene clearing (each for 15 min). The samples were then embedded in paraffin and sectioned. The sections were digested with pepsin to increase probe accessibility. The sections were pre-hybridized at 37 °C for 2 h in a hybridization buffer containing 30% formamide, 5 × SSC, 0.1% Tween-20, and 1 mg/mL salmon sperm DNA to block non-specific binding sites. Digoxigenin (DIG)-labeled single-stranded DNA probes were added, and the sections were hybridized at 42 °C for 16 h. Following stringent post-hybridization washes using SSC buffer, signals were detected using an indirect immunoperoxidase method with biotinylated anti-DIG antibody, streptavidin-biotin complex, biotinylated horseradish peroxidase, and AEC chromogen. Sections were counterstained with hematoxylin, rinsed in water, and mounted. CISH signals were evaluated under an optical microscope.

### Identification of siRNA

Three siRNAs were biosynthesized by GenePharma (Suzhou, China). The sequences of siRNAs were shown in Table E in S1 Appendix. L5 larvae were used to evaluate the effects of siRNAs on *nr1f* gene expression. L5 larvae were divided into five groups: (i) wild-type (WT), (ii) 0.2 mM siRNA negative control (NC), (iii) 0.2 mM siRNA-*nr1f*-94, (iv) 0.2 mM siRNA-*nr1f*-894, and (v) 0.2 mM siRNA-*nr1f*-1219. After 24 h of incubation with siRNAs, fifty worms in each group were washed three times with PBS buffer and harvested for qRT-PCR.

### Real-time qPCR

Total RNA of *A. cantonensis* was extracted using Trizol (Invitrogen, USA) and reverse transcribed to cDNA using PrimeScript RT reagent Kit (Takara, Japan) after removing contaminating genomic DNA with DNase I (Sigma, USA). qPCR was performed using SYBR Green Real-Time PCR Master Mix (Toyobo, Japan) on a Light Cycler 480 Instrument II (Roche, Switzerland). The primers for *Acnr1f* were 5’- CCACCATCACAACAACCAAGTTATGC-3’ and 5’-GCGACTCCATAGGAATGCGAAGG-3’, the primers for *Acgapdh* were 5’-GCGAAGGCAGTTGGAAAGGTTATTC-3’ and 5’-CTGGTTTCTCAAGACGGCAGGTAAG-3’. A 20 µL qPCR reaction mixture was prepared by combining 10 µL of 2 × SYBR Green PCR Mix, 1.0 µL of each primer (forward and reverse, 10 µM each), 2.0 µL of template cDNA, and nuclease-free water to the final volume. The thermal cycling conditions consisted of an initial denaturation step at 95 °C for 30 seconds, followed by 40 cycles of denaturation at 95 °C for 5 seconds, annealing at 60 °C for 10 seconds, and extension at 72 °C for 15 seconds. Relative gene expression levels were calculated using the 2^(^−ΔΔ^Ct) method after normalization to gene *gapdh.*

### Lentivirus *nr1f*-894 and *nr1f-*1219 administration

Target gene-specific hairpin stem sequences were composed of siRNA sense sequences, loop sequences, siRNA antisense sequences, and polyA, and named shRNA. The shRNA sequences were as follows: siRNA-*Acnr1f-*894 sequences 5’-CTCGAGGCAAATCCACTTACTCAAATTCAAGAGATTTGAGTAAGTGGATTTGCTTTTTTGGATCC-3’ and shRNA-*Acnr1f*-1219 sequences 5’- CTCGAGGCTCAGAGAGTTTGGGATATTCAAGAGATATCCCAAACTCTCTGAGCTTTTTTGGATCC-3’. These sequences were biosynthesized (Sangon, China) and cloned into the miR-30a context of pGIPZ, between the 5′ and 3′ human miR-30a flanking sequences, downstream of the CMV-driven TurboGFP–IRES–PuroR cassette, so that TurboGFP, PuroR and the shRNAmir are transcribed as a single polycistronic mRNA. The primers of shRNAmir 5’-CAAGCCCGGTGCCTGAGTT-3’ and 5’-TGGCCGGCCGCATTAGTCTT-3’ were used to identify the shRNAmir sequences. LV particles containing shRNAmir were prepared and concentrated as described previously [21]. Virus titers were estimated as TCID50 (50% tissue culture infectious dose) using the Reed-Muench method.

### Parasite infection, recovery and transduction with lentivirus

Infective L3 larvae (iL3s) were obtained from infected *B. glabrata* at 28 dpi, and iL3s were washed with PBS buffer containing 0.45% glucose, 100 U/mL penicillin, 100 μg/mL streptomycin, 100 μg/mL gentamicin. Larvae were washed with serum-free medium (RPMI1640, 1.5% glucose, 2 mM L-glutamine, 100 U/mL penicillin, 100 μg/mL streptomycin, 100 μg/mL gentamicin, 20 mM HEPES) twice, and added to recombinant LV. Approximately 100 L3s were exposed to LV at multiplicities of infection (MOIs) of 400 in 1 mL of serum-free medium containing 10 μg/mL polybrene (Sigma, USA). Controls were L3s without virus (wild-type, WT) and L3s exposed to LV lacking a small hairpin structure (empty vector, EV). LV-containing culture medium for L3s was replaced with culture medium (RPMI1640, 2% FBS, 1.5% glucose, 2 mM L-glutamine, 100 U/mL penicillin, 100 μg/mL streptomycin, 100 μg/mL gentamicin, 20 mM HEPES) after 24 h of incubation, and interference effects of L3s were evaluated after an additional 48 h incubation. Approximately 100 L3s were used to infect 6-week-old female SD rats by gavage, and *A. cantonensis* were collected from rat brains at 28 dpi and from rat lungs at 50 dpi. Three biological replicates were included.

### SEM and TEM

For SEM observation, the 28 dpi and 50 dpi of worms from SD rats and the 28 dpi of worms from mice were washed using PBS buffer and were prefixed in 2.5% glutaraldehyde, postfixed in 1% OsO_4_, dehydrated through a graded series of ethanol (30%, 50%, 70%, 80%, 90%, 95%, and 100%), and then dehydrated in a Leica Model CDP 300 critical point dryer with liquid CO_2_. The samples were coated with platinum-palladium in ACE 600 ion sputter (Leica, Germany) and observed in EVO MA 15 (ZEISS, Germany). For TEM observation, the 28 dpi and 50 dpi of worms from SD rats were prefixed in 2.5% glutaraldehyde, postfixed in 1% OsO4, stained with uranyl acetate, dehydrated through a graded series of ethanol (30%, 50%, 70%, 85%, 95% and 100%), permeated and embedded with resin and acetone mixtures in different proportions (1:1 and 2:1). The samples were sliced after polymerization and examined with a Talos F200S transmission electron microscope (Thermo Fisher Scientific, USA).

### Masson staining

Six adult female worms were randomly selected and fixed in 4% paraformaldehyde at room temperature for 48 h. Fixed samples were embedded in paraffin and sectioned. Masson’s trichrome staining was performed using the Masson Trichrome Staining Kit according to the manufacturer’s instructions (Beyotime, China). Sections were deparaffinized, rehydrated, stained sequentially with hematoxylin, Ponceau-acid fuchsin, and light green, then dehydrated, cleared, and mounted with neutral balsam. Stained sections were examined under a light microscope and images were analyzed using ImageJ.

### Expression profile analyses

Total RNA was extracted from *A. cantonensis* from adult worms with interfering *nr1f* expression at 80 dpi. Total RNA was used for transcriptome sequencing using the Illumina NovaSeq 6000 high-throughput sequencing platform (Personalbio, China). RNA-seq data of adult worms with interfering *Acnr1f* expression were obtained. FastQC (v0.11.6) was used for quality control and Trim-galore (v0.6.6) was used to filter out low-quality reads. Reads were mapped to the reference genome (CNSA accession: CNP0000467) using HISAT2 (v2.1) [36]. We used the featureCount of Subread package (v1.6) to obtain the read count of genes [37]. Then, STAR (v2.7.9a) and RSEM (v1.3.3) were employed to calculate the fragments per kilobase million (FPKM) of the genes [38,39]. Differential expression analysis between groups was performed using DESeq2. Genes with |log_2_fold change| > 1 and adjusted *p*-value < 0.05 were considered significantly differentially expressed. TBtools (v1.098696) was used to visualize the expression level of genes in the heatmap [40].

### Co-expressed analysis of transcriptomic data

Weighted Gene Co-expression Network Analysis (WGCNA) (v1.74) [41] was performed on transcriptomic data of *A. cantonensis* collected at 14, 21, and 28 dpi from mice and rats and 55 dpi from rats. Hierarchical clustering of samples was conducted to detect outliers. A soft-thresholding power of β = 10 was selected to achieve scale-free topology. Adjacency and topological overlap matrices were subsequently calculated, and gene co-expression modules were identified by hierarchical clustering of the TOM dissimilarity using the dynamic tree cut method.

### Screening downstream genes of *Ac*NR1F from genomes

The *Ac*NR1F protein sequence was queried against the JASPAR database to identify transcription-factor binding profiles. The RORA motif (JASPAR matrix ID MA0071.1), exhibiting the lowest E-value, was selected as the candidate binding model. Promoter sequences ~2 kb upstream of the translation start site for all annotated genes were retrieved from the *A. cantonensis* genome (CNSA accession: CNP0000467). MEME was then employed to scan these promoters for putative *Ac*NR1F binding sites [42].

### Double luciferase report assays for DNA binding activity

Total DNA was extracted from adult worms of *A. cantonensis* using QIAGEN DNeasy Blood &Tissue Kit (Qiagen, Germany). Approximately 2000 bp promoter region in the upstream of 5’ UTR of *col-12* and *col-144* were amplified from total DNA and cloned into plasmids pGL3-Basic with restriction enzymes of *Kpn* I and *Sma* I (Fig C in S1 Appendix). The primers for *col-12-p* were 5’- GGGGTACCCCTCCACTGCACAAAACCGAA-3’ and 5’-TCCCCCGGGGGAACGAGAACGCAAAATGTAATGT-3’. The primers for *col-144-p* were 5’-GGGGTACCCCCAGGTGGAGAAAAAATAGAACA-3’ and 5’- TCCCCCGGGGGAGTGGGTGAGAAACTCAGCATAG -3’. *Acnr1f* was cloned in pcDNA3.1 using *Bam*H I and *Eco*R I to obtain pcDNA3.1-*Acnr1f* (Fig D in S1 Appendix). HEK-293T cells were co-transfected with the reporter construct (pGL3-Basic-*col-12*-p or pGL3-Basic-*col-144*-p), the expression vector pcDNA3.1-*Acnr1f*, and the Renilla luciferase internal control pRL-TK using Lipofectamine 2000. As a blank control, cells were transfected with the empty pGL3-Basic vector together with pcDNA3.1-*Acnr1f* and pRL-TK. Firefly luciferase and sea kidney luciferase activities were assayed according to the instructions of Dual-luciferase reporter gene assay system kit (Promega, USA).

### LC-MS/MS detection of steroids

Mice were infected via oral gavage with approximately 30 L3 larvae. L5 larvae of *A. cantonensis* were collected from rat and mouse brains at 28 dpi. And the corresponding 50 mg uninfected rat and mouse brain tissues, infected rat and mouse brains were collected at 28 dpi. All samples were stored at −80 °C until analysis. After thawing, 50 mg wet weight of larvae and rodent brain tissues were homogenized in 400 µL of methanol using a tissue homogenizer on ice, followed by vortexing for 10 min on ice to extract steroids. The mixture was centrifuged at 12,000 rpm for 5 min at 4 °C. Approximately 370 µL supernatant was dried under nitrogen at 20 °C, redissolved in 100 µL methanol, vortexed for 5 min, and re-centrifuged (12,000 rpm, 3 min, 4 °C). The final 80 µL supernatant was transferred for UPLC-MS/MS analysis.

Steroid profiling was performed on an ExionLC AD UPLC system coupled to a QTRAP 6500 + LC-MS/MS system (SCIEX, USA) equipped with a Phenomenex Kinetex C18 column (1.7 µm, 100 × 2.1 mm) maintained at 40 °C. Mobile phase A was acetonitrile/water (30:70, v/v) containing 0.04% (v/v) acetic acid, and mobile phase B was acetonitrile/isopropanol (50:50, v/v) containing 0.04% (v/v) acetic acid. The gradient program was as follows: 0–1 min, 5% B; 1–10 min, linear increase from 5% to 90% B; 10-12.5 min, hold at 90% B; 12.5-15 min, ramped back to 5% B. The flow rate was 0.35 mL/min. The TurboIonSpray source was operated in positive-ion mode with the following settings: source temperature 550 °C, ion spray voltage 5,500 V, curtain gas 35 psi. Data were acquired in multiple reaction monitoring mode. Steroids were identified and quantified by comparison to a custom-built spectral library of 43 steroid standards and their derivatives.

### Double luciferase report assays for ligand binding activity

The ligand-binding domain of *Ac*NR1F (NR1F-LBD) was amplified using Phanta Max Super-Fidelity DNA Polymerase and cloned into plasmid pFN26A (Fig E in S1 Appendix). The primers for amplification of NR1F-LBD were 5’-CCGGAATTCCGCATCCCTAGACTTCCAGAAT-3’ and 5’- GCTCTAGAAGCTTCGGTTTCAGATGGG-3’ with restriction enzymes of *EcoR* I and *Xba* I. The recombinant plasmid pFN26A-NR1F-LBD was co-transfected with pGL4.35 into HEK-293T cells using Lipofectamine 2000. The working concentration of 1 μM, 5 μM, 10 μM, 20 μM, 50 μM, 75 μM, 100 μM of cholesterol, corticosterone, and 7α,25-dihydroxycholesterol in 100% alcohol were added in culture medium of HEK-293T cells. The blank control group was treated identically to the other experimental groups, except that no ligand was added. Firefly and Renilla luciferase activities were measured sequentially using Dual Luciferase Reporter Assay System (Promega, USA). Each experiment was performed in triplicate and repeated independently three times. Data were analyzed using Prism 9 (GraphPad Software, USA).

### Statistical analysis

Data were presented as mean ± SD. Comparisons were made by one-way ANOVA followed by the appropriate post-hoc test, or by unpaired t-test where applicable. *P* < 0.05 was considered statistically significant. All analyses were performed in Prism 9.0 (GraphPad Software, USA).

## Supporting information

S1 Appendix Fig A. Gel electrophoresis of PCR fragment of *Acnr1f* gene. Fig B. Functional annotations of the up-regulated and down-regulated expression of genes in *A. cantonensis* from mice and rats at 28 dpi. Fig C. Gel electrophoresis of PCR fragments of collagen gene promoter region. Fig D. Gel electrophoresis of CDS region of *Acnr1f* and pcDNA3.1 vector after *Bam*H I and *Eco*R I digestion. Fig E. Gel electrophoresis of PCR fragments of the ligand-binding domain of *Ac*NR1F. Table A. Expression pattern of *Acnr1f* and *Acgapdh* across developmental stages of *A. cantonensis* based on RNA-seq (Log2FPKM). Table B. GO enrichment of the main DEGs in larvae from mouse brains at 28 dpi compared to larvae from rat brains at 28 dpi. Table C. Expression pattern of *Acnr1f* and *Acgapdh* from mouse and rat-derived *A. cantonensis* (Log2FPKM). Table D. Genome-wide analysis identified 918 potential downstream targets of *Ac*NR1F, which were predominantly enriched within the co-expressed genes of the turquoise module identified by WGCNA (FPKM). Table E. siRNA sequences for RNAi of *Acnr1f* gene. Table F. RNAi effects of three siRNAs on adult worms by qPCR analysis (n = 3). Table G. *Acnr1f* gene transcription in F0 generation of adult worms treated with lentivirus LV-*Acnr1f*-894 and LV-*Acnr1f*-1219 at MOI of 400 by qPCR analysis (n = 3). Table H. Number of L4 larvae (21 dpi) from rat brains (n = 4). Table I. Percentage of L5 larvae delaying in rat brains treatment with LV-*Acnr1f*-894 (n = 4). Table J. Number of L1 larvae from per gram of rat feces (n = 3). Table K. Number of F1 generation of eggs laid from per female (n = 3). Table L. Length of the eggs (μm) (n = 15). Table M. Hatching rate of F1 generation of eggs to L1 larvae from F0 generation of adult worms (n = 3). Table N. Masson staining of F0 generation of adult worms for observing cuticle thickness (μm) (n = 15). Table O. GO enrichment of DEGs of F0 generation of adult worms between EV and RNAi groups. Table P. Expression pattern of 36 collagens in the F0 generation of adult worms with Log2(FPKM)>1 among WT, EV and RNAi groups. Table Q. Identification of two *collagen* genes as downstream targets of *Ac*NR1F by dual-luciferase reporter assays (n = 3). Table R. Expression pattern of two *collagen* genes across developmental stages of *A. cantonensis* (Log2FPKM). Table S. LC-MS/MS analysis of steroids contents in the brain of SD rats and BALB/c mice and larvae at 28 dpi (n = 6, ng/g dry weight). Table T. Binding activities of cholesterol, corticosterone and 7α25-OHC to *Ac*NR1F assessed by dual-luciferase reporter assays.
(DOCX)

S1 Rawgel Raw image A. This is the original gel electrophoresis image for Fig 1B. Raw image B. The right image is the original gel electrophoresis image for Fig 3C. Raw image C. This is the original gel electrophoresis image for Fig 3D. Raw image D. This is the original gel electrophoresis image for Fig A Raw image E. This is the original gel electrophoresis image for Fig CA. Raw image F. This is the original gel electrophoresis image for Fig CB. Raw image G. This is the original gel electrophoresis image for Fig D. Raw image H. This is the original gel electrophoresis image for Fig E.
(DOCX)
