# Supplementary material for: AcNR1F orchestrates epidermal remodeling to enable host adaptation in Angiostrongylus cantonensis
Source: PLoS Pathog. 2026 Oct 12;22(10):e1014691. doi: 10.1371/journal.ppat.1014691 (PMC13657317; doi:10.1371/journal.ppat.1014691)
Supplement: Supplementary file 1 [file ppat.1014691.s001.docx]

**S1 Appendix:**

**Figure A. Gel electrophoresis of PCR fragment of *Acnr1f* gene**

M: DNA marker; 1. Amplification of *Acnr1f* CDS fragment PCR.

**Figure B Functional annotations of the up-regulated and down-regulated expression of genes in *A. cantonensis* from mice and rats at 28 dpi.**

**Figure C Gel electrophoresis of PCR fragments of collagen gene promoter region**

M: DNA marker; (A) *Accol-12*-promotor; (B) *Accol-144*-promotor.

**Figure D Gel electrophoresis of CDS region of *Acnr1f* and pcDNA3.1 vector after *Bam*H I and *Eco*R I digestion**

M: DNA marker; 1. CDS region of *Acnr1f*; 2. pcDNA3.1 vector.

**Figure E Gel electrophoresis of PCR fragment of the ligand-binding domain of *Ac*NR1F**

M: DNA marker; 1. PCR amplification of the ligand-binding domain of *Ac*NR1F.

**Table A Expression pattern of *Acnr1f* and *Acgapdh* across developmental stages of *A. cantonensis* based on RNA-seq (Log2FPKM).**

| Gene | L1 | L3 | L4 | L5 | AF | AM |
| --- | --- | --- | --- | --- | --- | --- |
| *Acnr1f* | 2.16 | 0 | 2.70 | 7.08 | 8.49 | 4.99 |
| *Acgapdh* | 11.62 | 10.95 | 11.14 | 11.51 | 11.58 | 11.69 |

Note: L1, First stage larvae; L3, Third stage larvae; L4, Fourth stage larvae; L5, Fifth stage larvae; AF, Adult female; AM, Adult male.

**Table B GO enrichment of the main DEGs in larvae from mouse brains at 28 dpi compared to larvae from rat brains at 28 dpi.**

| **Term** | **ID** | **Input number** | **Background number** | **P-value** |
| --- | --- | --- | --- | --- |
| Cytoplasm | GO:0005737 | 217 | 1958 | 1.78475159653e-60 |
| Hydrolase activity | GO:0016787 | 141 | 831 | 2.64690095588e-58 |
| Metal ion binding | GO:0046872 | 160 | 1381 | 2.25136536053e-46 |
| Membrane | GO:0016020 | 381 | 6222 | 5.22823079223e-45 |
| Nucleus | GO:0005634 | 204 | 2275 | 1.43907066395e-43 |
| ATP binding | GO:0005524 | 119 | 909 | 4.57441949163e-39 |
| Phosphorylation | GO:0016310 | 83 | 478 | 3.9743294658e-35 |
| Kinase activity | GO:0016301 | 82 | 468 | 5.81454342772e-35 |
| Peptidase activity | GO:0008233 | 54 | 227 | 5.48643296404e-29 |
| Proteolysis | GO:0006508 | 65 | 365 | 2.70774606875e-28 |
| Innate immune response | GO:0045087 | 48 | 264 | 1.44052585828e-21 |
| Molting cycle, collagen and cuticulin-based cuticle | GO:0018996 | 28 | 68 | 6.80675519882e-21 |
| Nucleotide binding | GO:0000166 | 86 | 887 | 1.71314282472e-20 |
| Transmembrane transport | GO:0055085 | 52 | 451 | 1.31875951179e-15 |
| Determination of adult lifespan | GO:0008340 | 36 | 251 | 1.009276363e-13 |
| Transferase activity | GO:0016740 | 74 | 942 | 2.05042455688e-13 |
| Collagen trimer | GO:0005581 | 16 | 55 | 1.12945602497e-10 |
| Spermatogenesis | GO:0007283 | 16 | 56 | 1.41414709848e-10 |
| Protein binding | GO:0005515 | 74 | 1095 | 1.5057096398e-10 |
| Oxidoreductase activity | GO:0016491 | 39 | 389 | 2.08323811225e-10 |
| Structural constituent of cuticle | GO:0042302 | 25 | 166 | 2.30460008564e-10 |
| Cell-cell junction | GO:0005911 | 11 | 22 | 9.66773363718e-10 |
| Cytoskeleton | GO:0005856 | 29 | 261 | 5.30806785788e-09 |
| Embryo development ending in Birth or egg hatching | GO:0009792 | 31 | 305 | 1.08565196339e-08 |
| Lipid metabolic process | GO:0006629 | 23 | 207 | 1.96902161864e-07 |
| Cell surface | GO:0009986 | 10 | 34 | 3.26007882118e-07 |
| P granule | GO:0043186 | 13 | 70 | 5.86552906035e-07 |
| Pseudopodium | GO:0031143 | 8 | 22 | 1.34303411776e-06 |
| Neuron projection | GO:0043005 | 21 | 201 | 1.62796120884e-06 |
| Oviposition | GO:0018991 | 13 | 84 | 3.58821092075e-06 |

**Table C Expression pattern of *Acnr1f* and *Acgapdh* from mouse- and rat-derived *A. cantonensis* (Log2FPKM).**

| Gene | L3 | R14D | R21D | R28D | M14D | M21D | M28D |
| --- | --- | --- | --- | --- | --- | --- | --- |
| *Acnr1f* | 0.18 | 0.7 | 1.95 | 3.5 | 0.74 | 2.03 | 2.29 |
| *Acgapdh* | 9.75 | 10.24 | 10.57 | 10.94 | 10.52 | 10.41 | 10.64 |

Note: L3, Third stage larvae; R14D, Larvae from rat brains at 14 dpi; R21D, Larvae from rat brains at 21 dpi; R28D, Larvae from rat brains at 28 dpi; M14D, Larvae from mouse brains at 14 dpi; M21D, Larvae from mouse brains at 21 dpi; M28D, Larvae from mouse brains at 28 dpi.

**Table D Genome-wide analysis identified 918 potential downstream targets of *Ac*NR1F, which were predominantly enriched within the co-expressed genes of the turquoise module identified by WGCNA (FPKM).**

| **Transcript_id** | **M14B** | **R14B** | **M21B** | **R21B** | **M28B** | **R28B** | **R28L** | **R55L** | **SwissprotName** |
| --- | --- | --- | --- | --- | --- | --- | --- | --- | --- |
| Angca_10659 | 27955.24 | 31719.51 | 38796.65 | 21885.66 | 13996.09 | 22178.69 | 11885.28 | 526.19 | dpy-13 |
| Angca_09844 | 524.32 | 652.12 | 503.59 | 255.79 | 129.41 | 307.55 | 142.07 | 16.7 | dpy-13 |
| Angca_08234 | 28.25 | 28.19 | 52.49 | 46.54 | 15.4 | 8.82 | 3.74 | 0.27 | col-144 |
| Angca_02486 | 0.8 | 0.96 | 0.91 | 1.1 | 0.62 | 1.5 | 2.49 | 3.38 | col-80 |
| Angca_05058 | 1.32 | 1.62 | 0.96 | 1.22 | 1.69 | 0.61 | 1.33 | 5.11 | dpy-2 |
| Angca_04727 | 1951.33 | 1899.46 | 1975.98 | 2051.57 | 1953.38 | 2242.72 | 3298.75 | 6901.92 | pfn-1 |
| Angca_00446 | 3675.87 | 3589.8 | 3794.69 | 4819.75 | 3617.42 | 2983.23 | 2106.33 | 646.65 | far-1 |
| Angca_03796 | 3062.97 | 3600.07 | 2371.89 | 2317.97 | 1389.97 | 2248.67 | 1828.89 | 1259.75 | dlc-1 |
| Angca_12450 | 1425.44 | 1900.29 | 1752.64 | 1561.23 | 1505.9 | 1863.18 | 1529.79 | 510.98 | ttr-46 |
| Angca_06470 | 1117.72 | 1470.47 | 1521.77 | 1355.55 | 1276.1 | 1256.65 | 1264.03 | 645.11 | - |
| Angca_11178 | 4.97 | 9.24 | 15.03 | 4.98 | 3.87 | 698.65 | 1241.66 | 22435.64 | vit-2 |
| Angca_06416 | 1348.71 | 1487.1 | 1301.55 | 1829.52 | 1429.97 | 1367.67 | 995.55 | 985.94 | GLRX |
| Angca_00641 | 1170.24 | 1182.61 | 1122.04 | 1128.33 | 1295.1 | 1104.84 | 915.95 | 1032.9 | eft-3 |
| Angca_05377 | 570.89 | 635.78 | 552.08 | 561.77 | 658.62 | 654.53 | 725.26 | 796.28 | rpl-2 |
| Angca_00420 | 951.65 | 1214.94 | 972.79 | 1380.3 | 731.15 | 1017.42 | 693.56 | 476.38 | SERP2 |
| Angca_04569 | 488.71 | 573.4 | 503.93 | 504.53 | 458.82 | 509.59 | 521.57 | 424.74 | - |
| Angca_02577 | 369.9 | 286.65 | 368.56 | 431.01 | 371.8 | 412.75 | 505.35 | 721.71 | pkm |
| Angca_01334 | 441.68 | 675.58 | 685.16 | 699.98 | 640.5 | 627.42 | 485.02 | 177.01 | ttr-33 |
| Angca_12851 | 345.88 | 335.57 | 354.97 | 355.04 | 363.46 | 395.81 | 476.85 | 501.28 | cct-4 |
| Angca_03791 | 485.92 | 530.41 | 475.69 | 587.84 | 517.25 | 483.67 | 426.52 | 375.83 | C23G10.2 |
| Angca_01272 | 270.15 | 230.35 | 248.06 | 288.42 | 165.69 | 323.77 | 420.22 | 508.92 | ubc-13 |
| Angca_09275 | 446.21 | 375.27 | 365.15 | 332.06 | 467.36 | 363.96 | 397.87 | 284.64 | mutd-1 |
| Angca_12175 | 57.4 | 45.17 | 65.81 | 77.02 | 104.56 | 129.66 | 394.41 | 1362.12 | car-1 |
| Angca_12091 | 350.01 | 383.03 | 315.5 | 291.66 | 361.01 | 344.74 | 392.04 | 491.42 | mdh-1 |
| Angca_13409 | 377.71 | 446.46 | 366.53 | 394.39 | 257.84 | 506.73 | 373.03 | 131.66 | his-3 |
| Angca_08682 | 227.81 | 342.22 | 186.5 | 247.43 | 262.67 | 309.18 | 357.53 | 533.6 | clns1a |
| Angca_10184 | 458.25 | 503.95 | 427.26 | 447.21 | 519.03 | 397.32 | 350.31 | 324.47 | asg-2 |
| Angca_11691 | 408.65 | 388.31 | 497.53 | 561.88 | 546.7 | 549 | 332.38 | 17.02 | - |
| Angca_07716 | 285.01 | 254.35 | 252.79 | 257.53 | 227.38 | 258.97 | 316.43 | 308.56 | cat2 |
| Angca_07813 | 363.48 | 309.42 | 286.2 | 352.24 | 275.94 | 320.58 | 299.01 | 154.27 | PEPCK |
| Angca_03548 | 139.14 | 98.13 | 143.41 | 135.72 | 149.82 | 190.61 | 292.88 | 516.86 | RAD23B |
| Angca_02189 | 86.48 | 77.01 | 170.68 | 146.38 | 109.03 | 525.28 | 291.43 | 618.4 | cah-5 |
| Angca_12912 | 223.71 | 251.98 | 204.19 | 229.18 | 224.48 | 217.56 | 288.92 | 354.12 | icd-1 |
| Angca_08848 | 163.05 | 177.21 | 187.21 | 209.18 | 234.38 | 257.55 | 288.83 | 551.69 | Tktl2 |
| Angca_11205 | 0.31 | 0.23 | 4.35 | 2.8 | 5.09 | 79.14 | 283.63 | 865.49 | cbd-1 |
| Angca_00797 | 252.93 | 248.8 | 275.16 | 310.26 | 261.41 | 290.83 | 283.2 | 415.21 | - |
| Angca_03026 | 434.28 | 355.68 | 364.3 | 470.29 | 495.21 | 441.91 | 261.29 | 68.34 | PRKAB2 |
| Angca_00364 | 259.88 | 238.26 | 243.02 | 237.61 | 285.42 | 176.73 | 251.34 | 393.47 | rsp-1 |
| Angca_05214 | 257.98 | 262.35 | 204 | 241.97 | 234.54 | 238.76 | 250.19 | 345.06 | F01G4.6 |
| Angca_11996 | 224.69 | 231.05 | 186.78 | 220.27 | 287.6 | 172.39 | 243.01 | 464.23 | epg-4 |
| Angca_01442 | 159.94 | 138.91 | 147.82 | 171.28 | 174.53 | 159.21 | 228.7 | 267.18 | Myef2 |
| Angca_07926 | 148.19 | 204.53 | 187.3 | 173.68 | 123.95 | 241.75 | 223.63 | 413.99 | fat-2 |
| Angca_07675 | 34.96 | 49.29 | 14.35 | 18.06 | 17.25 | 72.78 | 211.31 | 695.13 | cpg-3 |
| Angca_04703 | 17.12 | 20.49 | 80.83 | 46.18 | 62.18 | 119.68 | 207.33 | 312.06 | baf-1 |
| Angca_08386 | 109.26 | 111.13 | 112.62 | 128.67 | 165.49 | 133.81 | 207.11 | 320.81 | rab-18 |
| Angca_09923 | 182.79 | 171.66 | 177.37 | 174.76 | 183.37 | 183.22 | 203.43 | 104.96 | prps2 |
| Angca_10930 | 452.33 | 672.5 | 543.74 | 767.12 | 306.03 | 342.9 | 200.5 | 85.2 | asp-6 |
| Angca_00842 | 183.66 | 186.72 | 192.92 | 188.59 | 160.85 | 153.11 | 196.04 | 277.65 | LSM3 |
| Angca_08613 | 1.53 | 2.53 | 18.5 | 9.98 | 17.2 | 83.08 | 194.43 | 617.18 | pos-1 |
| Angca_08945 | 40.7 | 56.61 | 55.7 | 66.75 | 42.65 | 102.88 | 190.8 | 909.1 | ttr-52 |
| Angca_07464 | 130.22 | 157.21 | 164.78 | 163.12 | 209.24 | 157.92 | 183.73 | 270.83 | Rsf1 |
| Angca_01183 | 298.16 | 358.09 | 380.29 | 275.26 | 191.93 | 269.35 | 181.12 | 8.53 | C28H8.5 |
| Angca_00695 | 66.89 | 69.52 | 86.12 | 105.5 | 92.48 | 112.33 | 178.67 | 279.48 | dram1 |
| Angca_03868 | 511.39 | 409.74 | 541.92 | 551.95 | 336.44 | 329.65 | 173 | 119.06 | atx1 |
| Angca_11773 | 83.33 | 78.59 | 89.11 | 85.22 | 98.75 | 125.75 | 168.66 | 264.8 | ubxn-6 |
| Angca_01666 | 153.91 | 159.33 | 172.67 | 156.49 | 135.01 | 164.05 | 164.24 | 227.38 | rep-1 |
| Angca_05682 | 46.61 | 79.5 | 96.83 | 79.69 | 54.26 | 54.84 | 163.1 | 149.68 | POLR2D |
| Angca_04759 | 55.02 | 52.47 | 54.43 | 69.54 | 66.13 | 83.15 | 137.29 | 237.74 | cyb-3 |
| Angca_04640 | 87.53 | 87.09 | 85.44 | 96.59 | 99.52 | 96.88 | 136.41 | 227.39 | snap-29 |
| Angca_12085 | 93.63 | 104.29 | 97.9 | 87.31 | 125.98 | 165.17 | 132.34 | 174.49 | cni-1 |
| Angca_07405 | 37.87 | 39.54 | 48.7 | 52.12 | 56.83 | 64.76 | 132.27 | 125.9 | UBR7 |
| Angca_00877 | 126.69 | 133.06 | 115.03 | 114.63 | 133.22 | 124.15 | 131 | 146.59 | F44G4.1 |
| Angca_03623 | 84 | 95.88 | 81.17 | 95.91 | 87.15 | 90.63 | 128.21 | 106.08 | phb-2 |
| Angca_12408 | 92.04 | 98.99 | 97.82 | 103.67 | 117.99 | 87.75 | 127.98 | 138.27 | Gpn3 |
| Angca_09570 | 80.36 | 67.4 | 76.63 | 82.94 | 99.37 | 107.32 | 127.38 | 152.86 | JNK-1 |
| Angca_01943 | 17.34 | 12.1 | 27.53 | 28.92 | 31.38 | 56.93 | 126.91 | 560.39 | cgh-1 |
| Angca_01522 | 106.35 | 87.97 | 92.75 | 101.02 | 134.47 | 133.29 | 124.57 | 176.49 | scpl-3 |
| Angca_06019 | 96.64 | 64.09 | 70.17 | 86.7 | 93.52 | 98.91 | 123.62 | 159.02 | csn-4 |
| Angca_04130 | 103.74 | 81.84 | 91.61 | 115.34 | 111.97 | 100.92 | 120.02 | 119.55 | DNAJC3 |
| Angca_02795 | 118.11 | 113.92 | 104.14 | 107.8 | 134.54 | 88.21 | 119.58 | 185.33 | rsp-3 |
| Angca_10663 | 166.41 | 305.91 | 243.38 | 255.85 | 263.06 | 220.55 | 113.57 | 104.24 | eppl-1 |
| Angca_06465 | 45.45 | 45.19 | 60.15 | 45.97 | 35.56 | 76.79 | 110.21 | 143.27 | his-3 |
| Angca_07567 | 85.33 | 84.69 | 78.48 | 90.6 | 63.81 | 84.91 | 105.12 | 137.42 | rab-6.2 |
| Angca_02275 | 94.17 | 90.57 | 71.77 | 76.43 | 79.44 | 78.03 | 104.65 | 121.63 | NDUFV1 |
| Angca_05839 | 0.17 | 0.13 | 4.86 | 2.87 | 5.34 | 29 | 98.03 | 445.01 | Lrp2 |
| Angca_07488 | 92.18 | 96.21 | 81.34 | 92.74 | 93.64 | 77.03 | 93.79 | 148.58 | tin-13 |
| Angca_05291 | 20.3 | 22.47 | 27.81 | 20.24 | 19.79 | 78.28 | 93.5 | 112.81 | mxl-2 |
| Angca_03990 | 70.57 | 76.54 | 74.21 | 74.86 | 77.43 | 69.06 | 91.92 | 86.19 | rpt-3 |
| Angca_10391 | 116.84 | 101.86 | 135.56 | 135.4 | 75.79 | 117.3 | 90.62 | 20.08 | TMEM47 |
| Angca_04350 | 34.9 | 47.44 | 53.16 | 46.22 | 26.04 | 62.71 | 87.36 | 101.57 | his-48 |
| Angca_09156 | 72.86 | 71.39 | 60.96 | 65.11 | 64.1 | 47.83 | 86.83 | 103.36 | rsp-6 |
| Angca_07784 | 62.54 | 74.09 | 67.52 | 85.12 | 96.24 | 82.58 | 86.19 | 98.95 | Exosc1 |
| Angca_12950 | 93.32 | 73.07 | 75.24 | 90.75 | 77.26 | 87.66 | 86.07 | 132.16 | aco-2 |
| Angca_08812 | 114.45 | 106.67 | 101.85 | 108.35 | 102.3 | 84.43 | 84.19 | 75.86 | tric-1B.1 |
| Angca_12986 | 48.06 | 56.24 | 65.35 | 52.28 | 61.49 | 62.39 | 82.54 | 78.11 | EMC2 |
| Angca_01608 | 76.61 | 48.26 | 53.67 | 67.43 | 64.47 | 64.59 | 82.45 | 151.69 | slc39a1 |
| Angca_04424 | 50.07 | 45.62 | 52.93 | 55.14 | 36.43 | 60.84 | 82.3 | 70.71 | jamp-1 |
| Angca_13374 | 31.55 | 34.01 | 32.12 | 36.59 | 35.88 | 61.61 | 81.9 | 148.9 | C35D10.10 |
| Angca_08123 | 65.29 | 60.1 | 57.73 | 54.69 | 88.51 | 67.9 | 81.71 | 91.01 | smg-8 |
| Angca_00784 | 75.47 | 63.93 | 56.16 | 69.21 | 53.36 | 70.3 | 81.71 | 109.19 | lin-36 |
| Angca_09516 | 112.99 | 116.65 | 101.79 | 84.8 | 116.62 | 95.12 | 81.05 | 52.05 | pex2 |
| Angca_03948 | 49.52 | 50.1 | 51 | 48.05 | 47.24 | 48.67 | 80.61 | 228.61 | T22D1.3 |
| Angca_07853 | 56.63 | 59.46 | 63.39 | 61.03 | 67.28 | 64.88 | 79.67 | 112.49 | GOLGA7 |
| Angca_12045 | 44.02 | 40.26 | 39.93 | 48.22 | 39.13 | 64.48 | 79.45 | 118.66 | RABEP2 |
| Angca_01555 | 83.95 | 66.57 | 77.07 | 59.36 | 82.52 | 58.44 | 78.88 | 137.37 | NDUFA13 |
| Angca_09818 | 47.77 | 49.37 | 50.8 | 35.99 | 13.3 | 42.28 | 78.8 | 141.26 | lsy-27 |
| Angca_07178 | 34.8 | 42.36 | 51.64 | 44.65 | 42.37 | 58.55 | 77.67 | 114.66 | UBE2J1 |
| Angca_04005 | 48.35 | 54.95 | 48.46 | 61.29 | 48.2 | 64.3 | 77.46 | 97.91 | GAF1 |
| Angca_07792 | 36.39 | 31.55 | 40.51 | 42.44 | 44.82 | 63.96 | 77.25 | 100.62 | stam-1 |
| Angca_11112 | 46.84 | 48.74 | 49.04 | 60.42 | 56.84 | 57.01 | 77.05 | 119.87 | PDPK1 |
| Angca_05209 | 80.09 | 68.26 | 66.04 | 80.93 | 64.38 | 68.19 | 76.11 | 37.84 | enpl-1 |
| Angca_08510 | 50.35 | 54.51 | 58.47 | 75.4 | 63.21 | 70.67 | 75.3 | 91.62 | AP3S2 |
| Angca_05185 | 28.92 | 21.51 | 31.65 | 36.09 | 42.85 | 53.81 | 74.62 | 150.67 | egl-10 |
| Angca_13460 | 31.33 | 33.81 | 38.15 | 40.71 | 56.84 | 53.13 | 72.74 | 103.97 | ARF6 |
| Angca_02154 | 29.55 | 26.32 | 37.52 | 36.88 | 37.81 | 50.48 | 72.57 | 80.28 | Y37H9A.3 |
| Angca_01956 | 48.22 | 39.15 | 48.56 | 63.38 | 80.01 | 67.12 | 71.87 | 116.51 | PLB1 |
| Angca_07477 | 62.11 | 70.17 | 63.21 | 65.84 | 72.64 | 58.93 | 71.76 | 93.86 | CBG07973 |
| Angca_03074 | 50.43 | 48.87 | 52.97 | 58.94 | 53 | 55.2 | 71.53 | 82.88 | lex-1 |
| Angca_02477 | 85.38 | 74.2 | 69.11 | 67.51 | 48.52 | 77.68 | 70.04 | 11.07 | - |
| Angca_10649 | 33.16 | 29.93 | 35.36 | 38.86 | 50.59 | 52.81 | 69.19 | 115.75 | wapl-1 |
| Angca_04612 | 58.64 | 61.55 | 51.71 | 51.73 | 62.41 | 52.7 | 69.04 | 90.74 | His3.3A |
| Angca_13412 | 82.12 | 76.72 | 75.39 | 60.59 | 71.26 | 79.43 | 68.59 | 34.63 | his-3 |
| Angca_12092 | 56.26 | 62.09 | 72.78 | 73.23 | 61.87 | 72.38 | 68.34 | 31.53 | EPHX1 |
| Angca_06120 | 84.23 | 90.54 | 86.16 | 95.05 | 81.05 | 68.88 | 68.22 | 46.81 | pat-6 |
| Angca_09543 | 34.22 | 34.48 | 39.59 | 37.54 | 37.87 | 51.88 | 68.13 | 82.82 | Usp4 |
| Angca_12176 | 71.04 | 78.26 | 69.92 | 83.78 | 99.98 | 64.1 | 68.13 | 160.76 | Tmem254 |
| Angca_06420 | 59.41 | 43.07 | 48.95 | 75.77 | 70.1 | 55.76 | 68.07 | 119.11 | gnas |
| Angca_04357 | 51.09 | 46.18 | 43.32 | 62.52 | 66.73 | 40.38 | 66.54 | 73.78 | NOB1 |
| Angca_01373 | 49.94 | 53.45 | 58.72 | 64.31 | 57.94 | 55.57 | 65.88 | 37.27 | nst-1 |
| Angca_04559 | 45.09 | 31.09 | 40.58 | 40.31 | 44.48 | 50.49 | 65.81 | 97.18 | Blzf1 |
| Angca_06281 | 42.44 | 38.99 | 42.91 | 48.73 | 58.63 | 52.85 | 65.46 | 83.43 | Xpac |
| Angca_07096 | 68.86 | 68.38 | 60.02 | 68.96 | 66.86 | 55.47 | 65.11 | 36.34 | Abcf1 |
| Angca_03568 | 147.75 | 162.09 | 170.89 | 168.8 | 74.03 | 88.73 | 63.95 | 65.78 | - |
| Angca_05052 | 39.65 | 36.01 | 43.33 | 44.17 | 44.03 | 48.51 | 63.77 | 81.53 | wdfy-2 |
| Angca_09139 | 44.79 | 39.88 | 42.57 | 56.56 | 62.57 | 48.67 | 63.52 | 79.38 | map-2 |
| Angca_09721 | 100.6 | 90.82 | 82.2 | 100.6 | 84.98 | 78.33 | 62.46 | 22.27 | Slc3a1 |
| Angca_12267 | 71.31 | 60.66 | 60.26 | 69.47 | 60.06 | 56.01 | 61.08 | 41.82 | Ahsa1 |
| Angca_08934 | 34.36 | 28.79 | 31.34 | 25.54 | 26.04 | 42.44 | 59.91 | 157.78 | Gfpt1 |
| Angca_05186 | 24.87 | 21.91 | 30.83 | 28.7 | 34.21 | 48.47 | 59.51 | 67.53 | F28C1.3 |
| Angca_13022 | 35.36 | 32.15 | 34.37 | 43.5 | 47.18 | 47.01 | 59.3 | 68.19 | Usp7 |
| Angca_10818 | 42.29 | 32.29 | 40.2 | 45.45 | 47.77 | 47.34 | 58.94 | 78.55 | Cpsf6 |
| Angca_11841 | 39.88 | 38.12 | 43.39 | 44.18 | 34.42 | 50 | 58.69 | 149.5 | mfsd5 |
| Angca_04228 | 57.01 | 43.72 | 35.82 | 32.01 | 37.56 | 31.61 | 58.69 | 6.81 | his-3 |
| Angca_11425 | 55.92 | 49.97 | 52.66 | 47.48 | 49.55 | 45.34 | 58.47 | 45.31 | B0416.5 |
| Angca_07210 | 36.84 | 34.06 | 36.03 | 45.54 | 59.54 | 48.46 | 58.33 | 131.5 | odr-4 |
| Angca_08274 | 48.21 | 69.25 | 65.34 | 60.22 | 56.73 | 50.12 | 57.23 | 28.07 | bre-5 |
| Angca_02580 | 38.48 | 31.47 | 36.3 | 43.72 | 44.03 | 39.61 | 57.19 | 71.79 | fitm-2 |
| Angca_08840 | 41.57 | 48.87 | 40.2 | 37.46 | 59.16 | 44.3 | 57.18 | 60.8 | rrp-1 |
| Angca_07821 | 8.39 | 8.71 | 18.44 | 19.96 | 19.4 | 36.3 | 57.01 | 81.24 | cyb-1 |
| Angca_10415 | 51.16 | 37.27 | 36.34 | 42.07 | 44.37 | 50.72 | 56.36 | 56.1 | Vps36 |
| Angca_03795 | 34.08 | 27.51 | 32.22 | 35.3 | 41.01 | 36.85 | 56.17 | 92.82 | png-1 |
| Angca_10911 | 20.76 | 20.49 | 28.64 | 33.68 | 30.3 | 36.68 | 55.98 | 109.66 | unc-84 |
| Angca_01447 | 59.56 | 64.62 | 59.19 | 60.34 | 47.87 | 49.45 | 55.89 | 34.59 | SMN1 |
| Angca_00648 | 37.84 | 33.97 | 34.51 | 36.64 | 36.69 | 46.01 | 55.59 | 105.85 | Pitpna |
| Angca_01291 | 36.74 | 33.9 | 27.73 | 32.51 | 33.64 | 45.96 | 55.22 | 93.48 | slc39a14 |
| Angca_06210 | 0.13 | 0.1 | 0.09 | 0.09 | 0.09 | 4.31 | 54.72 | 1498.03 | ASP |
| Angca_00397 | 51.33 | 58.2 | 51.12 | 50.69 | 55.89 | 50.73 | 53.22 | 84.77 | NNT |
| Angca_11589 | 30.31 | 25.14 | 35.92 | 36.37 | 35.85 | 39.02 | 53.16 | 175.39 | ced-1 |
| Angca_08584 | 33.54 | 25.3 | 33.69 | 41.43 | 54.67 | 47.01 | 53.06 | 81.93 | FBXO25 |
| Angca_01070 | 27.08 | 26.83 | 31 | 30.48 | 35.2 | 30.21 | 52.85 | 56.82 | pcf-11 |
| Angca_07212 | 42.89 | 35.38 | 35.7 | 47.04 | 50.43 | 43.83 | 52.77 | 147.65 | pan-1 |
| Angca_00753 | 40.74 | 37.95 | 38.42 | 41.81 | 33.91 | 39.32 | 52.52 | 46.15 | byn-1 |
| Angca_07824 | 38.94 | 41.6 | 41.4 | 58.79 | 41.86 | 49.18 | 52.34 | 75.69 | Eif2b2 |
| Angca_02177 | 74.77 | 67.36 | 72 | 65.59 | 62.83 | 62.05 | 51.94 | 41.81 | sem-5 |
| Angca_06815 | 48.67 | 46.47 | 41.67 | 44.02 | 36.5 | 41.04 | 51.92 | 75.11 | F27D4.1 |
| Angca_08704 | 13.53 | 10.43 | 18.25 | 23.51 | 24.31 | 34.89 | 51.48 | 74.75 | cah-2 |
| Angca_00318 | 35.87 | 34.43 | 35.79 | 43.25 | 45.07 | 39.1 | 51.38 | 69.11 | hars-1 |
| Angca_12237 | 24.73 | 18.99 | 28.24 | 36.98 | 32.65 | 40.41 | 51.23 | 48.64 | gck-3 |
| Angca_02409 | 28.99 | 32.02 | 32.63 | 32.25 | 31.8 | 45.39 | 51.16 | 56.11 | pxl-1 |
| Angca_07714 | 4.23 | 4.89 | 3.26 | 6.3 | 9.3 | 13.01 | 50.84 | 197.55 | marchf3 |
| Angca_09838 | 37.33 | 42.36 | 38.04 | 33.47 | 36.38 | 39.13 | 50.44 | 46.35 | dnj-21 |
| Angca_11631 | 1.43 | 1.01 | 6.56 | 3.02 | 5.82 | 21.98 | 50.44 | 127.88 | egg-5 |
| Angca_09246 | 42.86 | 36.65 | 39.09 | 42.97 | 48.45 | 44.36 | 50.26 | 56.81 | DCTN4 |
| Angca_12975 | 29.66 | 23.73 | 28.92 | 26.83 | 29.75 | 40.55 | 50.18 | 68.8 | bicd-1 |
| Angca_03303 | 27.38 | 23.77 | 25.72 | 28.22 | 28.14 | 33.25 | 49.51 | 79.42 | ddi2 |
| Angca_04476 | 41.45 | 54.56 | 55.65 | 55.25 | 42.91 | 37.96 | 49.47 | 39.59 | aat-6 |
| Angca_05208 | 40.09 | 41.94 | 42.11 | 38.91 | 43.85 | 45.47 | 49.02 | 52.02 | deps-1 |
| Angca_01099 | 25.26 | 22.18 | 25.48 | 28.72 | 26.62 | 33.53 | 48.92 | 72.49 | Fdx2 |
| Angca_01652 | 37.88 | 30.11 | 35.16 | 36.76 | 33.71 | 43.48 | 47.65 | 57.67 | tag-325 |
| Angca_02658 | 29.33 | 24.29 | 31.05 | 28.94 | 30.12 | 36.93 | 47.06 | 45.15 | HDAC1 |
| Angca_06183 | 3.33 | 3.6 | 18.22 | 12.39 | 15.52 | 33.91 | 46.8 | 64.4 | baf-1 |
| Angca_03927 | 29.23 | 23.56 | 27.44 | 33.24 | 40.82 | 41.36 | 46.73 | 60.38 | KCTD5 |
| Angca_11382 | 41.71 | 45.14 | 37.94 | 39.12 | 40.34 | 38.15 | 46.06 | 70.37 | Macrod2 |
| Angca_03132 | 36.65 | 35.58 | 41.09 | 49.03 | 42.89 | 38.59 | 46 | 20.49 | Sf3a3 |
| Angca_01547 | 25.68 | 23.86 | 26.79 | 24.16 | 32.71 | 38.98 | 45.58 | 68.48 | GPR158 |
| Angca_04925 | 18.45 | 16.65 | 22.27 | 20.57 | 21.32 | 31.84 | 45.44 | 94.78 | Tesk2 |
| Angca_09637 | 74.01 | 87.47 | 76.1 | 62.87 | 42.96 | 50.53 | 44.44 | 11.61 | MFSD8 |
| Angca_13049 | 19.97 | 17.77 | 21.35 | 23.98 | 32.05 | 30.35 | 44.38 | 40.48 | KIF2A |
| Angca_04756 | 34.08 | 28.89 | 22.64 | 27.64 | 39.59 | 37.23 | 44.23 | 51.32 | smad2 |
| Angca_00348 | 32.95 | 34.37 | 39.19 | 37.41 | 36.67 | 39.99 | 44.18 | 57.3 | sptl-3 |
| Angca_02783 | 15.58 | 13.22 | 25.6 | 14 | 12.55 | 23.45 | 44.18 | 73.66 | atgl-1 |
| Angca_05648 | 28.58 | 29.11 | 28.47 | 31.92 | 28.17 | 37.03 | 44.11 | 63.35 | rpt-4 |
| Angca_09481 | 21.88 | 20.92 | 28.52 | 27.72 | 23.02 | 34.12 | 43.76 | 58.67 | nito |
| Angca_09493 | 51.2 | 41.62 | 44.49 | 49.42 | 49.74 | 48.59 | 43.1 | 31.35 | bec-1 |
| Angca_01186 | 19.22 | 8.51 | 17.61 | 27.63 | 25.62 | 25.7 | 42.8 | 138.37 | neur |
| Angca_07804 | 25.07 | 24.64 | 25.76 | 24.32 | 22.6 | 26.84 | 42.6 | 44.77 | lem-3 |
| Angca_08074 | 24.74 | 12.37 | 23.86 | 24.27 | 30.17 | 27.58 | 42.59 | 70.45 | EDEM2 |
| Angca_08488 | 43.01 | 35.52 | 32.49 | 50.5 | 48.82 | 56.71 | 42.48 | 49.24 | lysmd2 |
| Angca_04990 | 41.71 | 46.86 | 39.3 | 36.53 | 38.49 | 34.49 | 41.85 | 24.11 | Dnajc9 |
| Angca_04271 | 30.12 | 22.56 | 28.3 | 30.44 | 25.33 | 35.2 | 41.8 | 74.32 | ced-6 |
| Angca_05303 | 27.85 | 32.27 | 32.46 | 41.61 | 34.73 | 36.17 | 41.68 | 78.18 | Ncam1 |
| Angca_11120 | 20.37 | 22.88 | 24.24 | 22.84 | 24.64 | 26.97 | 41.48 | 69.63 | mcm-6 |
| Angca_12818 | 37.44 | 46.37 | 52.81 | 43.45 | 42.65 | 31.97 | 41.11 | 17.7 | Trim3 |
| Angca_03175 | 15.92 | 15.68 | 27.04 | 23.33 | 27.76 | 33.67 | 41.04 | 71.43 | gld-2 |
| Angca_04950 | 19.3 | 17.49 | 20.93 | 27.83 | 28.11 | 34.22 | 40.57 | 40.53 | pink-1 |
| Angca_10499 | 40.31 | 42.01 | 52.41 | 32.27 | 55.27 | 43.55 | 39.6 | 77.97 | AGXT |
| Angca_10380 | 28.29 | 26.71 | 48.41 | 32.24 | 20.94 | 20.65 | 39.05 | 140.25 | mex3b |
| Angca_05894 | 19.42 | 20.3 | 13.19 | 17.76 | 22.31 | 23.17 | 38.86 | 87.64 | tent5c |
| Angca_09173 | 41.04 | 31.05 | 27.28 | 34.16 | 30.56 | 25.47 | 38.61 | 54.22 | ric-3 |
| Angca_10703 | 22.71 | 19.57 | 23.54 | 29.99 | 32.36 | 23.88 | 38.38 | 61.61 | C3orf33 |
| Angca_05072 | 31.49 | 14.73 | 25.31 | 33.18 | 31.33 | 30 | 38.27 | 75.29 | glna-2 |
| Angca_00493 | 23.07 | 19.66 | 21.57 | 24.99 | 32.47 | 27.28 | 38.21 | 43.68 | EXD3 |
| Angca_00774 | 39.24 | 50.02 | 50.79 | 45.42 | 47.35 | 48.11 | 38.11 | 92.81 | - |
| Angca_08478 | 33.25 | 34.81 | 41.62 | 39.27 | 34.34 | 36.21 | 38.03 | 43.37 | bli-4 |
| Angca_03733 | 26.39 | 28.37 | 26.37 | 25.77 | 27.63 | 28.66 | 38.01 | 35.17 | AIFM1 |
| Angca_08777 | 30.2 | 36.3 | 32.66 | 28.79 | 35.07 | 33.32 | 37.91 | 45.24 | MRPL14 |
| Angca_04784 | 28.48 | 19.15 | 23.96 | 32.89 | 23.92 | 31.25 | 37.88 | 37.13 | trm-1 |
| Angca_09319 | 29.09 | 20.53 | 20.55 | 26.27 | 33.39 | 33.27 | 37.71 | 124.49 | CHN48 |
| Angca_04894 | 26.28 | 22.06 | 25.06 | 26.18 | 27.87 | 27.54 | 37.68 | 36.39 | gyf-1 |
| Angca_04864 | 32.52 | 29.56 | 29.46 | 27.63 | 31.19 | 30.56 | 37.44 | 39.16 | pgam-5 |
| Angca_08008 | 29.26 | 40.03 | 39.48 | 30.37 | 29.98 | 35.31 | 36.94 | 10.11 | ugt-50 |
| Angca_12133 | 31.7 | 31.59 | 31.19 | 30.98 | 31.04 | 31.77 | 36.92 | 35.58 | MRPS35 |
| Angca_09130 | 9.44 | 6.68 | 8.02 | 6.63 | 11.53 | 18.44 | 36.89 | 71.44 | amt-2 |
| Angca_03180 | 26.07 | 26.41 | 28.64 | 27.95 | 26.53 | 28.55 | 36.8 | 47.96 | Cln3 |
| Angca_04338 | 0.7 | 0.51 | 10.47 | 5.74 | 8.59 | 21.2 | 36.65 | 37.67 | TAF3 |
| Angca_07035 | 20.1 | 25.81 | 22.22 | 26.42 | 34.67 | 26.94 | 36.42 | 42.28 | ska-1 |
| Angca_00217 | 20.48 | 19.32 | 19.31 | 20.97 | 37.32 | 21.83 | 35.99 | 52.35 | alkbh6 |
| Angca_13026 | 18.91 | 16.07 | 21.66 | 26.52 | 32.17 | 24.6 | 35.9 | 35.47 | DNAJC11 |
| Angca_07479 | 18.43 | 15.4 | 17.56 | 20.86 | 21.92 | 24.41 | 35.64 | 54.6 | gsk-3 |
| Angca_07817 | 27.65 | 29.92 | 29.46 | 28.17 | 27.67 | 30 | 35.58 | 38.24 | Ubc2 |
| Angca_10788 | 26.95 | 22.1 | 20.61 | 27.15 | 27.71 | 24.64 | 35.3 | 62.67 | kcc-3 |
| Angca_08581 | 0.21 | 0.14 | 4.91 | 2.85 | 3.6 | 13.16 | 35.02 | 159.73 | mex-5 |
| Angca_12858 | 50.15 | 31.72 | 40.62 | 52.13 | 22.12 | 45.3 | 34.73 | 16.22 | MGAM |
| Angca_03217 | 28.14 | 24.35 | 20.84 | 23.29 | 26.82 | 27.88 | 34.41 | 47.74 | immp1l |
| Angca_02144 | 50.94 | 46.64 | 54.07 | 54.13 | 54.01 | 44.1 | 34.25 | 19.52 | EAF2 |
| Angca_07920 | 33.21 | 32.8 | 27.94 | 31.51 | 29.56 | 28.8 | 34.14 | 18.84 | ZNF207 |
| Angca_05380 | 18.79 | 19.61 | 23.55 | 27.78 | 22.22 | 26.17 | 34.09 | 31.41 | abhd12 |
| Angca_07282 | 19.36 | 25.67 | 15.97 | 12.34 | 15.82 | 18.97 | 33.9 | 66.38 | aspdh |
| Angca_06862 | 19.42 | 18.68 | 22.85 | 21.57 | 29.21 | 25.19 | 33.8 | 44.78 | Afdn |
| Angca_12906 | 23.9 | 23.5 | 20.86 | 21.99 | 27.66 | 23.32 | 33.74 | 65.54 | Prss30 |
| Angca_10348 | 31.93 | 37.22 | 35.67 | 34.69 | 31.91 | 32.63 | 33.67 | 26.38 | Inpp5f |
| Angca_02405 | 11.53 | 11.38 | 15.57 | 16.03 | 17.7 | 21.02 | 33.63 | 34.19 | R05D3.2 |
| Angca_09267 | 37.29 | 46.99 | 40.6 | 30.81 | 29.15 | 28.17 | 33.56 | 23.52 | slc35b4 |
| Angca_08450 | 12.16 | 12 | 17.92 | 12.88 | 15.35 | 20.07 | 33.55 | 44.69 | CHKB |
| Angca_05511 | 26.76 | 20.73 | 23.74 | 26.81 | 24.53 | 20.93 | 33.24 | 68.88 | B0303.7 |
| Angca_01246 | 34.91 | 37.15 | 34.95 | 43.27 | 42.55 | 35.93 | 33.14 | 55.2 | PRPSAP2 |
| Angca_11772 | 17.88 | 17.36 | 21.06 | 22.02 | 27.69 | 24.73 | 32.84 | 37.79 | Apeh |
| Angca_03201 | 22.56 | 22.23 | 22.02 | 26.34 | 22.35 | 25.05 | 32.78 | 43.43 | slc25a46 |
| Angca_07866 | 12.88 | 12.05 | 15.57 | 17.44 | 19.16 | 18.65 | 32.76 | 39.65 | nud-2 |
| Angca_06727 | 31.95 | 32.56 | 32.04 | 32.74 | 31.94 | 30.57 | 32.76 | 46.2 | RAB2 |
| Angca_02261 | 23.42 | 23.09 | 22.27 | 24.7 | 25.03 | 24.82 | 32.64 | 34.85 | RPN2 |
| Angca_07819 | 32.63 | 28.69 | 25.94 | 28.01 | 30.29 | 29.83 | 32.26 | 33.17 | SESN3 |
| Angca_10738 | 19.55 | 16.9 | 19.95 | 23.53 | 27.09 | 24.58 | 32.08 | 30.69 | cogc-8 |
| Angca_08699 | 46.36 | 55.58 | 51.74 | 37.92 | 25.41 | 32.17 | 31.97 | 5.39 | AVT1D |
| Angca_03222 | 26.03 | 13.97 | 18.91 | 29.9 | 31.11 | 29.5 | 31.95 | 35.16 | mig-10 |
| Angca_02608 | 26.15 | 24.31 | 24.66 | 24.61 | 26.18 | 30.94 | 31.9 | 48.74 | pamn-1 |
| Angca_04265 | 11.98 | 13.92 | 16.84 | 16.14 | 13.54 | 24.04 | 31.88 | 60.64 | POR |
| Angca_00173 | 9.71 | 7.15 | 13.38 | 10.95 | 15.98 | 21.06 | 31.81 | 55.42 | mys-1 |
| Angca_01500 | 34.68 | 44.31 | 38.14 | 32.33 | 28.42 | 30.99 | 31.7 | 19.89 | maea |
| Angca_11534 | 29.75 | 32.27 | 28.41 | 25.4 | 25.43 | 24.85 | 31.59 | 19.61 | nsun-5 |
| Angca_12326 | 16.18 | 16.24 | 14.28 | 24.07 | 19.84 | 25.53 | 31.49 | 32.51 | B0035.15 |
| Angca_08409 | 16.1 | 13.61 | 20.41 | 19.94 | 19.45 | 25.68 | 31.17 | 35.54 | Kiaa0319l |
| Angca_05845 | 20.08 | 17.98 | 23.46 | 28.08 | 18.78 | 25.07 | 31.15 | 49.07 | TM9SF4 |
| Angca_05598 | 27.93 | 31.41 | 25.55 | 29.7 | 28.78 | 27.21 | 31.06 | 39.93 | mrps-7 |
| Angca_06840 | 1.88 | 1.73 | 3.27 | 3.37 | 4.35 | 8.22 | 31.05 | 323.75 | egg-6 |
| Angca_04103 | 35.19 | 31.77 | 28.59 | 28.65 | 23.45 | 25.07 | 30.85 | 91.37 | LRRC58 |
| Angca_06053 | 43.57 | 34.23 | 41.89 | 45.82 | 37.07 | 34.17 | 30.83 | 29.31 | cher |
| Angca_02014 | 24.95 | 24.68 | 23.62 | 30.29 | 26.67 | 25.43 | 30.76 | 34.17 | C35D10.1 |
| Angca_12956 | 20.74 | 20.94 | 21.66 | 23.18 | 18.71 | 27.46 | 30.62 | 45.79 | XPR1 |
| Angca_03167 | 30.03 | 27.28 | 23.46 | 22.83 | 30.54 | 26.25 | 30.59 | 39.9 | Rdh14 |
| Angca_10016 | 8.03 | 10.18 | 13.34 | 12.95 | 18.41 | 18.82 | 30.53 | 43.28 | ndc80 |
| Angca_12679 | 38.74 | 40.66 | 34.35 | 35.66 | 30.89 | 35.2 | 30.44 | 23.21 | - |
| Angca_11538 | 21.16 | 23.51 | 26.04 | 28.37 | 25.93 | 19.2 | 30.37 | 29.54 | gpr89-b |
| Angca_13053 | 53.39 | 74.84 | 79.23 | 67.9 | 65.67 | 19.73 | 30.32 | 16.31 | Tango2 |
| Angca_09376 | 8.15 | 0 | 0 | 0 | 4.84 | 0 | 30.08 | 144.34 | rab1 |
| Angca_10811 | 50.85 | 38.2 | 43.16 | 50.88 | 49.51 | 46.61 | 29.54 | 9.99 | sorb-1 |
| Angca_08745 | 34.64 | 30.7 | 25.45 | 30.51 | 33.57 | 30.51 | 29.52 | 19.62 | pfut-1 |
| Angca_09219 | 16.94 | 18.47 | 17.43 | 19.81 | 24.22 | 15.39 | 29.4 | 31.07 | Tsr3 |
| Angca_00079 | 0.49 | 0.61 | 4.7 | 2.12 | 3.03 | 11.54 | 29.28 | 74.69 | CYP4V2 |
| Angca_12609 | 20.99 | 20.66 | 24.94 | 19.47 | 29.7 | 24.73 | 29.17 | 30.26 | Tbc1d2b |
| Angca_08821 | 20.45 | 17.34 | 16.11 | 19.7 | 20.48 | 21 | 29.16 | 47.26 | hmp-2 |
| Angca_09259 | 18.69 | 17.19 | 18.87 | 19.96 | 24.55 | 20.38 | 29.13 | 39.78 | GK25604 |
| Angca_06224 | 17.98 | 20.17 | 16.24 | 19.94 | 27.15 | 18.63 | 29.06 | 32.5 | ttr-46 |
| Angca_07024 | 4.63 | 3.79 | 5.98 | 4.5 | 7.47 | 8.75 | 28.86 | 74.74 | cpg-4 |
| Angca_02635 | 21.14 | 14.14 | 22.9 | 21.34 | 15.49 | 22.87 | 28.44 | 39.42 | mig-23 |
| Angca_08410 | 18.91 | 16.91 | 20.72 | 20.89 | 24.54 | 24.56 | 28.34 | 28.68 | Trappc9 |
| Angca_00639 | 10.46 | 9.34 | 12.06 | 14.48 | 19.31 | 15.9 | 27.99 | 35.32 | F31E3.4 |
| Angca_04006 | 31.4 | 32.07 | 29.17 | 32.25 | 33.52 | 30.6 | 27.66 | 21.62 | Trmt10c |
| Angca_09770 | 28.13 | 23.34 | 24.94 | 35.98 | 26.95 | 20.88 | 27.53 | 54.12 | algn-11 |
| Angca_07908 | 9.38 | 8.04 | 9.38 | 8.59 | 12.17 | 17.52 | 27.47 | 85.89 | Ptpn12 |
| Angca_00777 | 14.25 | 11.63 | 11.11 | 14.1 | 12.1 | 14.81 | 27.31 | 144.13 | UGP2 |
| Angca_06451 | 9.45 | 7.92 | 13.8 | 16.59 | 18.48 | 20.17 | 27.15 | 47.28 | wago-1 |
| Angca_00216 | 9.47 | 7.05 | 13.07 | 13.09 | 16.68 | 15.15 | 27.1 | 54.19 | Foxn1 |
| Angca_10973 | 28.94 | 28.85 | 25.25 | 29.96 | 24.72 | 25.1 | 27.09 | 38.06 | AGAP012208 |
| Angca_07577 | 11.58 | 15.07 | 11.18 | 10.62 | 10.2 | 16.79 | 26.51 | 90.1 | mrp-7 |
| Angca_09547 | 20.65 | 17.78 | 12.76 | 15.42 | 15.19 | 19.59 | 26.36 | 35.22 | IPMK |
| Angca_02430 | 18.49 | 12.57 | 19.71 | 21.17 | 26.53 | 22.51 | 26.33 | 31.64 | Slk |
| Angca_07958 | 17.31 | 13.23 | 15 | 17.43 | 16.76 | 20.13 | 26.28 | 43.18 | DCAF7 |
| Angca_02145 | 16.49 | 13.8 | 16.92 | 18.63 | 19.76 | 21.25 | 26.24 | 27.6 | Sacm1l |
| Angca_01964 | 19.6 | 17.73 | 19.83 | 19.51 | 26.78 | 15.79 | 26.01 | 63.17 | RYBP |
| Angca_04650 | 16.35 | 12.19 | 14.3 | 19.21 | 28.15 | 19.18 | 25.97 | 28.18 | srpa-68 |
| Angca_10575 | 38.43 | 48.77 | 57.8 | 37.96 | 26.95 | 19.42 | 25.83 | 18.24 | pch-2 |
| Angca_09148 | 22.04 | 21.68 | 19.85 | 23.11 | 20.77 | 19.34 | 25.74 | 44.55 | pezo-1 |
| Angca_07349 | 20.02 | 12.87 | 15.26 | 18.77 | 22.4 | 19.14 | 25.64 | 24.9 | Son |
| Angca_00693 | 30.33 | 21.64 | 24.71 | 33.28 | 28.71 | 27.33 | 25.62 | 8.83 | nfki-1 |
| Angca_09964 | 28.63 | 33.71 | 31.12 | 28.21 | 25.39 | 24.18 | 25.59 | 18.44 | unc-119 |
| Angca_09479 | 14.5 | 14.73 | 16.8 | 16.34 | 17.15 | 20.64 | 25.5 | 23.97 | tut-1 |
| Angca_06819 | 6.15 | 5.38 | 6.72 | 7.27 | 9.89 | 12.97 | 25.45 | 114.87 | Ptchd3 |
| Angca_08483 | 23.54 | 24.16 | 21.33 | 25.68 | 25.97 | 20.98 | 25.39 | 40.52 | lid-1 |
| Angca_12963 | 10.77 | 6.68 | 12.77 | 16.18 | 23.83 | 22.14 | 25.39 | 46.24 | egl-45 |
| Angca_09480 | 15.61 | 17.81 | 18.84 | 18.44 | 18.78 | 22.22 | 25.37 | 31.25 | IFRD2 |
| Angca_00795 | 33.29 | 36.48 | 30.04 | 29.33 | 22.15 | 27.03 | 25.31 | 18.88 | F54F2.9 |
| Angca_04999 | 5.67 | 4.9 | 6.5 | 5.32 | 9.6 | 10.57 | 24.73 | 80.47 | gmps-1 |
| Angca_11639 | 38.05 | 33.62 | 30.04 | 32.93 | 26.52 | 30.58 | 24.65 | 13.06 | hs6st1b |
| Angca_01938 | 12.24 | 12.98 | 13.34 | 13.33 | 14.62 | 17.81 | 24.48 | 30.35 | Bm1_28595 |
| Angca_11717 | 13.81 | 10.82 | 14.67 | 13.99 | 14.78 | 18.52 | 24.46 | 36.37 | PBRM1 |
| Angca_08294 | 17.68 | 14.85 | 15.42 | 17.28 | 22.67 | 16.98 | 24.32 | 24.63 | MPHOSPH10 |
| Angca_00896 | 13.97 | 12.34 | 15.16 | 15.32 | 16.9 | 19.17 | 24.19 | 43.4 | Tex2 |
| Angca_13433 | 23.15 | 21.93 | 29.19 | 28.94 | 23.61 | 25.58 | 23.74 | 13.34 | faah-4 |
| Angca_08183 | 19.99 | 18.11 | 15.63 | 18.92 | 19.31 | 18.07 | 23.7 | 24.42 | kics2 |
| Angca_11433 | 9.49 | 7.09 | 10.21 | 11.51 | 16.18 | 15.76 | 23.66 | 36.25 | spri |
| Angca_13264 | 22.1 | 16.56 | 23.24 | 24.97 | 19.3 | 30.37 | 23.58 | 1.88 | sup-10 |
| Angca_08404 | 25.5 | 29.12 | 31.65 | 26.87 | 29.9 | 25.07 | 23.24 | 4.69 | ttr-2 |
| Angca_09416 | 18.9 | 14.27 | 15.91 | 19.17 | 20.75 | 20.67 | 23.23 | 24.82 | Nup54 |
| Angca_10796 | 13.45 | 11.31 | 12.46 | 14.49 | 16.94 | 16.93 | 23.17 | 35.83 | Slc41a1 |
| Angca_11272 | 5.19 | 7.83 | 9.07 | 9.82 | 12.93 | 9.71 | 23.11 | 19.67 | pri-1 |
| Angca_01135 | 11.21 | 7.55 | 12.72 | 13.15 | 17.65 | 16.59 | 23.02 | 24.17 | dre-1 |
| Angca_06223 | 7.32 | 5.6 | 7.46 | 9.59 | 17.27 | 12.84 | 22.79 | 74.84 | Acsl5 |
| Angca_00992 | 18.66 | 21.58 | 23.04 | 20.43 | 19.3 | 18.33 | 22.62 | 22.24 | PTP4A3 |
| Angca_01115 | 32.34 | 59.38 | 54.67 | 50.47 | 23.63 | 38.78 | 22.61 | 6.71 | bre-4 |
| Angca_05092 | 9.41 | 8.35 | 12.21 | 10.45 | 6.21 | 14.26 | 22.46 | 48.01 | C41D11.9 |
| Angca_09804 | 7.48 | 7.55 | 9.93 | 11.72 | 14.97 | 16.05 | 22.22 | 27.01 | ERCC2 |
| Angca_05792 | 14.46 | 18.33 | 20.91 | 16.48 | 21.98 | 17.12 | 22.13 | 26.06 | MMAB |
| Angca_01003 | 16 | 16.04 | 24.97 | 17.5 | 16.44 | 15.56 | 22.08 | 69.34 | rgs-7 |
| Angca_09939 | 22.01 | 34.92 | 34.03 | 29.23 | 18.02 | 27.21 | 21.97 | 15.35 | nrf-6 |
| Angca_03073 | 24.76 | 20.88 | 21.74 | 24.41 | 26.02 | 18.74 | 21.91 | 11.71 | Rbm34 |
| Angca_11055 | 18.09 | 17.8 | 15.86 | 15.47 | 15.23 | 21.83 | 21.9 | 20.6 | MICU3 |
| Angca_08295 | 19.96 | 19.35 | 14.42 | 14.43 | 18.21 | 16.88 | 21.86 | 34.7 | bcl-7 |
| Angca_06778 | 17.42 | 16.64 | 15.11 | 16.3 | 20.58 | 16.26 | 21.83 | 24.45 | COASY |
| Angca_01200 | 7.48 | 6.7 | 12.04 | 10.04 | 13.78 | 14.2 | 21.68 | 42.87 | PELI1 |
| Angca_04457 | 15.47 | 14.93 | 15.5 | 17.96 | 20.06 | 17.71 | 21.67 | 24.38 | usp33 |
| Angca_07175 | 13.25 | 10.61 | 7.29 | 11.66 | 9.69 | 8.37 | 21.57 | 27.56 | Rrh |
| Angca_02311 | 11.67 | 9.39 | 12.04 | 11.49 | 13.03 | 16.27 | 21.56 | 18.71 | nol6 |
| Angca_00139 | 26.04 | 17.6 | 18.16 | 18.12 | 11.35 | 16.26 | 21.56 | 47.5 | MET |
| Angca_02225 | 59.84 | 37.73 | 55.69 | 67.21 | 37.28 | 45.69 | 21.51 | 0.69 | ttr-2 |
| Angca_03349 | 10.09 | 6.95 | 7.54 | 9.6 | 8.83 | 20.69 | 21.25 | 22.46 | clip-1 |
| Angca_11727 | 23.91 | 26.23 | 22.51 | 19.37 | 19.65 | 22.24 | 21.18 | 1.5 | dyf-5 |
| Angca_10443 | 24.89 | 43.93 | 45.95 | 25.33 | 20.93 | 29.54 | 21.12 | 16.27 | hgo-1 |
| Angca_11088 | 5.08 | 4.91 | 6.75 | 6.16 | 4.74 | 12.29 | 21.03 | 66.11 | cya-1 |
| Angca_06506 | 14.44 | 11.08 | 13.98 | 14.87 | 17.43 | 16.79 | 20.99 | 30.9 | mtm-3 |
| Angca_09194 | 15.99 | 32.91 | 36.93 | 23.79 | 33.93 | 29.3 | 20.88 | 9.67 | Prss36 |
| Angca_02873 | 15.09 | 11.28 | 16.63 | 17.57 | 12.36 | 12.91 | 20.84 | 45.09 | CBL |
| Angca_06848 | 11.35 | 11.61 | 10.24 | 13.01 | 15.16 | 15.98 | 20.65 | 50.96 | MCRS1 |
| Angca_10974 | 8.73 | 14.99 | 10.49 | 12.6 | 13.58 | 10.69 | 20.57 | 33.24 | Stk16 |
| Angca_01722 | 10.55 | 9.36 | 11.65 | 12.51 | 12.21 | 13.69 | 20.53 | 23.46 | E01A2.2 |
| Angca_00419 | 4.33 | 1.68 | 5.58 | 5.58 | 7.36 | 9.85 | 20.52 | 37.97 | mTerf3 |
| Angca_08546 | 6.83 | 21.68 | 13.73 | 9.42 | 34.75 | 24.81 | 20.28 | 23.74 | pen-2 |
| Angca_11420 | 34.85 | 44.39 | 67.06 | 40.74 | 37.79 | 34.28 | 20.26 | 0.86 | lon-8 |
| Angca_12442 | 5.07 | 1.69 | 4.95 | 4.94 | 4.9 | 9.41 | 20.11 | 35.29 | BAP18 |
| Angca_08334 | 9.23 | 7.22 | 7.93 | 11.25 | 11.18 | 12.7 | 20.08 | 38.49 | Tmem165 |
| Angca_05189 | 24.9 | 32.32 | 28.18 | 22.83 | 14.15 | 19.53 | 19.95 | 6.55 | SPCC757.05c |
| Angca_01074 | 13.25 | 12.5 | 14.32 | 15.69 | 17.06 | 16.21 | 19.9 | 30.88 | feh-1 |
| Angca_12401 | 8.92 | 7.43 | 10.25 | 11.47 | 17.41 | 13.26 | 19.84 | 22.78 | ZK632.2 |
| Angca_01190 | 26.42 | 25.64 | 23.83 | 27.5 | 25.66 | 26.87 | 19.8 | 13.27 | ttyh-1 |
| Angca_09685 | 8.78 | 8.28 | 10.4 | 10.57 | 12.94 | 12.48 | 19.71 | 18.85 | 63 |
| Angca_12842 | 12.99 | 16.27 | 14.19 | 13.37 | 12.53 | 16.57 | 19.61 | 17.31 | PNPLA4 |
| Angca_08110 | 10.95 | 9.18 | 11.39 | 12.95 | 15.77 | 14.36 | 19.6 | 33.61 | mig-5 |
| Angca_12474 | 19.62 | 22.03 | 23.55 | 19.56 | 16.11 | 14.28 | 19.58 | 8.61 | prmt-5 |
| Angca_07499 | 39.42 | 36.27 | 39.38 | 36.66 | 35.95 | 22.52 | 19.5 | 3.11 | sestd1 |
| Angca_06547 | 9.22 | 9.64 | 12.88 | 11.81 | 12.55 | 15.91 | 19.48 | 22.1 | tim-1 |
| Angca_03957 | 9.96 | 17.39 | 22.66 | 26.29 | 15.04 | 30.99 | 19.47 | 47.22 | C28H8.8 |
| Angca_04475 | 11.51 | 9.78 | 11.41 | 12.7 | 14.07 | 16.42 | 19.42 | 20.39 | Ptpn23 |
| Angca_13155 | 10.35 | 9.5 | 11.11 | 10.47 | 10.4 | 15.45 | 19.2 | 28.19 | mboa-7 |
| Angca_07459 | 11.14 | 11.48 | 11.04 | 11.86 | 15.16 | 12.72 | 18.67 | 27.89 | Snx1 |
| Angca_12281 | 12.82 | 12.4 | 12.65 | 13.69 | 14.75 | 13.02 | 18.4 | 29.07 | Stub1 |
| Angca_02667 | 35.21 | 30.96 | 27.19 | 36.09 | 25.1 | 29.66 | 18.06 | 3.9 | inx-12 |
| Angca_08437 | 10.87 | 10.11 | 10.82 | 10.18 | 11.26 | 17.06 | 17.91 | 23.3 | zmp-2 |
| Angca_06529 | 8.02 | 6.69 | 9.09 | 5.42 | 18.23 | 9.98 | 17.85 | 62.7 | HSP20 |
| Angca_01323 | 13.98 | 14.53 | 14.28 | 13.36 | 15.87 | 17.48 | 17.59 | 18.46 | vps-53 |
| Angca_07518 | 11.58 | 11.65 | 11.7 | 10.33 | 11.01 | 12.79 | 17.58 | 20.21 | PDP1 |
| Angca_13440 | 9.27 | 7.85 | 11.21 | 12.22 | 16.67 | 13.68 | 17.53 | 18.53 | Huwe1 |
| Angca_08476 | 8.5 | 9.52 | 8.04 | 6.22 | 5.24 | 12.28 | 17.23 | 28.26 | pdxk-1 |
| Angca_10758 | 14.33 | 13.27 | 11.74 | 13.62 | 16.63 | 13.3 | 17.23 | 20.05 | gex-2 |
| Angca_07055 | 30.48 | 33.26 | 31.72 | 28.82 | 25.44 | 26.37 | 17.18 | 6.34 | fut-8 |
| Angca_00394 | 10.72 | 12.31 | 10.59 | 11.71 | 13.32 | 14.87 | 17.1 | 31.37 | GPR108 |
| Angca_11802 | 14.1 | 11.17 | 11.83 | 14.24 | 11.3 | 11.57 | 16.76 | 26.28 | nstp-10 |
| Angca_08647 | 14.09 | 14.55 | 13.39 | 14 | 12.38 | 12.33 | 16.68 | 8.44 | mars-1 |
| Angca_11629 | 14.32 | 13.59 | 12.28 | 15.47 | 16.6 | 12.18 | 16.66 | 25.03 | Zdhhc24 |
| Angca_03575 | 0 | 6.88 | 0 | 0 | 5.51 | 14.3 | 16.65 | 16.82 | nito |
| Angca_04423 | 13.18 | 8.7 | 12.11 | 13.1 | 13.83 | 10.4 | 16.64 | 36.51 | pgap3 |
| Angca_12811 | 13.99 | 27.93 | 27.76 | 16.92 | 15.93 | 21.87 | 16.51 | 0 | SETMAR |
| Angca_09944 | 10.78 | 9.49 | 10.32 | 11.02 | 10.77 | 13.14 | 16.36 | 21.35 | Rnf40 |
| Angca_07727 | 15.7 | 20.09 | 20.02 | 19.33 | 16.55 | 13.06 | 16.32 | 3.68 | RHM2 |
| Angca_05791 | 11.46 | 11.08 | 13.22 | 14.34 | 13.97 | 11.5 | 16.07 | 18.27 | set-2 |
| Angca_08739 | 16.86 | 15.65 | 12.01 | 14.92 | 14.75 | 13.9 | 15.89 | 4.08 | RGS7 |
| Angca_01261 | 10.31 | 8.51 | 8.95 | 10.24 | 10.78 | 14.33 | 15.75 | 17.8 | ilrun |
| Angca_08323 | 14.84 | 15.04 | 13.67 | 16.09 | 14.42 | 15.44 | 15.68 | 7.28 | 4CLL5 |
| Angca_02777 | 25.34 | 25.48 | 23.48 | 22.46 | 16.13 | 14.56 | 15.62 | 1.98 | F23F12.3/F23F12.1 |
| Angca_03589 | 12.32 | 10.15 | 12.43 | 15.59 | 14.44 | 11.84 | 15.6 | 28.56 | REXO1 |
| Angca_10275 | 40.53 | 57.51 | 51.82 | 27.77 | 16.34 | 27.02 | 15.53 | 3.96 | F13G3.3 |
| Angca_03518 | 12.43 | 22.17 | 24.22 | 15.02 | 7.71 | 13.25 | 15.5 | 8.93 | Phgdh |
| Angca_07182 | 14.04 | 11.17 | 10.29 | 10.46 | 15.5 | 14.3 | 15.42 | 34.68 | YRDC |
| Angca_13326 | 107.6 | 97.77 | 79.45 | 102.27 | 66.84 | 28.88 | 15.41 | 1.46 | ASP |
| Angca_05808 | 7.73 | 7.27 | 8.17 | 6.86 | 9.94 | 10.09 | 15.41 | 19.34 | gpdh-2 |
| Angca_01670 | 11.55 | 9.16 | 9.81 | 12.28 | 14.16 | 13.15 | 15.36 | 24.26 | cbp-1 |
| Angca_01445 | 6.67 | 6 | 8.21 | 9.54 | 13.51 | 10.42 | 15.23 | 32.65 | usp-48 |
| Angca_09258 | 8.56 | 8.03 | 9.97 | 10.25 | 12.36 | 10.69 | 15.19 | 26.34 | mask-1 |
| Angca_05295 | 15.09 | 14.25 | 14.52 | 17.31 | 15.11 | 14.41 | 15.14 | 11.21 | dpy-22 |
| Angca_02817 | 29.69 | 35.57 | 28.43 | 28.88 | 28.9 | 17.3 | 15.13 | 7.42 | Aasdhppt |
| Angca_03318 | 15.12 | 15.87 | 15.68 | 16.39 | 16.1 | 12.78 | 15.06 | 36.23 | slc20a1b |
| Angca_09775 | 11.77 | 9.57 | 11.12 | 16.83 | 15.48 | 15.96 | 15.01 | 25.76 | Pip4k2a |
| Angca_03054 | 13.67 | 11.5 | 16.65 | 17.56 | 17.52 | 14.1 | 15 | 31.82 | TFAP2A |
| Angca_05856 | 22.15 | 18.86 | 27.29 | 23.57 | 15.16 | 8.71 | 14.97 | 2.71 | hmr-1 |
| Angca_04916 | 33.24 | 20.57 | 22.97 | 25.34 | 23.8 | 22.1 | 14.84 | 2.11 | aff-1 |
| Angca_04155 | 10.21 | 7.8 | 10.44 | 11.55 | 14.38 | 11.64 | 14.79 | 21.35 | BAZ1A |
| Angca_13437 | 9.21 | 14.89 | 14.3 | 16.81 | 13.62 | 12.52 | 14.75 | 5.41 | ZK892.4 |
| Angca_03985 | 15.74 | 13.86 | 17.37 | 16.73 | 18.2 | 12.78 | 14.7 | 8.2 | Dlg5 |
| Angca_11415 | 16.21 | 26.39 | 23.34 | 25.54 | 16.63 | 14.18 | 14.62 | 8.84 | - |
| Angca_05195 | 6.75 | 8.24 | 11.92 | 8.14 | 8.51 | 13.09 | 14.43 | 24.02 | GART |
| Angca_01654 | 11.46 | 9.56 | 9.64 | 9.58 | 10.75 | 10.81 | 14.37 | 24.32 | F55H2.5 |
| Angca_01473 | 13.15 | 9.54 | 10.23 | 12.46 | 12.7 | 16.48 | 14.3 | 14.26 | mab-31 |
| Angca_12553 | 48.85 | 51.78 | 48.66 | 48.71 | 44.44 | 25.02 | 14.21 | 2.13 | aqp-10 |
| Angca_04174 | 57.24 | 80.33 | 70.82 | 55.61 | 36.21 | 47.23 | 14.19 | 0 | ncs-1 |
| Angca_12743 | 7.86 | 7.4 | 6.83 | 5.19 | 6.06 | 8.63 | 14.16 | 21.61 | vha-7 |
| Angca_07992 | 8.68 | 3.15 | 10.86 | 18.22 | 7.02 | 9.7 | 14.14 | 19.42 | ASPSCR1 |
| Angca_02404 | 8.5 | 7.33 | 7.76 | 8.93 | 11.73 | 11.11 | 14.08 | 25.01 | hlh-11 |
| Angca_04675 | 19.62 | 18.67 | 23.82 | 19.26 | 24.86 | 16.55 | 14.02 | 3.7 | KCNT2 |
| Angca_11899 | 0.53 | 0.47 | 0.36 | 0.53 | 0.42 | 2 | 14.01 | 66.57 | ds |
| Angca_06046 | 19.22 | 22.82 | 20.86 | 18.48 | 10.8 | 16.43 | 14 | 11.2 | sma-6 |
| Angca_07286 | 6.74 | 6.75 | 7.96 | 8 | 9 | 10.76 | 13.95 | 16.86 | SLX4 |
| Angca_04036 | 8.01 | 4.84 | 6.83 | 11.4 | 16.77 | 8.52 | 13.9 | 46.73 | oys |
| Angca_05863 | 11.21 | 10.56 | 9.37 | 9.88 | 14.12 | 11.41 | 13.8 | 15.97 | Lysmd3 |
| Angca_05984 | 7.92 | 5.9 | 5.37 | 6.14 | 5.08 | 4.69 | 13.68 | 80.83 | HMCN1 |
| Angca_08644 | 7.78 | 7.35 | 8.57 | 9.58 | 11.28 | 9.88 | 13.59 | 15.53 | Art7 |
| Angca_01394 | 9.68 | 7.4 | 8.56 | 9.06 | 9.39 | 11.3 | 13.37 | 14.01 | PPP2R5D |
| Angca_06770 | 2.4 | 0.61 | 2.28 | 2.35 | 23.97 | 3.79 | 13.33 | 34.44 | Eprs1 |
| Angca_07834 | 6.85 | 5.38 | 6.96 | 7.6 | 8.5 | 8.4 | 13.22 | 18.88 | ZFX |
| Angca_10583 | 0.39 | 0.62 | 1.44 | 1.32 | 0.59 | 9.24 | 13.1 | 12.87 | fgt-1 |
| Angca_12910 | 9.63 | 9.54 | 9.65 | 10.13 | 9.44 | 10.44 | 13.08 | 17.86 | chmp4b |
| Angca_02729 | 7.08 | 5.74 | 7.98 | 8.57 | 11.13 | 9.53 | 13.03 | 13.99 | dcr-1 |
| Angca_00752 | 13.14 | 16.15 | 14.57 | 13.39 | 15.64 | 12.04 | 12.96 | 7.42 | coq-2 |
| Angca_00516 | 4.61 | 4.48 | 6.12 | 5.65 | 5.05 | 8.3 | 12.9 | 12.72 | PIGW |
| Angca_00414 | 28.95 | 39.61 | 29.26 | 17.48 | 12.71 | 21.46 | 12.9 | 2.9 | SLC9A5 |
| Angca_11023 | 5.27 | 3.8 | 7.67 | 7.26 | 10.23 | 10.66 | 12.82 | 28.72 | Start1 |
| Angca_13416 | 14.65 | 18.96 | 16.07 | 8.79 | 23.24 | 15.94 | 12.58 | 5.14 | - |
| Angca_09155 | 8.26 | 8.08 | 8.17 | 8.35 | 7.79 | 9.07 | 12.58 | 21.17 | imp-2 |
| Angca_11247 | 12.63 | 12.47 | 11.94 | 12.84 | 14.33 | 12.81 | 12.24 | 5.68 | SRGAP1 |
| Angca_13330 | 2.17 | 1.74 | 4.85 | 8.52 | 11.2 | 6.55 | 12.19 | 19.76 | IFT74 |
| Angca_02950 | 10.67 | 9.26 | 9.62 | 9.99 | 8.99 | 8.44 | 12.14 | 18.18 | Dgkq |
| Angca_05263 | 38.84 | 37.18 | 29.76 | 34.06 | 36.28 | 20.1 | 11.98 | 7.92 | hex-1 |
| Angca_10759 | 12.72 | 14.28 | 12.48 | 11.76 | 13.94 | 12.3 | 11.89 | 7.81 | kti12 |
| Angca_10972 | 3.52 | 3.47 | 4.86 | 5.35 | 5.75 | 8.95 | 11.83 | 11.49 | cul-1 |
| Angca_11257 | 8.41 | 6.83 | 6.37 | 7.84 | 7.32 | 7.91 | 11.76 | 10.26 | MARCHF1 |
| Angca_04783 | 10.95 | 11.53 | 9.96 | 9.51 | 11.47 | 10.54 | 11.72 | 7.37 | trm-1 |
| Angca_05382 | 7.84 | 8.01 | 8.24 | 9 | 9.21 | 9.9 | 11.69 | 17.02 | cand-1 |
| Angca_11725 | 7.95 | 7.62 | 7.5 | 8.89 | 7.75 | 9.53 | 11.66 | 11.88 | ppfr-1 |
| Angca_03673 | 8.62 | 6.08 | 7.67 | 8.59 | 6.88 | 8.39 | 11.66 | 12.3 | chd-3 |
| Angca_07074 | 13.68 | 13.8 | 14.72 | 14.66 | 13.77 | 8.54 | 11.63 | 3.03 | P65-C |
| Angca_09929 | 6.32 | 6.86 | 7.48 | 7.63 | 7.89 | 10.57 | 11.55 | 21.27 | TRIM24 |
| Angca_05352 | 6.27 | 5.67 | 7.9 | 8.12 | 9.71 | 8.99 | 11.52 | 15.8 | let-711 |
| Angca_09806 | 2.29 | 1.92 | 2.13 | 3.13 | 2.42 | 4.19 | 11.49 | 46.77 | tbc-8 |
| Angca_09492 | 10 | 10.93 | 10.6 | 14.66 | 5.48 | 10.49 | 11.36 | 4.93 | NCAN |
| Angca_09215 | 16.7 | 15.06 | 13.99 | 15.4 | 16.81 | 13.68 | 11.32 | 6.37 | ada |
| Angca_09501 | 9.47 | 9.88 | 8.96 | 9.82 | 9.06 | 9.42 | 11.29 | 11.99 | Naus |
| Angca_02075 | 11.4 | 9.64 | 14.17 | 13.87 | 11.96 | 7.51 | 11.28 | 9.2 | RSPRY1 |
| Angca_11306 | 11.34 | 10.33 | 9.78 | 9.64 | 11.35 | 9.69 | 11.23 | 13.5 | Polr1c |
| Angca_09771 | 14.69 | 10.1 | 10.04 | 10.3 | 9.68 | 12.18 | 11.11 | 7.56 | F22B7.9 |
| Angca_06439 | 8.71 | 8 | 6.69 | 8.54 | 8.98 | 7.6 | 11.09 | 13.05 | rpoa-2 |
| Angca_11192 | 12.35 | 8.94 | 4.78 | 7.98 | 7.78 | 5.58 | 11.01 | 54.61 | bcat-1 |
| Angca_06761 | 6.65 | 4.96 | 7.12 | 7.38 | 7.95 | 9.03 | 10.98 | 13.49 | ANKFY1 |
| Angca_07713 | 9.52 | 8.1 | 8.92 | 11 | 9.86 | 9.17 | 10.91 | 14.17 | TIAL1 |
| Angca_06713 | 9.53 | 10.24 | 13.59 | 12.2 | 13.32 | 9.03 | 10.82 | 6.15 | smp-1 |
| Angca_07564 | 18.11 | 14.42 | 14.82 | 13.73 | 20.76 | 12.54 | 10.71 | 1.84 | tra-3 |
| Angca_12889 | 8.07 | 10.6 | 10.44 | 9.1 | 7.76 | 7.32 | 10.71 | 46.57 | cyd-1 |
| Angca_06411 | 13.83 | 18.63 | 14.12 | 11.4 | 12.02 | 10.68 | 10.66 | 3.71 | UPRT |
| Angca_07373 | 10.2 | 9.88 | 8.76 | 9.14 | 10.43 | 8.96 | 10.63 | 14.2 | rab-11.1 |
| Angca_04018 | 5.7 | 4.55 | 5.05 | 6.57 | 8.49 | 8.03 | 10.6 | 14.26 | drp-1 |
| Angca_10991 | 15.52 | 13.99 | 14.43 | 13.35 | 11.36 | 5.98 | 10.57 | 28.26 | baz-2 |
| Angca_02542 | 8.68 | 6.8 | 7.53 | 7.44 | 9.03 | 9.7 | 10.53 | 22.18 | abl-1 |
| Angca_12998 | 4.39 | 3.82 | 5.31 | 5.64 | 7.97 | 6.15 | 10.32 | 26.06 | let-19 |
| Angca_01417 | 6.73 | 4.35 | 6.72 | 7.88 | 8.27 | 7.61 | 10.25 | 14.49 | pry-1 |
| Angca_05443 | 7 | 4.05 | 6.44 | 9.04 | 13.85 | 7.65 | 10.23 | 23.85 | daf-2 |
| Angca_05404 | 3.62 | 1.89 | 4.16 | 5.84 | 11.47 | 6.6 | 10.21 | 32.61 | ATG9A |
| Angca_09178 | 7.35 | 7.78 | 5.99 | 6.53 | 12.09 | 8.12 | 10.16 | 15.72 | elt-6 |
| Angca_03181 | 4.49 | 2.92 | 5.37 | 6.01 | 10.62 | 7.47 | 10.16 | 18.26 | pola1 |
| Angca_10299 | 4.05 | 4.35 | 5.83 | 5.12 | 2.9 | 8.66 | 10.16 | 8.02 | abhd-3.2 |
| Angca_13046 | 8.48 | 8.23 | 7.88 | 8.16 | 8.18 | 7.17 | 10.07 | 9.31 | W09H1.5 |
| Angca_12991 | 4.19 | 0.64 | 2.88 | 5.01 | 7.38 | 6.45 | 10.03 | 15.69 | LIG3 |
| Angca_05253 | 4.57 | 5.06 | 4.59 | 5.92 | 7.45 | 4.43 | 10.01 | 39.2 | ELMOD2 |
| Angca_07830 | 6.27 | 6.5 | 7.42 | 6.97 | 9.14 | 7.29 | 10 | 13.21 | NSMCE4A |
| Angca_06054 | 8.53 | 7.36 | 6.8 | 7.61 | 9.93 | 7.24 | 9.95 | 11.14 | CG8475 |
| Angca_10965 | 4.21 | 3.07 | 3.23 | 3.94 | 6.13 | 7.21 | 9.94 | 13.82 | F54C8.6 |
| Angca_08154 | 6.9 | 6.52 | 5.83 | 7.1 | 7.74 | 7.52 | 9.93 | 14.34 | EEIG2 |
| Angca_11953 | 7.01 | 6.75 | 5.96 | 6.75 | 7.53 | 6.69 | 9.92 | 11.61 | W09C3.4 |
| Angca_12402 | 7.64 | 5.2 | 6.49 | 7.32 | 10.08 | 7.25 | 9.89 | 21.97 | usp-46 |
| Angca_12077 | 3.71 | 2.16 | 4.74 | 6.5 | 4.96 | 6.85 | 9.87 | 10.02 | N4bp2l1 |
| Angca_13147 | 5.8 | 4.47 | 4.1 | 4.07 | 3.98 | 2.53 | 9.8 | 18.35 | Nudt6 |
| Angca_09665 | 5.95 | 5.58 | 6.06 | 6.04 | 6.45 | 6.64 | 9.78 | 16.56 | CLCN3 |
| Angca_02698 | 17.13 | 8.26 | 9.78 | 14.4 | 12.83 | 9.33 | 9.73 | 1.16 | pat-9 |
| Angca_03270 | 5.41 | 5.88 | 4.6 | 4.17 | 5.42 | 5.95 | 9.73 | 27.06 | FASN |
| Angca_11461 | 2.29 | 2.9 | 5.73 | 4.84 | 3.36 | 5.08 | 9.64 | 22.51 | gar-3 |
| Angca_11681 | 16.3 | 14.63 | 9.54 | 11.61 | 10.76 | 13.24 | 9.59 | 2.85 | NCEH1 |
| Angca_02843 | 7.27 | 8.65 | 7.58 | 7.13 | 8.8 | 5.56 | 9.59 | 9.46 | nmad-1 |
| Angca_02889 | 3.2 | 1.55 | 2.79 | 4.22 | 4.04 | 5.35 | 9.46 | 15.43 | NELFA |
| Angca_01295 | 5.26 | 4.85 | 5 | 4.64 | 6.3 | 5.56 | 9.46 | 8.9 | sta-1 |
| Angca_10378 | 5.15 | 3.75 | 5.91 | 6.49 | 6.77 | 7.66 | 9.39 | 13.31 | Arfgap1 |
| Angca_10826 | 24.56 | 20.74 | 19.33 | 22.81 | 17.75 | 12.64 | 9.3 | 5.14 | myrf-1 |
| Angca_03734 | 1.95 | 0.93 | 2.61 | 3.91 | 5.63 | 4.43 | 9.29 | 24.48 | INO80E |
| Angca_09457 | 10.68 | 11.87 | 9.73 | 8.4 | 11.25 | 8.54 | 9.27 | 6.42 | Oplah |
| Angca_09273 | 30.6 | 27.89 | 24.27 | 27.97 | 23.23 | 13.6 | 9.21 | 2.48 | FKBP9 |
| Angca_04156 | 8.18 | 6.78 | 7.39 | 7.89 | 9.07 | 8.51 | 9.15 | 13.53 | Ralgapb |
| Angca_07199 | 8.57 | 8.37 | 7.08 | 4.81 | 5.99 | 3.98 | 9.14 | 12.69 | mml-1 |
| Angca_12502 | 3.01 | 1.71 | 3.86 | 3.44 | 7.88 | 4.95 | 9.09 | 19.24 | hira-1 |
| Angca_00537 | 10.59 | 7.58 | 5.74 | 7.7 | 5.22 | 5.99 | 9.08 | 26.32 | unc-103 |
| Angca_08495 | 6.42 | 6.05 | 6.48 | 7.01 | 8.13 | 7.55 | 9.08 | 9.99 | csnk-1 |
| Angca_11763 | 9.07 | 0.38 | 0 | 2.08 | 3.55 | 0.16 | 9.05 | 16.25 | mep-1 |
| Angca_02767 | 6.14 | 6.19 | 6.37 | 6.75 | 8.79 | 6.55 | 9.01 | 10.03 | sqv-3 |
| Angca_08449 | 6.4 | 5.86 | 6.46 | 7.47 | 8.28 | 7.1 | 8.8 | 8.89 | cka-2 |
| Angca_05406 | 0.92 | 0.28 | 3.26 | 3.04 | 4.26 | 5.01 | 8.77 | 22.2 | ani-2 |
| Angca_03142 | 5.86 | 5 | 5.76 | 6.16 | 6.34 | 7.93 | 8.72 | 10.23 | Znf598 |
| Angca_01157 | 15.09 | 15.02 | 13.93 | 11.08 | 6.87 | 9.63 | 8.67 | 1.04 | Ork1 |
| Angca_12881 | 7.28 | 1.98 | 1.89 | 10.99 | 4.59 | 12.4 | 8.63 | 47.41 | zyg-1 |
| Angca_00469 | 15.96 | 14.94 | 19.63 | 18.08 | 15.1 | 11.3 | 8.56 | 5.55 | nhr-22 |
| Angca_10777 | 8.96 | 14.95 | 43.26 | 28.93 | 30.62 | 16.18 | 8.42 | 0 | FAXDC2 |
| Angca_01253 | 8.59 | 9.68 | 11.32 | 9.08 | 11.97 | 10.53 | 8.38 | 3.82 | SPR |
| Angca_04368 | 7.28 | 6.84 | 7.61 | 7.39 | 7.51 | 7.26 | 8.24 | 3.91 | riok-2 |
| Angca_09129 | 4.87 | 4.22 | 4.88 | 5.16 | 4.41 | 6.24 | 8.17 | 21.44 | clp-1 |
| Angca_08454 | 6.88 | 7.86 | 8.12 | 7.93 | 7.59 | 7.11 | 8.14 | 15.54 | daf-25 |
| Angca_09287 | 9.08 | 7.86 | 8.91 | 7.72 | 7.79 | 8.6 | 8.14 | 4.79 | LACTB |
| Angca_08809 | 0.49 | 0.33 | 2.16 | 1.55 | 4.6 | 5.04 | 8.05 | 10.93 | msh-5 |
| Angca_00194 | 8.9 | 7.56 | 6.76 | 6.49 | 7.85 | 8.45 | 8.03 | 2.97 | Fdx1 |
| Angca_07578 | 6.11 | 11.06 | 9.75 | 8.22 | 9.32 | 7.08 | 8.01 | 3.91 | RAB32 |
| Angca_01909 | 6.13 | 7.17 | 6.97 | 7.34 | 6.37 | 8.44 | 7.95 | 11.87 | sqv-7 |
| Angca_02910 | 7.61 | 7.92 | 17.12 | 12.04 | 9.16 | 8.19 | 7.91 | 2.2 | hbl-1 |
| Angca_02131 | 5.27 | 3.82 | 4.66 | 5.45 | 5.82 | 5.3 | 7.67 | 9.22 | taf-1 |
| Angca_04677 | 7.62 | 7.01 | 5.02 | 8.42 | 6.82 | 6.29 | 7.64 | 9.21 | TCAIM |
| Angca_04770 | 5.87 | 11.66 | 11.36 | 9.26 | 5.8 | 7.94 | 7.57 | 2.21 | chat-1 |
| Angca_08536 | 7.2 | 7.07 | 6.83 | 6.31 | 6.31 | 6.42 | 7.49 | 3.31 | grh-1 |
| Angca_03188 | 10.58 | 6.95 | 6.83 | 7.66 | 8.84 | 7.28 | 7.47 | 4.2 | chp-1 |
| Angca_13180 | 3.14 | 4.7 | 10.4 | 11 | 5.07 | 8.14 | 7.36 | 14.46 | asic1a |
| Angca_01340 | 2.52 | 2.18 | 3.02 | 2.72 | 3.43 | 4.21 | 7.35 | 19 | F56D1.1 |
| Angca_10001 | 6.51 | 6.83 | 6.69 | 6.11 | 6.73 | 6.71 | 7.32 | 3.27 | B3GLCT |
| Angca_01148 | 5.34 | 5.16 | 5.3 | 6.25 | 7.33 | 5.68 | 7.32 | 11.5 | SLC25A22 |
| Angca_01625 | 3.58 | 2.97 | 4.66 | 4.83 | 5.81 | 4.5 | 7.3 | 8.15 | lrk-1 |
| Angca_13300 | 2.23 | 1.71 | 2.31 | 2.22 | 3.78 | 2.25 | 7.2 | 10.53 | RBE1 |
| Angca_09921 | 4.18 | 4.03 | 4.34 | 4.63 | 4.61 | 5.55 | 7.04 | 7.46 | thoc5 |
| Angca_01997 | 5.85 | 6.31 | 5.72 | 9.66 | 6.12 | 16.58 | 7.03 | 20.77 | foxi1c |
| Angca_04353 | 2.38 | 2.23 | 2.28 | 4.4 | 4.85 | 3.83 | 6.95 | 26.4 | mat-1 |
| Angca_11177 | 6.83 | 5.42 | 4.56 | 4.42 | 4.28 | 5.06 | 6.9 | 12.03 | sorf-1 |
| Angca_00335 | 2.67 | 3.32 | 5.49 | 6.75 | 5.79 | 3.29 | 6.88 | 2.11 | tshr |
| Angca_00209 | 1.03 | 0.96 | 0.84 | 1.93 | 3.59 | 2.61 | 6.73 | 14.8 | tyr-3 |
| Angca_02398 | 5.47 | 4.71 | 4.74 | 5.48 | 6.57 | 6.27 | 6.73 | 8.68 | zyg-8 |
| Angca_13286 | 5.49 | 2.54 | 3.88 | 6.31 | 8.7 | 6.2 | 6.73 | 18.64 | hda-4 |
| Angca_11474 | 2.2 | 1.48 | 2.35 | 2.6 | 3.69 | 3.42 | 6.72 | 10.15 | sid-3 |
| Angca_12330 | 0.64 | 0.54 | 0.72 | 0.56 | 0.49 | 2.06 | 6.67 | 7.79 | nep-1 |
| Angca_00638 | 2.81 | 3.12 | 3.95 | 4.17 | 3.83 | 4.42 | 6.59 | 5.88 | hay |
| Angca_06461 | 4.93 | 4.47 | 4.39 | 4.96 | 5.77 | 4.98 | 6.5 | 9.97 | trr |
| Angca_09795 | 4.19 | 3.88 | 4.64 | 4.8 | 5.76 | 4.96 | 6.47 | 7.4 | ACVR2A |
| Angca_05198 | 4.79 | 4.18 | 4.6 | 4.53 | 6.1 | 4.51 | 6.4 | 6.81 | lin-45 |
| Angca_03667 | 5.26 | 3.91 | 4.82 | 6.27 | 7.06 | 7.74 | 6.37 | 9.49 | SPBC3D6.12 |
| Angca_05226 | 2.71 | 2.84 | 3.5 | 3.3 | 3.88 | 4.56 | 6.3 | 6.38 | lag-1 |
| Angca_13261 | 4.96 | 4.06 | 4.62 | 4.78 | 7 | 6.46 | 6.27 | 7.03 | Htt |
| Angca_00133 | 7.06 | 5.89 | 7.38 | 9.06 | 6.08 | 5.05 | 6.22 | 3.74 | F54D1.6 |
| Angca_10888 | 10.16 | 6.64 | 10.87 | 12.55 | 9.55 | 10.03 | 6.09 | 1.84 | T07F12.1 |
| Angca_02643 | 10.78 | 10.92 | 9.9 | 8.34 | 5.06 | 6.8 | 6.06 | 0.39 | At4g01020 |
| Angca_02850 | 0.21 | 0.35 | 0.37 | 0.55 | 0.89 | 2.16 | 6.05 | 6.65 | R10E12.2 |
| Angca_03085 | 2.27 | 1.54 | 2.73 | 3.4 | 4.44 | 3.54 | 6 | 7.72 | WDR36 |
| Angca_04502 | 4.62 | 4.15 | 4.43 | 5.06 | 6.72 | 4.73 | 6 | 10.34 | gapvd1 |
| Angca_02990 | 7.41 | 7.28 | 8.27 | 9.25 | 5.87 | 7.69 | 5.97 | 5.9 | cdk-8 |
| Angca_02559 | 13.25 | 13.81 | 12.22 | 15.4 | 13.88 | 7.97 | 5.97 | 2.55 | psuG |
| Angca_12380 | 4.3 | 2.5 | 5.08 | 5.46 | 4.89 | 4.05 | 5.96 | 11.44 | Trio |
| Angca_02538 | 4.54 | 3.51 | 4.2 | 5.11 | 4.77 | 5.27 | 5.72 | 9.15 | cdc-14 |
| Angca_09949 | 1.2 | 1.12 | 2.57 | 1.74 | 2.24 | 3.84 | 5.6 | 6.51 | snpc-4 |
| Angca_04997 | 5.45 | 5 | 5.01 | 5.2 | 4.77 | 5.07 | 5.6 | 5.41 | cap-2 |
| Angca_01799 | 1.97 | 1.82 | 3.1 | 2.93 | 3.79 | 3.77 | 5.6 | 12.41 | RASA1 |
| Angca_03530 | 1.29 | 1.26 | 0.86 | 1.36 | 0.54 | 2.1 | 5.59 | 4.06 | Slc2a7 |
| Angca_02701 | 2.31 | 1.9 | 2.83 | 3.25 | 4.09 | 3.12 | 5.57 | 7.71 | NPRL2 |
| Angca_10944 | 3.44 | 2.15 | 2.85 | 2.24 | 1.53 | 1.68 | 5.57 | 3.97 | Ppn |
| Angca_12161 | 3.73 | 2.85 | 2.23 | 4.98 | 4.53 | 5.2 | 5.56 | 6.28 | nhr-47 |
| Angca_09271 | 5.93 | 6.26 | 10 | 9.07 | 5.48 | 7.1 | 5.48 | 1.33 | CHRND |
| Angca_11610 | 9.05 | 13.03 | 10.24 | 8.82 | 3.08 | 7.72 | 5.45 | 1.04 | Chrnd |
| Angca_07795 | 3.39 | 4.6 | 4.64 | 4.04 | 4.39 | 3.94 | 5.33 | 14.31 | Gba2 |
| Angca_04084 | 0.01 | 0.01 | 0.11 | 0.07 | 0.16 | 1.06 | 5.33 | 31.36 | SOD |
| Angca_10412 | 6.56 | 6.02 | 4.93 | 6.93 | 6.54 | 7.45 | 5.25 | 1.08 | prk-2 |
| Angca_00019 | 4.36 | 3.33 | 4.2 | 5.05 | 4.63 | 4.26 | 5.13 | 7.14 | tax-6 |
| Angca_08135 | 2.2 | 1.1 | 1.6 | 3.07 | 4.6 | 3.22 | 5.04 | 11.9 | tbc1d23 |
| Angca_10756 | 8.29 | 4.86 | 13.05 | 7.72 | 2.5 | 4.53 | 5.03 | 0.23 | onecut |
| Angca_00263 | 0.09 | 0.09 | 1 | 0.52 | 2.37 | 2.14 | 5.03 | 7.61 | wee-1.3 |
| Angca_04335 | 1.23 | 0.73 | 0.93 | 1.35 | 3.71 | 1.66 | 5.01 | 32.7 | cdh-4 |
| Angca_11000 | 1.22 | 0.58 | 1.41 | 2.17 | 6.92 | 2.65 | 5.01 | 11.8 | smc-3 |
| Angca_12070 | 4.22 | 3.72 | 4.14 | 4.2 | 5.06 | 4.63 | 4.97 | 5.34 | POLDIP2 |
| Angca_00200 | 1.98 | 2.98 | 2.48 | 3.02 | 4.38 | 4.1 | 4.9 | 8.5 | TGFBR1 |
| Angca_04875 | 10.55 | 10.17 | 10.02 | 5.8 | 3.18 | 6.92 | 4.88 | 2.9 | mlt-7 |
| Angca_10338 | 5.96 | 6.87 | 5.4 | 5.88 | 5.49 | 5.05 | 4.82 | 2.42 | IDH3G |
| Angca_04517 | 5.23 | 6.03 | 7 | 4.98 | 7.07 | 7.78 | 4.64 | 2.23 | Cyt-b5 |
| Angca_11143 | 8.88 | 9.47 | 8.19 | 9 | 6.02 | 5.22 | 4.61 | 1.23 | ZC84.1 |
| Angca_00966 | 3.76 | 7.31 | 7.4 | 5.5 | 6.4 | 5.33 | 4.56 | 1.69 | sphk-1 |
| Angca_10549 | 2.29 | 3.81 | 3.46 | 2.26 | 2.61 | 2.15 | 4.55 | 3.82 | dyf-7 |
| Angca_10157 | 4.03 | 3.27 | 2.73 | 3.06 | 1.83 | 3.43 | 4.52 | 11.56 | syd-1 |
| Angca_06739 | 3.07 | 2.22 | 2.91 | 2.92 | 3.15 | 3.25 | 4.48 | 9.2 | unc-37 |
| Angca_06286 | 4.75 | 3.61 | 4.14 | 4.62 | 4.03 | 3.46 | 4.42 | 3.13 | Rasl12 |
| Angca_12050 | 3.77 | 3.05 | 3 | 3.29 | 2.65 | 2.43 | 4.35 | 7.85 | gtf-2A2 |
| Angca_02510 | 0.18 | 0.07 | 0.32 | 0.24 | 0.39 | 0.85 | 4.31 | 42.79 | tdc-1 |
| Angca_10932 | 2.16 | 1.24 | 1.83 | 3.36 | 2.34 | 4.18 | 4.26 | 4.58 | C06G4.5 |
| Angca_00923 | 0.39 | 1.34 | 1.14 | 1.48 | 1.45 | 0.71 | 4.23 | 13.54 | Skp2 |
| Angca_10840 | 4.36 | 2.87 | 3.57 | 2.51 | 4.85 | 4.94 | 4.2 | 4.56 | daf-3 |
| Angca_00064 | 1.49 | 3.18 | 3.37 | 2.03 | 1.11 | 3.04 | 4.18 | 7.81 | XDH |
| Angca_13048 | 6.43 | 6.99 | 8.46 | 6.38 | 4.81 | 5.1 | 4.08 | 0.58 | nphp-1 |
| Angca_02148 | 7.21 | 8.1 | 6.19 | 9.42 | 6.55 | 4.64 | 4.07 | 0.36 | CCKAR |
| Angca_13377 | 1.31 | 1.15 | 3.85 | 2.77 | 3.86 | 3.85 | 4.04 | 13.72 | mup-4 |
| Angca_11545 | 3.82 | 4.49 | 5.76 | 4.44 | 4.15 | 4.92 | 4 | 1.56 | TTC38 |
| Angca_03353 | 4.61 | 8.1 | 7.49 | 4.52 | 3.62 | 4.07 | 3.97 | 0.47 | C33G3.4 |
| Angca_08311 | 2.95 | 1.31 | 1.59 | 2.68 | 5.68 | 5.81 | 3.96 | 6.47 | Ecpas |
| Angca_05974 | 2.81 | 2.29 | 3.01 | 3.84 | 4.51 | 5 | 3.95 | 7.79 | OSBPL8 |
| Angca_08405 | 6.28 | 6.3 | 3.99 | 9.61 | 5.36 | 4.28 | 3.89 | 1.09 | C09F5.1 |
| Angca_02079 | 1.66 | 1.87 | 2.32 | 2.46 | 2.53 | 2.84 | 3.85 | 3.92 | CBG18823 |
| Angca_02193 | 3.68 | 4.93 | 4.11 | 3.83 | 4.54 | 3.32 | 3.81 | 1.29 | snx-17 |
| Angca_03191 | 2.38 | 1.68 | 1.99 | 2.19 | 2.56 | 2.72 | 3.79 | 7.25 | lin-59 |
| Angca_00056 | 3.19 | 2.62 | 2.73 | 2.77 | 2.78 | 2.68 | 3.71 | 3.98 | - |
| Angca_07047 | 4.56 | 4.5 | 5.77 | 7.95 | 1.43 | 3.59 | 3.7 | 1.2 | rft-1 |
| Angca_13100 | 2.28 | 1.89 | 2.31 | 3.17 | 4.39 | 2.47 | 3.67 | 6.97 | ptc-1 |
| Angca_04277 | 2.1 | 1.9 | 2.61 | 2.7 | 2.48 | 2.78 | 3.65 | 4.34 | ATJ16 |
| Angca_12105 | 2.51 | 2.65 | 3.23 | 2.38 | 2.91 | 2.7 | 3.63 | 3.9 | spas-1 |
| Angca_10523 | 3.21 | 2.91 | 2.72 | 3.37 | 3.14 | 3.57 | 3.59 | 5.48 | saeg-1 |
| Angca_11837 | 2.87 | 2.92 | 3.13 | 3.29 | 3.49 | 3.13 | 3.55 | 4.65 | PEX3 |
| Angca_06007 | 4.99 | 3.34 | 3.75 | 4.36 | 3.86 | 4.29 | 3.54 | 0.95 | ABTB2 |
| Angca_01561 | 4.74 | 3.84 | 5.27 | 6.8 | 4.62 | 3.67 | 3.5 | 2.01 | MACF1 |
| Angca_11145 | 2.58 | 2.32 | 2.76 | 3.01 | 3.97 | 2.53 | 3.32 | 4.96 | sdc-2 |
| Angca_13188 | 2.22 | 1.97 | 2.13 | 2.72 | 2.6 | 2.19 | 3.3 | 3.4 | Nmd3 |
| Angca_05884 | 2.53 | 4.98 | 3.45 | 4.03 | 2.96 | 3.3 | 3.28 | 0.8 | R07B1.3 |
| Angca_00802 | 3.59 | 2.78 | 2.72 | 1.59 | 2.53 | 1.88 | 3.26 | 5.29 | pef-1 |
| Angca_11728 | 4.12 | 4.72 | 4.26 | 4.03 | 3.54 | 3.21 | 3.24 | 0.91 | PGPEP1 |
| Angca_06090 | 2.99 | 1.57 | 2.42 | 2 | 0.93 | 1.53 | 3.22 | 7.16 | twk-7 |
| Angca_03773 | 8.25 | 15.55 | 10.66 | 5.63 | 2.81 | 5.58 | 3.21 | 0.19 | sto-4 |
| Angca_02132 | 2.58 | 2.29 | 1.71 | 2.08 | 1.21 | 2.44 | 3.18 | 9.26 | atp1a1 |
| Angca_08556 | 0.9 | 0 | 1.28 | 0.87 | 0 | 0 | 3.11 | 7.33 | IP6K3 |
| Angca_08085 | 2.89 | 5.24 | 8.17 | 4.37 | 3.29 | 3.03 | 3.1 | 0.96 | ptr-18 |
| Angca_02555 | 2.72 | 1.99 | 2.47 | 2.7 | 3.11 | 2.37 | 3.1 | 4.71 | toca-1 |
| Angca_08741 | 1.32 | 1.2 | 1.57 | 2.25 | 2.63 | 2.46 | 3.09 | 5.33 | EEED8.10 |
| Angca_10362 | 8.43 | 12.29 | 11.23 | 12.87 | 12 | 3.46 | 3.03 | 1.05 | nrf-6 |
| Angca_05649 | 1.2 | 1.77 | 0.62 | 0.73 | 1.02 | 1.33 | 3.02 | 1.98 | dmsr-1 |
| Angca_00254 | 0.7 | 1.13 | 1.78 | 2.07 | 4.01 | 2.42 | 2.99 | 4.73 | faah-1 |
| Angca_02301 | 2.11 | 0.41 | 0.64 | 0.76 | 1.32 | 3.41 | 2.99 | 2.83 | hsp-110 |
| Angca_12332 | 1.63 | 0.6 | 0.83 | 1.47 | 2.23 | 1.71 | 2.93 | 6.58 | max-2 |
| Angca_08481 | 1.85 | 1.81 | 2.21 | 2.42 | 3.11 | 2.3 | 2.92 | 2.91 | spt-16 |
| Angca_02021 | 1.1 | 1.11 | 1.44 | 1.17 | 1.48 | 1.83 | 2.91 | 3.01 | ani-1 |
| Angca_00621 | 0.86 | 0.47 | 0.49 | 0.63 | 0.65 | 1.37 | 2.87 | 7.31 | phlp-2 |
| Angca_00926 | 3.17 | 2.92 | 5.31 | 5.28 | 1.86 | 1.67 | 2.78 | 0.35 | Upb1 |
| Angca_01770 | 0.64 | 0.37 | 0.52 | 0.17 | 0.52 | 0.38 | 2.71 | 9.59 | CUBN |
| Angca_04007 | 1.75 | 1.21 | 1.66 | 1.91 | 2.41 | 2.04 | 2.71 | 3.96 | dcap-2 |
| Angca_03583 | 0.5 | 1.03 | 0.74 | 0.55 | 0.18 | 2.03 | 2.65 | 8.77 | ced-3 |
| Angca_10977 | 4.18 | 4.5 | 3.81 | 3.21 | 4.29 | 3.01 | 2.61 | 0.69 | CBG09016 |
| Angca_11007 | 2.87 | 1.78 | 2.16 | 2.71 | 3.9 | 2.79 | 2.56 | 4.04 | glr-2 |
| Angca_04321 | 3.3 | 4.06 | 4.58 | 4.38 | 3.5 | 1.84 | 2.51 | 1.77 | Esyt2 |
| Angca_08128 | 4.61 | 3.33 | 3.16 | 3.28 | 3.92 | 7.03 | 2.46 | 1.22 | CG12163 |
| Angca_02279 | 0.18 | 0.13 | 0.67 | 0.49 | 0.73 | 1.75 | 2.46 | 2.31 | wago-1 |
| Angca_02597 | 2.53 | 2.93 | 2.78 | 2.43 | 2.86 | 2.58 | 2.44 | 2.37 | Fdps |
| Angca_11916 | 0.22 | 0.11 | 0.22 | 0.56 | 1.42 | 1.34 | 2.43 | 7.99 | FANCI |
| Angca_01690 | 1.64 | 1.45 | 1.87 | 1.98 | 2.45 | 2.21 | 2.43 | 3.06 | SLC30A9 |
| Angca_04501 | 3.65 | 3.12 | 4.1 | 2.67 | 4.73 | 3.13 | 2.37 | 0.73 | sto-3 |
| Angca_05287 | 4.29 | 4.17 | 1.25 | 2.48 | 3.04 | 3.36 | 2.37 | 0.22 | zig-8 |
| Angca_13192 | 1.51 | 1.27 | 1.58 | 1.62 | 1.91 | 1.9 | 2.28 | 2.92 | DIP2C |
| Angca_05206 | 2.84 | 2.52 | 2.74 | 2.86 | 2.59 | 2.67 | 2.24 | 1 | Atp8b2 |
| Angca_12086 | 4.52 | 3.58 | 4.02 | 3.96 | 0.76 | 3.06 | 2.23 | 0.54 | F28H7.8 |
| Angca_01600 | 1.83 | 1.84 | 1.88 | 1.89 | 1.65 | 2.08 | 2.22 | 2.67 | unc-40 |
| Angca_04483 | 1.93 | 1.52 | 1.72 | 1.72 | 2.55 | 1.52 | 2.18 | 8.37 | cam-1 |
| Angca_04088 | 3.31 | 3.03 | 2.04 | 3.83 | 2.34 | 3.36 | 2.17 | 0.36 | SOD |
| Angca_10147 | 1.77 | 1.23 | 1.64 | 0.87 | 0.35 | 0.97 | 2.14 | 6.26 | adal-a |
| Angca_04490 | 3.52 | 9.88 | 4.74 | 6.51 | 6.89 | 6.11 | 2.1 | 0 | Pif1 |
| Angca_12875 | 1.49 | 1.54 | 1.5 | 1.36 | 1.75 | 1.46 | 2.02 | 2 | gna-1 |
| Angca_03770 | 2.11 | 1.55 | 1.42 | 1.99 | 2.16 | 1.53 | 2.02 | 6.8 | Wdr44 |
| Angca_06636 | 2.04 | 1.73 | 2.17 | 2.2 | 2.23 | 1.69 | 2.02 | 1.07 | Gnl2 |
| Angca_06063 | 1.69 | 1.7 | 1.84 | 2.31 | 1.88 | 1.54 | 2.01 | 3.34 | unc-8 |
| Angca_01278 | 1.76 | 1.71 | 1.63 | 1.59 | 1.92 | 1.72 | 1.98 | 0.67 | MTP3 |
| Angca_05176 | 1.56 | 1.27 | 1.1 | 1.15 | 0.74 | 1.16 | 1.93 | 3.9 | dyf-4 |
| Angca_12790 | 4.14 | 5.84 | 8.96 | 1.56 | 1.79 | 3.22 | 1.93 | 0.55 | che-13 |
| Angca_05448 | 2.99 | 2.98 | 4.75 | 2.87 | 2.15 | 3.54 | 1.9 | 0.62 | acc-4 |
| Angca_04686 | 2.04 | 2.5 | 3.18 | 3.38 | 3.07 | 2.5 | 1.9 | 0.08 | TMEM164 |
| Angca_11663 | 2.32 | 3.25 | 4.83 | 2.45 | 2.3 | 1.18 | 1.87 | 0.64 | ptr-18 |
| Angca_09808 | 1.84 | 1.38 | 0.76 | 1.35 | 1.14 | 0.7 | 1.83 | 7.77 | B0361.4 |
| Angca_05348 | 6.5 | 6.76 | 5.32 | 7.24 | 4.2 | 2.96 | 1.75 | 0.29 | ggr-1 |
| Angca_02596 | 0.83 | 0.57 | 0.74 | 0.96 | 0.97 | 1.22 | 1.74 | 4.83 | Wasf3 |
| Angca_07401 | 0.77 | 0.5 | 0.72 | 0.86 | 0.9 | 1.59 | 1.72 | 1.56 | Gpaa1 |
| Angca_09934 | 0.91 | 0.84 | 1.15 | 1.24 | 1.51 | 1.17 | 1.68 | 2.2 | Ccdc47 |
| Angca_04467 | 0.65 | 0.62 | 0.87 | 1.14 | 0.87 | 1.43 | 1.68 | 1.82 | Mctp1 |
| Angca_02490 | 2.27 | 2.71 | 1.31 | 2.9 | 2.09 | 1.58 | 1.63 | 0.28 | acr-16 |
| Angca_12066 | 13.22 | 6.63 | 10.58 | 14.53 | 4.77 | 5.1 | 1.48 | 0.21 | gab-1 |
| Angca_03542 | 0.54 | 0.89 | 0.9 | 0.89 | 0.71 | 1.16 | 1.47 | 2.9 | F33H1.3 |
| Angca_11034 | 0.76 | 0.78 | 0.8 | 0.73 | 1.19 | 0.94 | 1.46 | 2.07 | polq-1 |
| Angca_08683 | 2.16 | 2.43 | 2.98 | 1.89 | 1.42 | 1.25 | 1.42 | 0.42 | nas-12 |
| Angca_13044 | 1.44 | 1.28 | 1.66 | 1.76 | 1.47 | 1.27 | 1.38 | 2.25 | gus |
| Angca_09880 | 0.73 | 0.56 | 0.77 | 1.49 | 1.53 | 1.2 | 1.37 | 3.31 | F13B12.4 |
| Angca_04026 | 1.39 | 1.25 | 1.18 | 1.18 | 1.33 | 1.26 | 1.36 | 0.73 | ric-8 |
| Angca_00672 | 3.88 | 3.97 | 4.58 | 3.05 | 2.33 | 1.09 | 1.34 | 0.78 | mgl-1 |
| Angca_09195 | 1.74 | 1.83 | 3.11 | 2.09 | 1.66 | 1.73 | 1.33 | 0.91 | best-9 |
| Angca_02117 | 1.86 | 1.39 | 1.56 | 1.76 | 1.66 | 1.79 | 1.26 | 1.21 | MTERF4 |
| Angca_09888 | 2.09 | 1.26 | 0.64 | 1.18 | 0.95 | 1.84 | 1.2 | 0.21 | mom-2 |
| Angca_09605 | 2.56 | 2.66 | 2.84 | 2.75 | 3.2 | 2.28 | 1.19 | 1.77 | F09A5.2 |
| Angca_03256 | 1.66 | 0.99 | 1.62 | 3.2 | 1.38 | 0.67 | 1.12 | 0 | F13C5.5 |
| Angca_01563 | 0.91 | 0.42 | 0.66 | 1.3 | 1.22 | 0.95 | 1.04 | 13.47 | Plec |
| Angca_05019 | 2.73 | 3.71 | 4.32 | 1.81 | 1.37 | 1.4 | 0.99 | 0.16 | T25E4.1 |
| Angca_04910 | 1.73 | 2.15 | 0.21 | 0.06 | 0.69 | 0.95 | 0.98 | 5.68 | Gpat4 |
| Angca_07794 | 0.27 | 0.19 | 0.5 | 0.56 | 0.49 | 0.56 | 0.98 | 5.16 | che-3 |
| Angca_10322 | 2.29 | 1.32 | 1.44 | 1.13 | 1.07 | 1.5 | 0.96 | 0 | ceh-6 |
| Angca_12673 | 1.42 | 1.23 | 1.97 | 1.24 | 0.85 | 1.23 | 0.94 | 0.31 | glb-9 |
| Angca_06927 | 2.12 | 1.6 | 1.44 | 2.15 | 1.9 | 1.64 | 0.9 | 0.19 | GLIPR2 |
| Angca_12869 | 1.69 | 0.95 | 1.16 | 1.76 | 1.53 | 1.37 | 0.83 | 0.24 | AMN1 |
| Angca_02760 | 1.71 | 1.87 | 2.09 | 1.41 | 1.9 | 1.44 | 0.78 | 0.2 | php-3 |
| Angca_02472 | 1.81 | 1.79 | 2.73 | 1.96 | 3.45 | 1.84 | 0.62 | 0.44 | Asator |
| Angca_03354 | 1.94 | 1.63 | 1.35 | 1.88 | 1.3 | 0.93 | 0.39 | 0.31 | lgc-50 |
| Angca_04167 | 0 | 0 | 0 | 0 | 0 | 0 | 0 | 8.35 | PSMC5 |
| Angca_05950 | 0 | 0 | 3.97 | 0 | 2.16 | 0 | 0 | 8.76 | CBG02625 |
| Angca_10555 | 39.55 | 0 | 99.21 | 0 | 0 | 0 | 0 | 0 | gpc-1 |
| Angca_06738 | 2.56 | 2.73 | 5.89 | 3.14 | 2.51 | 0 | 0 | 0 | Cyt-b5 |
| Angca_02799 | 0 | 0 | 0 | 0 | 0.73 | 0 | 0 | 64.82 | ced-7 |
| Angca_08355 | 0 | 0 | 0 | 0 | 0 | 0 | 0 | 15.27 | sumv-1 |
| Angca_06641 | 1085.88 | 1288.61 | 1065.73 | 1061.66 | 1064.39 | 1118.38 | 1181.13 | 950.57 | - |
| Angca_11942 | 800.46 | 1227.44 | 1341.24 | 1376.87 | 1274.22 | 1122.01 | 1042.76 | 117.06 | - |
| Angca_00303 | 145.43 | 66.57 | 320.09 | 620.71 | 1031.6 | 724.06 | 554.54 | 2457.87 | - |
| Angca_07906 | 350.63 | 271.42 | 307.57 | 341.18 | 295.15 | 294.22 | 398.35 | 386.12 | - |
| Angca_05818 | 351.37 | 453.17 | 352.98 | 281.97 | 248.38 | 396.53 | 324.38 | 70.91 | - |
| Angca_07287 | 129.24 | 115.41 | 123.51 | 143.76 | 175.51 | 148.03 | 204.7 | 405.33 | - |
| Angca_12880 | 0 | 0 | 0 | 0 | 82.2 | 0 | 201.07 | 177 | - |
| Angca_13298 | 169.95 | 260.6 | 264.28 | 315.09 | 214.23 | 207.68 | 186.97 | 114.26 | - |
| Angca_04337 | 304.41 | 327.81 | 293.44 | 354.15 | 333.13 | 255.47 | 169.8 | 70.55 | - |
| Angca_12058 | 85.38 | 65.71 | 70.95 | 75.02 | 111.17 | 97.89 | 131.19 | 250.8 | - |
| Angca_13031 | 256.76 | 199.54 | 249.95 | 326.74 | 247.2 | 262.75 | 128.77 | 18.79 | - |
| Angca_07858 | 103.15 | 98.84 | 100.13 | 100.83 | 135.5 | 120.3 | 128.01 | 177.58 | - |
| Angca_11598 | 94.41 | 34.23 | 76.75 | 144.78 | 135.97 | 97.3 | 113.53 | 359.89 | - |
| Angca_06630 | 357.52 | 429.59 | 413.62 | 194.65 | 91.66 | 266.19 | 100.91 | 1.07 | - |
| Angca_11791 | 1 | 3.12 | 9.42 | 1.87 | 11.57 | 39.77 | 95.44 | 339.48 | - |
| Angca_03621 | 84.81 | 79.38 | 71.91 | 87.96 | 94.92 | 65.41 | 94.37 | 128.33 | - |
| Angca_09268 | 96.89 | 120.44 | 94.13 | 66.68 | 94.46 | 83.99 | 89.81 | 49.98 | - |
| Angca_08202 | 48.29 | 42.67 | 53.65 | 52.63 | 77.07 | 58.63 | 76.76 | 81.1 | - |
| Angca_03271 | 45.35 | 48.97 | 55.65 | 52.18 | 48.43 | 61.41 | 74 | 75.21 | - |
| Angca_09908 | 97.04 | 100.42 | 108.32 | 94.04 | 96.16 | 96.56 | 70.49 | 29.84 | - |
| Angca_02974 | 0.56 | 0.15 | 14.6 | 5.29 | 10.37 | 37.86 | 68.97 | 161.44 | - |
| Angca_05147 | 0.28 | 0.62 | 2.36 | 2.23 | 2.58 | 18.3 | 65.82 | 460.48 | - |
| Angca_01587 | 26.73 | 23.07 | 32 | 33.5 | 45.96 | 44.19 | 62.74 | 76.22 | - |
| Angca_03737 | 33.92 | 26.12 | 28.97 | 28.23 | 51.51 | 50.1 | 62.62 | 81.47 | - |
| Angca_08543 | 53.65 | 56.81 | 65.11 | 71.44 | 69.26 | 59.77 | 58.93 | 25.92 | - |
| Angca_00075 | 195.01 | 197.75 | 132.2 | 126.57 | 43.68 | 118 | 58.67 | 1.27 | - |
| Angca_10764 | 64.64 | 51.54 | 77.06 | 90.1 | 77.05 | 62.4 | 56.57 | 13.81 | - |
| Angca_01453 | 26.25 | 26.48 | 26.89 | 29.25 | 27.76 | 32.88 | 53.81 | 84.91 | - |
| Angca_11655 | 39.16 | 33.41 | 28.97 | 39.48 | 45.41 | 32.52 | 50.68 | 132.65 | - |
| Angca_01501 | 48.66 | 58.96 | 54.3 | 61.62 | 55.96 | 53.6 | 50.62 | 39.33 | - |
| Angca_06568 | 29.6 | 19.69 | 21.3 | 20.92 | 16.67 | 39.92 | 50.56 | 64.68 | - |
| Angca_01014 | 42.1 | 36.5 | 34.45 | 40.72 | 42.23 | 32.86 | 50.46 | 134.27 | - |
| Angca_02648 | 37.43 | 30.96 | 23.78 | 40.33 | 44.18 | 26.04 | 49.38 | 81.62 | - |
| Angca_07359 | 9.27 | 16.02 | 26.06 | 30.46 | 22.52 | 7.66 | 46.4 | 31.62 | - |
| Angca_03959 | 46.66 | 43.97 | 44.54 | 41.21 | 37.59 | 36.58 | 46 | 65.81 | - |
| Angca_08020 | 51.34 | 68.37 | 144.01 | 58.8 | 93.79 | 49.53 | 44.26 | 0 | - |
| Angca_02308 | 14.78 | 13.31 | 22.82 | 19.86 | 29.25 | 29.75 | 43.98 | 55.97 | - |
| Angca_08839 | 18.92 | 16.19 | 21.4 | 24.79 | 34.18 | 30.8 | 43.21 | 55.34 | - |
| Angca_09036 | 73.19 | 88.51 | 114.99 | 56.86 | 37.79 | 62.12 | 41.51 | 9.25 | - |
| Angca_05006 | 20.78 | 21.6 | 22.93 | 22.92 | 32.11 | 32.83 | 40.92 | 54.29 | - |
| Angca_06662 | 8.45 | 6.04 | 5.06 | 14.02 | 12.78 | 15.79 | 40.25 | 66.92 | - |
| Angca_12541 | 49.53 | 61.68 | 61.67 | 40.3 | 32.9 | 53.69 | 38.16 | 8.71 | - |
| Angca_12306 | 27.95 | 25.23 | 31.83 | 32.74 | 25.61 | 29.65 | 37.27 | 34.23 | - |
| Angca_02915 | 32.94 | 37.64 | 34.97 | 27.37 | 34.94 | 33.93 | 37.05 | 8.9 | - |
| Angca_11126 | 14.65 | 18.29 | 16.54 | 20.93 | 17.34 | 22.9 | 34.32 | 44.76 | - |
| Angca_03611 | 1.12 | 1.06 | 1.32 | 1.1 | 2.06 | 8.94 | 32.75 | 178.83 | - |
| Angca_10148 | 21.55 | 14.4 | 15.8 | 19.23 | 22.2 | 21.62 | 31.03 | 54.39 | - |
| Angca_12047 | 18.82 | 16.45 | 20.95 | 22.5 | 22.82 | 28.94 | 30.7 | 32.88 | - |
| Angca_02464 | 11.38 | 12.03 | 20.83 | 16.74 | 23.23 | 18.88 | 30.51 | 31.39 | - |
| Angca_08383 | 18.16 | 20.74 | 20.93 | 21.38 | 22.24 | 22.27 | 29.48 | 42.07 | - |
| Angca_01043 | 198.45 | 250.13 | 188.12 | 211.46 | 131.3 | 48.11 | 29.22 | 3.07 | - |
| Angca_05159 | 24.59 | 30.83 | 31.6 | 29.32 | 27.86 | 44.59 | 29.07 | 50.49 | - |
| Angca_00767 | 16.42 | 17.88 | 19.27 | 18.57 | 21.26 | 18.96 | 28.24 | 69.32 | - |
| Angca_11891 | 11.97 | 9.47 | 9.64 | 7.9 | 15.63 | 16.6 | 28.21 | 69.89 | - |
| Angca_08515 | 21.46 | 32.82 | 47.07 | 32.97 | 27.72 | 25.01 | 26.83 | 11.34 | - |
| Angca_12745 | 16.12 | 14.76 | 19.12 | 20.22 | 18.34 | 20.33 | 26.7 | 24.57 | - |
| Angca_04301 | 14.23 | 5.02 | 7.1 | 11.43 | 10.29 | 26.53 | 26.68 | 86.99 | - |
| Angca_12296 | 20.65 | 27.23 | 8.64 | 23.68 | 38.58 | 18.52 | 26.06 | 81.83 | - |
| Angca_06403 | 14.83 | 14.08 | 17.67 | 11.95 | 11.75 | 17.68 | 25.85 | 51.78 | - |
| Angca_12995 | 14.89 | 10.46 | 8.14 | 14.03 | 27.99 | 16.71 | 25.37 | 112.95 | - |
| Angca_03595 | 24.37 | 25.88 | 25.84 | 21.39 | 22.39 | 21.98 | 25.29 | 14.94 | - |
| Angca_00116 | 10.21 | 9.32 | 10.87 | 12.75 | 16.72 | 16.16 | 25.08 | 39.58 | - |
| Angca_02431 | 19.46 | 20.15 | 15.1 | 19.64 | 16.89 | 12.83 | 23.14 | 61.49 | - |
| Angca_13054 | 13.46 | 12.04 | 13.79 | 14.88 | 12.66 | 16.05 | 22.94 | 40.27 | - |
| Angca_04551 | 55.73 | 35.98 | 43.17 | 47.49 | 36.37 | 29.26 | 22.94 | 1.77 | - |
| Angca_11243 | 0 | 0.68 | 5.16 | 3.94 | 0.45 | 6.61 | 22.91 | 45.3 | - |
| Angca_06047 | 15.22 | 14.46 | 18.27 | 22.51 | 14.5 | 21.04 | 22.68 | 31.04 | - |
| Angca_05043 | 13.87 | 18.3 | 20.04 | 14.1 | 15.07 | 10.57 | 22.65 | 51.81 | - |
| Angca_04573 | 19.35 | 23.19 | 9.02 | 39.75 | 9.57 | 15.02 | 21.38 | 0.51 | - |
| Angca_05877 | 11.54 | 7.3 | 11.38 | 12.27 | 9.03 | 15.49 | 20.88 | 27.98 | - |
| Angca_11069 | 12.36 | 12.8 | 15.14 | 14.42 | 17.62 | 16.47 | 20.22 | 32.44 | - |
| Angca_03528 | 11.78 | 10.96 | 12.77 | 12.96 | 13.98 | 13.2 | 20.19 | 27.37 | - |
| Angca_05757 | 24.66 | 26.89 | 33.07 | 27.52 | 17.84 | 30.88 | 19.61 | 12.8 | - |
| Angca_12418 | 13.4 | 11.35 | 12.22 | 12.71 | 16.45 | 13.44 | 19.41 | 27.43 | - |
| Angca_00984 | 20.49 | 21.56 | 23.01 | 14.4 | 14.9 | 26.44 | 19.17 | 3.04 | - |
| Angca_01226 | 17.83 | 19.58 | 21.01 | 15.8 | 24.22 | 21.37 | 18.98 | 10.71 | - |
| Angca_11374 | 21.28 | 19.9 | 7.75 | 8.32 | 9.69 | 3.55 | 17.75 | 145.36 | - |
| Angca_05144 | 19.79 | 21.64 | 10.1 | 18.21 | 17.57 | 24.05 | 17.51 | 3.97 | - |
| Angca_12260 | 28.7 | 23.34 | 23.86 | 27.23 | 24.77 | 17.53 | 17.34 | 4.22 | - |
| Angca_02224 | 28.59 | 11.36 | 9.99 | 24.37 | 15.6 | 26.55 | 17 | 7.95 | - |
| Angca_08538 | 19.72 | 5.73 | 7.89 | 0 | 32.02 | 0 | 16.86 | 75.12 | - |
| Angca_07259 | 12.28 | 16.81 | 8.22 | 10.44 | 13.57 | 7.43 | 15.9 | 2.12 | - |
| Angca_03423 | 10.12 | 8.04 | 9.79 | 10.2 | 15.23 | 12.07 | 15.67 | 21.29 | - |
| Angca_04656 | 19.05 | 23.24 | 21.58 | 24.88 | 21.22 | 17.23 | 14.84 | 9.1 | - |
| Angca_06674 | 9.54 | 7.34 | 10.08 | 12.08 | 12.41 | 12.45 | 14.34 | 26.34 | - |
| Angca_06220 | 7.42 | 6.19 | 6.72 | 8.02 | 5.72 | 8.37 | 14.29 | 45.49 | - |
| Angca_01606 | 8.25 | 7.37 | 7.52 | 8.84 | 13.07 | 8.81 | 14.24 | 34.51 | - |
| Angca_12079 | 10.24 | 11.02 | 11.32 | 10.2 | 13.41 | 9.62 | 13.01 | 29.94 | - |
| Angca_05658 | 18.48 | 16.47 | 17.57 | 16.84 | 22.82 | 18.74 | 12.89 | 4.03 | - |
| Angca_00466 | 6.97 | 7.17 | 4.33 | 6.42 | 6.95 | 7.22 | 12.63 | 32.76 | - |
| Angca_06940 | 15.16 | 10.92 | 20.56 | 18.81 | 15.39 | 12.91 | 12.35 | 26.99 | - |
| Angca_00728 | 6.19 | 4.59 | 5.94 | 7.33 | 11.63 | 9.38 | 12.29 | 18.51 | - |
| Angca_10081 | 24.91 | 21.76 | 29.66 | 27.14 | 17.04 | 15.99 | 12.14 | 2.14 | - |
| Angca_10400 | 9.43 | 14.78 | 17.48 | 17.73 | 12.2 | 15.38 | 11.94 | 3.99 | - |
| Angca_11689 | 2.51 | 5.15 | 5.03 | 7.66 | 2.73 | 6.1 | 11.68 | 28.93 | - |
| Angca_09545 | 12.28 | 10.38 | 11.63 | 10.97 | 12.41 | 8.5 | 11.43 | 7.1 | - |
| Angca_10775 | 25.37 | 27.56 | 20.91 | 21.85 | 23.04 | 16.94 | 11.37 | 6.12 | - |
| Angca_11217 | 7.64 | 19.12 | 23.15 | 15.68 | 17.43 | 11.29 | 11.33 | 2.01 | - |
| Angca_09265 | 10.82 | 14.54 | 15.08 | 12.22 | 13.28 | 9.39 | 11.29 | 11.17 | - |
| Angca_02574 | 3.74 | 2.28 | 4.3 | 5.62 | 9.06 | 6.15 | 11.04 | 18.62 | - |
| Angca_08517 | 9.09 | 7.49 | 9.67 | 4.95 | 16.22 | 13.55 | 10.94 | 32.15 | - |
| Angca_09359 | 7.38 | 12.46 | 0 | 23.17 | 6.68 | 8.74 | 10.87 | 0 | - |
| Angca_10864 | 5.85 | 1.96 | 5.59 | 5.83 | 13.4 | 8.68 | 10.38 | 46.41 | - |
| Angca_02118 | 10.03 | 9.36 | 8.48 | 8.16 | 9.61 | 9.47 | 10.27 | 11.07 | - |
| Angca_02618 | 10.98 | 10.37 | 13.16 | 10.05 | 10.5 | 11.78 | 10.05 | 4.21 | - |
| Angca_01331 | 6.25 | 4.01 | 4.57 | 6.53 | 6.6 | 4.57 | 10 | 40.13 | - |
| Angca_00237 | 0.21 | 0.33 | 2.99 | 1.51 | 1.88 | 4.83 | 9.84 | 18.48 | - |
| Angca_09443 | 16.12 | 17.6 | 16.72 | 17.13 | 8.17 | 12.23 | 9.67 | 3.19 | - |
| Angca_00755 | 2.3 | 2.34 | 2.59 | 2.44 | 2.16 | 4.16 | 9.4 | 26.76 | - |
| Angca_11644 | 26.31 | 58.32 | 49.08 | 18.14 | 34.7 | 27.4 | 8.87 | 17.44 | - |
| Angca_01928 | 1.17 | 1.34 | 4.57 | 5.22 | 9.03 | 5.58 | 8.71 | 29.05 | - |
| Angca_08377 | 4.7 | 3.8 | 5.08 | 6.2 | 8.09 | 5.35 | 8.16 | 14.09 | - |
| Angca_11452 | 6.16 | 5.75 | 6.45 | 5.87 | 5.65 | 6.24 | 8.1 | 9.2 | - |
| Angca_06181 | 50.36 | 51.09 | 65.16 | 46.28 | 27.98 | 23.41 | 8.09 | 0.72 | - |
| Angca_01636 | 1.82 | 2.95 | 5.08 | 3.47 | 2.42 | 3.71 | 7.96 | 15.17 | - |
| Angca_07112 | 12.44 | 17.39 | 14.47 | 12.02 | 8.53 | 10.5 | 7.94 | 4.4 | - |
| Angca_12168 | 13.88 | 20.16 | 16.2 | 21.96 | 18.55 | 10.44 | 7.8 | 0.73 | - |
| Angca_07418 | 0.01 | 0.01 | 0.18 | 0.11 | 0.19 | 2.93 | 7.77 | 56.83 | - |
| Angca_08623 | 7.82 | 7.08 | 9.09 | 8.15 | 7.19 | 3.97 | 7.66 | 1.54 | - |
| Angca_05635 | 12.61 | 18.48 | 5.2 | 22.52 | 15.12 | 25.47 | 7.53 | 0 | - |
| Angca_03892 | 0.37 | 0.28 | 0.27 | 0.33 | 0.47 | 1.24 | 6.72 | 33.57 | - |
| Angca_11134 | 6.12 | 6.14 | 5.52 | 4.24 | 6.89 | 5.58 | 6.65 | 7.69 | - |
| Angca_02911 | 8.45 | 7.45 | 6.86 | 8.28 | 8.41 | 8.31 | 6.65 | 1.64 | - |
| Angca_13093 | 6.43 | 6.05 | 5.74 | 6.26 | 5.38 | 5.51 | 6.55 | 8.78 | - |
| Angca_12114 | 7.08 | 7.74 | 6.53 | 6.19 | 8.25 | 6.11 | 6.43 | 3.89 | - |
| Angca_04484 | 6.74 | 6.76 | 7.46 | 6.67 | 6.66 | 7.8 | 6.27 | 4.03 | - |
| Angca_00248 | 5.28 | 5.36 | 4.17 | 5.39 | 5.14 | 5.12 | 6.26 | 12.62 | - |
| Angca_11277 | 2.94 | 3.09 | 3.06 | 3.73 | 4.57 | 4.54 | 6.14 | 6.77 | - |
| Angca_11291 | 5.66 | 7.37 | 5.63 | 4.76 | 6.56 | 5.95 | 6.13 | 2.98 | - |
| Angca_02169 | 2.23 | 0.98 | 1.8 | 3.16 | 5.65 | 3.65 | 5.89 | 33.43 | - |
| Angca_04781 | 4.56 | 7.66 | 7.6 | 4.37 | 3.79 | 5.88 | 5.84 | 3.04 | - |
| Angca_11753 | 3.16 | 3.06 | 2.56 | 3.08 | 5.98 | 4.35 | 5.8 | 8.67 | - |
| Angca_02780 | 0.39 | 0.22 | 1.68 | 1.12 | 2.44 | 3.28 | 5.75 | 8.59 | - |
| Angca_04628 | 3.68 | 3.58 | 4.01 | 4.3 | 4.6 | 4.96 | 5.54 | 6.33 | - |
| Angca_00274 | 4.49 | 5.04 | 4.28 | 4.1 | 5.25 | 4.25 | 5.4 | 5.21 | - |
| Angca_09058 | 10.88 | 10.17 | 8.38 | 9.42 | 7.71 | 7.67 | 5.33 | 1.34 | - |
| Angca_04611 | 6.65 | 4.2 | 4.35 | 4.08 | 4.21 | 3.79 | 5.3 | 0.87 | - |
| Angca_11491 | 2.97 | 3.25 | 4.87 | 5.2 | 3.34 | 4.42 | 5.12 | 1.36 | - |
| Angca_12544 | 2.26 | 1.28 | 2.3 | 4.43 | 5.12 | 2.2 | 5.11 | 19.07 | - |
| Angca_11017 | 6.51 | 6.82 | 7.37 | 8.21 | 3.64 | 6.17 | 4.98 | 1.35 | - |
| Angca_07181 | 3.19 | 2.03 | 2.6 | 3.27 | 2.08 | 4.65 | 4.85 | 7.37 | - |
| Angca_10909 | 3.18 | 3.71 | 5.72 | 6.45 | 3.46 | 3.06 | 4.79 | 2.41 | - |
| Angca_06876 | 8.93 | 16.66 | 6.62 | 23.25 | 7.09 | 5.83 | 4.74 | 1.96 | - |
| Angca_08408 | 0.98 | 1.23 | 2.14 | 1.71 | 3.68 | 2.33 | 4.55 | 5.83 | - |
| Angca_09056 | 1.52 | 0.68 | 2.15 | 2.1 | 3.42 | 3.34 | 4.4 | 7.44 | - |
| Angca_03193 | 1.93 | 1.72 | 2.51 | 2.51 | 2.79 | 3.18 | 3.78 | 5.18 | - |
| Angca_12310 | 2.74 | 2.52 | 3.01 | 3.13 | 4.43 | 3.28 | 3.76 | 4.14 | - |
| Angca_02426 | 6.1 | 5.92 | 7.35 | 6.78 | 5.88 | 8.49 | 3.76 | 1.72 | - |
| Angca_02556 | 5 | 5.57 | 6.33 | 3.95 | 2.43 | 2.49 | 3.52 | 0.83 | - |
| Angca_00501 | 3.98 | 4.13 | 3.8 | 3.74 | 3.56 | 3.13 | 3.46 | 1.66 | - |
| Angca_07882 | 2.86 | 3.47 | 2.94 | 1.9 | 1.98 | 2.04 | 3.25 | 0.38 | - |
| Angca_08643 | 1.03 | 0.76 | 1.23 | 1.19 | 1.68 | 1.86 | 3.23 | 5.85 | - |
| Angca_12452 | 2.84 | 5.16 | 4.91 | 3.32 | 3.04 | 2.47 | 3.2 | 0 | - |
| Angca_07386 | 4.53 | 3.95 | 3.98 | 3.58 | 4.8 | 4.02 | 3.11 | 3.15 | - |
| Angca_07988 | 0.99 | 0.83 | 1.61 | 1.6 | 3.93 | 3.22 | 3.03 | 5.83 | - |
| Angca_05807 | 2.61 | 2.54 | 2.38 | 2.24 | 2.39 | 2.08 | 3.02 | 3.29 | - |
| Angca_00915 | 1.88 | 2.12 | 3.78 | 2.01 | 0.48 | 1.45 | 2.96 | 0.42 | - |
| Angca_10406 | 5.08 | 8.7 | 12.78 | 4.24 | 3.62 | 4.32 | 2.95 | 0.09 | - |
| Angca_00127 | 1.04 | 0.78 | 1.28 | 3.1 | 4.23 | 2.65 | 2.95 | 8.63 | - |
| Angca_07515 | 2.01 | 1.75 | 1.99 | 2.51 | 2.66 | 2.1 | 2.83 | 3.86 | - |
| Angca_12700 | 2.85 | 3.2 | 5.45 | 2.82 | 3.15 | 4.27 | 2.63 | 0.84 | - |
| Angca_03391 | 0.28 | 0.29 | 0.96 | 0.44 | 0.5 | 1.31 | 2.54 | 3.12 | - |
| Angca_11235 | 0.59 | 0.37 | 0.56 | 0.61 | 0.62 | 1.2 | 2.53 | 5.41 | - |
| Angca_03059 | 1.2 | 1.4 | 0.69 | 0.8 | 0.48 | 1.37 | 2.53 | 3.76 | - |
| Angca_12824 | 3.27 | 2.62 | 3.19 | 3.75 | 2.22 | 3.11 | 2.35 | 0.1 | - |
| Angca_05813 | 1.57 | 1.24 | 1.71 | 1.63 | 1.81 | 1.67 | 2.31 | 3.4 | - |
| Angca_00151 | 3.88 | 4.66 | 4.57 | 3.63 | 3.9 | 3.53 | 2.24 | 0.69 | - |
| Angca_03412 | 5.08 | 5.34 | 5.24 | 3.48 | 2.16 | 2.33 | 2.23 | 0.23 | - |
| Angca_02749 | 1.37 | 1.03 | 1.24 | 1.24 | 1.71 | 1.71 | 2.21 | 2.65 | - |
| Angca_10516 | 2.94 | 1.92 | 1.65 | 2.81 | 2.19 | 2.85 | 2.15 | 0.91 | - |
| Angca_04939 | 0.07 | 0.08 | 0.11 | 0.1 | 0.11 | 0.4 | 2.06 | 11.24 | - |
| Angca_00168 | 0.46 | 0.13 | 0.59 | 0.18 | 2.09 | 0.83 | 2.05 | 6.46 | - |
| Angca_13301 | 1.11 | 0.82 | 1.12 | 1.01 | 1.86 | 1.22 | 2.02 | 2.65 | - |
| Angca_13386 | 0.15 | 0.11 | 0.29 | 0.27 | 0.29 | 0.81 | 1.98 | 7.17 | - |
| Angca_11860 | 1.07 | 0.98 | 1.72 | 1.72 | 0.93 | 1.41 | 1.95 | 0.21 | - |
| Angca_07831 | 1.54 | 1.44 | 1.48 | 1.62 | 1.78 | 1.44 | 1.95 | 2.73 | - |
| Angca_02443 | 1.31 | 2.31 | 1.65 | 1.18 | 2.09 | 0.88 | 1.86 | 0.12 | - |
| Angca_07568 | 1.59 | 1.62 | 1.27 | 2.18 | 1.32 | 1.53 | 1.85 | 0.29 | - |
| Angca_00888 | 1.01 | 0.48 | 1.1 | 1.75 | 1.73 | 1.65 | 1.85 | 3.85 | - |
| Angca_13341 | 2.14 | 1.47 | 1.65 | 1.61 | 2.01 | 1.06 | 1.8 | 0.51 | - |
| Angca_10983 | 3.75 | 2.32 | 2.15 | 2.55 | 2.65 | 2.39 | 1.78 | 0.35 | - |
| Angca_08468 | 2.46 | 1.88 | 2.27 | 2.21 | 2.19 | 1.85 | 1.74 | 0.95 | - |
| Angca_07953 | 0 | 0.05 | 0.42 | 0.23 | 0.78 | 1.28 | 1.66 | 9.14 | - |
| Angca_09871 | 1.82 | 1.28 | 1.58 | 0.9 | 1.25 | 0.4 | 1.61 | 50.4 | - |
| Angca_07039 | 2.59 | 2.47 | 2.31 | 3.24 | 1.01 | 1.93 | 1.61 | 0.64 | - |
| Angca_00389 | 1.72 | 1.68 | 1.62 | 1.7 | 1.39 | 1.72 | 1.59 | 2.68 | - |
| Angca_01450 | 1.72 | 1.27 | 0.93 | 0.94 | 1.2 | 1.19 | 1.49 | 0.21 | - |
| Angca_04782 | 1.32 | 1.83 | 1.99 | 1.48 | 1.27 | 1.46 | 1.48 | 0.72 | - |
| Angca_11709 | 1.21 | 1.12 | 1.3 | 1.16 | 1 | 1.72 | 1.47 | 2.24 | - |
| Angca_05606 | 4.92 | 5.21 | 3.35 | 3.25 | 1.25 | 3 | 1.4 | 0.5 | - |
| Angca_04789 | 5.34 | 5.92 | 8.85 | 5.06 | 4.09 | 1.81 | 1.39 | 0.31 | - |
| Angca_03447 | 0.43 | 0.5 | 0.63 | 0.71 | 1.17 | 0.85 | 1.32 | 2.48 | - |
| Angca_01089 | 3.1 | 3 | 2.71 | 3.06 | 3.96 | 2.14 | 1.27 | 0.44 | - |
| Angca_09044 | 1.08 | 1.55 | 1.88 | 1.61 | 1.04 | 1.05 | 1.23 | 0.77 | - |
| Angca_11070 | 0.8 | 0.66 | 0.55 | 1.18 | 1.14 | 1.25 | 1.22 | 1.53 | - |
| Angca_04629 | 0.96 | 0.6 | 1.01 | 1.26 | 1.15 | 0.73 | 1.16 | 2.34 | - |
| Angca_04814 | 1.57 | 1.85 | 1.63 | 1.7 | 1.55 | 1 | 1.05 | 0.93 | - |
| Angca_05458 | 1.28 | 1.07 | 1.43 | 2.31 | 1.27 | 1.38 | 0.93 | 0.31 | - |
| Angca_04815 | 1.51 | 1.52 | 1.34 | 0.97 | 1.3 | 1.03 | 0.68 | 0.46 | - |
| Angca_11604 | 1.69 | 0.52 | 1.19 | 1.65 | 1.64 | 1.64 | 0.61 | 0.13 | - |
| Angca_11339 | 0.48 | 0.74 | 4.27 | 1.64 | 2.44 | 0.61 | 0.57 | 0 | - |
| Angca_01098 | 2.02 | 0.73 | 1.06 | 1.41 | 2.11 | 1.9 | 0.3 | 0.72 | - |
| Angca_12431 | 0 | 0 | 0 | 0 | 0 | 0.2 | 0.2 | 69.86 | - |
| Angca_04567 | 1.63 | 1.01 | 1.68 | 1.81 | 1.02 | 0.81 | 0 | 0.3 | - |
| Angca_02242 | 0 | 0 | 0 | 0 | 0 | 0 | 0 | 59.71 | - |
| Angca_03002 | 0.3 | 1.61 | 1.54 | 1.98 | 2.51 | 2.21 | 0 | 0 | - |
| Angca_02651 | 0 | 0 | 2.44 | 0 | 2.59 | 0 | 0 | 28.98 | - |
| Angca_04945 | 0 | 9.86 | 7.91 | 18.4 | 7.37 | 0 | 0 | 0 | - |
| Angca_03848 | 0 | 0 | 0 | 0 | 78.43 | 0 | 0 | 176.21 | - |
| Angca_03747 | 0 | 0 | 5.26 | 0 | 0 | 0 | 0 | 6.3 | - |
| Angca_12411 | 0 | 0 | 0 | 0 | 0 | 0 | 0 | 72.3 | - |
| Angca_05173 | 0 | 0 | 0 | 0 | 609.67 | 0 | 0 | 1253.96 | - |

Note: M14B, Larvae from mouse brains at 14 dpi; R14B, Larvae from rat brains at 14 dpi; M21B, Larvae from mouse brains at 21 dpi; R21B, Larvae from rat brains at 21 dpi; M28B, Larvae from mouse brains at 28 dpi; R28B, Larvae from rat brains at 28 dpi; R28L, Larvae from rat lungs at 28 dpi; R55L, Larvae from rat lungs at 55 dpi.

**Table E siRNA sequences for RNAi of *Acnr1f* gene.**

| siRNA | siRNA sequence |
| --- | --- |
| *Acnr1f*- 94 | F：5'- GCGAGGGAUGUAAAGGUUUTT -3' |
|  | R：5'-AAACCUUUACAUCCCUCGCTT -3' |
| *Acnr1f-*894 | F：5'- GCAAAUCCACUUACUCAAATT -3' |
|  | R：5'- UUUGAGUAAGUGGAUUUGCTT -3' |
| *Acnr1f*-1219 | F：5'-GCUCAGAGAGUUUGGGAUATT -3' |
|  | R：5'-UAUCCCAAACUCUCUGAGCTT-3' |

**Table F RNAi effects of three siRNAs on adult worms by qPCR analysis (n=3).**

| Group | Blank | Negative | *Acnr1f*-94 | *Acnr1f*-894 | *Acnr1f*-1219 |
| --- | --- | --- | --- | --- | --- |
| Gene expression levels (relative to *Acgapdh*) | 1.00 ± 0.00 | 1.25 ± 0.32 | 1.51 ± 0.42 | 0.57 ± 0.21 | 0.62 ± 0.09 |

**Table G *Acnr1f* gene transcription in F0 generation of adult worms treated with lentivirus LV-*Acnr1f*-894 and LV-*Acnr1f*-1219 at MOI of 400 by qPCR analysis (n=3).**

| Group | WT | NT | *Acnr1f*-894 | *Acnr1f*-1219 |
| --- | --- | --- | --- | --- |
| Gene expression levels (relative to *Acgapdh*) | 0.96 ± 0.06 | 0.99 ± 0.05 | 0.62 ± 0.22 | 0.88 ± 0.05 |

**Table H Number of L4 larvae (21 dpi) from rat brains among WT, EV and RNAi groups (n=4).**

| Group | WT | NT | *Acnr1f*-894 |
| --- | --- | --- | --- |
| L4 larvae | 29.00 ± 2.94 | 37.25 ± 2.75 | 10.00 ± 10.89 |

**Table I Percentage of L5 larvae delaying in rat brains** **treatment with LV-*Acnr1f*-894 (n=4).**

| Group | Number of L4 larvae in rat brains at 21 dpi | Number of L5 larvae in rat brains at 28 dpi | L5/L4 (%) |
| --- | --- | --- | --- |
| WT | 116 | 36 | 31.03 |
| EV | 149 | 16 | 10.74 |
| *Acnr1f*-894 | 40 | 26 | 65.00 |

**Table J Number of L1 larvae from per gram of rat feces among WT, EV and RNAi groups (n=3).**

| Dpi | WT | EV | Ac-nr1f-894 |
| --- | --- | --- | --- |
| 54 | 22909 | 17087 | 10556 |
| 58 | 18231 | 18743 | 13161 |
| 62 | 27433 | 25039 | 20690 |
| 66 | 26313 | 26000 | 23988 |
| 70 | 21857 | 19273 | 17524 |
| 74 | 27961 | 27591 | 23207 |
| 78 | 18824 | 18182 | 16189 |

**Table K Number of F1 generation of eggs laid from per female among WT, EV and RNAi groups (n=3).**

| Group | WT | NT | *Acnr1f*-894 |
| --- | --- | --- | --- |
| Eggs | 446.76 ± 70.63 | 417.41 ± 72.41 | 150.42 ± 82.18 |

**Table L Length of the eggs (μm) among WT, EV and RNAi groups (n=15).**

| Group | WT | NT | *Acnr1f*-894 |
| --- | --- | --- | --- |
| Egg length | 89.87 ± 8.59 | 86.82 ± 8.11 | 79.06 ± 5.60 |

**Table M Hatching rate** **of F1 generation of eggs to L1 larvae from F0 generation of adult worms among WT, EV and RNAi groups (n=3).**

| Group | WT | NT | *Acnr1f*-894 |
| --- | --- | --- | --- |
| Hatching rate of eggs | 0.41 ± 0.08 | 0.33 ± 0.06 | 0.11 ± 0.11 |

**Table N Masson staining of F0 generation of adult worms for observing cuticle thickness among WT, EV and RNAi groups (μm) (n=15).**

| Group | WT | NT | *Acnr1f*-894 |
| --- | --- | --- | --- |
| Cuticle thickness | 21.40 ± 1.31 | 20.24 ± 1.34 | 12.65 ± 2.06 |

**Table O GO enrichment of DEGs of F0 generation of adult worms between EV and RNAi groups.**

| **Term** | **ID** | **Input number** | **Background number** | **P-value** |
| --- | --- | --- | --- | --- |
| Cuticle development involved in collagen and cuticulin-based cuticle molting cycle | GO:0042338 | 7 | 27 | 4.97259E-08 |
| Cell differentiation | GO:0030154 | 16 | 303 | 2.70554E-07 |
| Post-embryonic body morphogenesis | GO:0040032 | 5 | 16 | 2.01447E-06 |
| Cell adhesion | GO:0007155 | 6 | 38 | 5.85724E-06 |
| Intracellular signal transduction | GO:0035556 | 11 | 184 | 6.5701E-06 |
| Extracellular region | GO:0005576 | 34 | 325 | 1.95611E-22 |
| Plasma membrane | GO:0005886 | 43 | 875 | 2.32373E-16 |
| Collagen trimer | GO:0005581 | 9 | 55 | 2.01469E-08 |
| Cell junction | GO:0030054 | 13 | 163 | 4.31143E-08 |
| Integral component of plasma membrane | GO:0005887 | 23 | 567 | 7.29032E-08 |
| Cytosolic small ribosomal subunit | GO:0022627 | 7 | 32 | 1.35994E-07 |
| Metal ion binding | GO:0046872 | 52 | 1381 | 5.35558E-15 |
| Hydrolase activity | GO:0016787 | 38 | 831 | 1.16361E-13 |
| Structural constituent of cuticle | GO:0042302 | 18 | 166 | 9.91317E-13 |
| Structural constituent of ribosome | GO:0003735 | 16 | 143 | 1.18187E-11 |
| Metalloendopeptidase activity | GO:0004222 | 13 | 106 | 3.60924E-10 |
| Extracellular matrix structural constituent | GO:0005201 | 7 | 34 | 1.95279E-07 |
| DNA binding | GO:0003677 | 27 | 834 | 4.26444E-07 |
| Structural constituent of collagen and cuticulin-based cuticle | GO:0042329 | 5 | 13 | 8.70107E-07 |
| ATP binding | GO:0005524 | 25 | 909 | 1.74369E-05 |

**Table P Expression pattern of 36 collagens in the F0 generation of adult worms with Log2(FPKM)>1 among WT, EV and RNAi groups.**

| Gene ID | WT | EV | *Acnr1f*-894 |
| --- | --- | --- | --- |
| Angca_01175 | 9.55 | 9.32 | 8.44 |
| Angca_03330 | 9.24 | 9.02 | 8.11 |
| Angca_03162 | 9.06 | 9.00 | 3.61 |
| Angca_02006 | 9.02 | 8.41 | 9.18 |
| Angca_12810 | 8.78 | 8.91 | 7.06 |
| Angca_08172 | 8.78 | 8.62 | 5.58 |
| Angca_01778 | 8.77 | 8.39 | 7.23 |
| Angca_10659 | 8.45 | 8.33 | 2.96 |
| Angca_12807 | 8.35 | 8.10 | 3.20 |
| Angca_11567 | 8.29 | 7.97 | 8.30 |
| Angca_11566 | 5.39 | 5.20 | 4.14 |
| Angca_12041 | 5.15 | 5.92 | 4.97 |
| Angca_09844 | 5.09 | 5.14 | 4.53 |
| Angca_03226 | 4.80 | 4.55 | 3.34 |
| Angca_07247 | 4.74 | 4.44 | 3.30 |
| Angca_07290 | 4.74 | 4.50 | 4.57 |
| Angca_09292 | 4.50 | 4.47 | 3.24 |
| Angca_07682 | 3.81 | 3.76 | 4.19 |
| Angca_10552 | 3.64 | 4.10 | 2.67 |
| Angca_00201 | 3.54 | 3.60 | 3.69 |
| Angca_08523 | 3.17 | 3.14 | 3.22 |
| Angca_08001 | 2.84 | 2.16 | 3.14 |
| Angca_11042 | 2.67 | 3.11 | 2.83 |
| Angca_05058 | 2.49 | 2.36 | 2.93 |
| Angca_11776 | 2.21 | 1.73 | 1.99 |
| Angca_13233 | 2.14 | 1.78 | 2.45 |
| Angca_02605 | 2.03 | 2.59 | 1.68 |
| Angca_00905 | 1.98 | 2.03 | 2.06 |
| Angca_08234 | 1.84 | 2.46 | 2.16 |
| Angca_02486 | 1.82 | 1.18 | 1.73 |
| Angca_03334 | 1.82 | 1.66 | 1.46 |
| Angca_04789 | 1.43 | 1.18 | 1.16 |
| Angca_03328 | 1.36 | 1.07 | 0.68 |
| Angca_01814 | 1.34 | 1.10 | 1.53 |
| Angca_11607 | 1.33 | 0.95 | 0.79 |
| Angca_12370 | 1.24 | 1.15 | 0.70 |

**Table Q Identification of two *collagen* genes as downstream targets of *Ac*NR1F by dual-luciferase reporter assays (n=3).**

| Group | Blank ( pGL3-Basic) | Angca_01175 | Angca_01778 |
| --- | --- | --- | --- |
| RLU (fold induction) | 1.00 ± 0.69 | 311.51 ± 62.41 | 43.53 ± 16.82 |

**Table R Expression pattern of two *collagen* genes across developmental stages of *A. cantonensis* (Log2FPKM).**

| Gene ID | L1 | L3 | L4 | L5 | AF | AM |
| --- | --- | --- | --- | --- | --- | --- |
| Angca_01778 | 6.24 | 4.10 | 11.55 | 12.94 | 10.72 | 9.59 |
| Angca_01175 | 7.97 | 4.07 | 9.99 | 11.66 | 10.38 | 9.75 |

Note: L1, First stage larvae; L3, Third stage larvae; L4, Fourth stage larvae; L5, Fifth stage larvae; AF, Adult female; AM, Adult male.

**Table S LC-MS/MS analysis of steroids contents in the brain of SD rats, BALB/c mice, and larvae at 28 dpi (n=6, ng/g dry weight).**

| Steroids | SD rat brain | Balb/c mouse brain | Infected SD rat brain | Infected Balb/c mouse brain | Larvae from SD rat | Larvae from Balb/c mouse |
| --- | --- | --- | --- | --- | --- | --- |
| Desmosterol | 12295.3±771.1 | 15647.0±1066.8 | 16476.3±1128.9 | 12586.6±2079.9 | -- | -- |
| Lathosterol | 760.8±100.7 | 623.6±67.4 | 689.4±140.3 | 505.5±52.0 | 361.2±98.6 | 3785.7±525.8 |
| Cholesterol | 764.0±96.8 | 583.8±120.9 | 682.0±144.8 | 531.1±22.5 | 346.0±84.9 | 3604.3±415.7 |
| 25-Hydroxycholesterol | -- | -- | -- | -- | 506.7±117.6 | 701.7±43.9 |
| 7α,25-Dihydroxycholesterol | 788.8±184.4 | 159.1±34.1 | 594.3±83.8 | -- | 4457.6±2009.8 | 972.7±47.9 |
| Corticosterone | 21.3±9.4 | 22.0±12.5 | 121.1±43.5 | 305.1±160.0 | 331.6±92.6 | 1158.0±16.7 |
| Deoxycorticosterone | 0.3±0.1 | 0.6±0.6 | 1.9±0.5 | 3.9±1.7 | 3.7±1.0 | 16.8±0.3 |
| 11-Dehydrocorticosterone | 2.0±1.3 | 1.3±0.6 | 13.1±11.2 | 18.8±12.2 | 175.7±56.3 | 87.9±2.5 |
| 7-Ketocholesterol | 21.2±5.8 | 54.0±26.9 | 22.0±4.7 | 45.7±18.8 | 18.4±12.4 | 46.1±3.4 |
| β-Sitosterol | -- | -- | -- | -- | 17.8±7.5 | 73.1±15.3 |
| 24-Hydroxycholesterol | 2802.6±1866.7 | 4110.9±2560.5 | 1486.6±405.6 | 5792.0±0.0 | 226.1±75.6 | -- |
| Pregnenolone | 8.0±2.2 | -- | 14.2±4.7 | 8.1±0.0 | -- | -- |
| 7a,27-Dihydroxycholesterol | 0.6±0.1 | 0.6±0.1 | 0.7±0.2 | -- | 6.6±1.7 | 4.0±0.3 |
| Cortisol | -- | -- | 7.1±0.0 | -- | -- | -- |
| Progesterone | 4.1±4.6 | 0.3±0.1 | 2.2±0.9 | 0.3±0.2 | 0.1±0.0 | 0.0±0.0 |
| 17β-Estradiol | 1.6±0.1 | 1.6±0.3 | 2.9±1.5 | 1.6±0.4 | 2.1±1.1 | 4.3±0.5 |
| 16α-Hydroxyestrone | 0.6±0.2 | 0.6±0.1 | 0.6±0.1 | 1.2±0.3 | -- | -- |
| 5α-Pregnane-3,20-dione | 3.0±2.7 | -- | 2.3±0.9 | 2.3±0.0 | -- | -- |
| 5β-Androsterone | 10.4±3.8 | 6.6±2.2 | 8.2±1.4 | 9.4±1.7 | -- | -- |
| Testosterone | 0.0±0.0 | 0.0±0.0 | 0.0±0.0 | 0.1±0.0 | 0.1±0.0 | -- |
| Androstenedione | 0.1±0.0 | 0.0±0.0 | 0.1±0.0 | 0.1±0.1 | 0.1±0.0 | 0.1±0.0 |
| 11-Hydroxyandrosterone | -- | -- | 2.9±1.4 | -- | -- | -- |

**Table T Binding activities of cholesterol, corticosterone and 7α25-OHC to *Ac*NR1F assessed by dual-luciferase reporter assays.**

| Conc.  Steroids | Blank | 1 μM | 5 μM | 10 μM | 20 μM | 50 μM | 75 μM | 100 μM |
| --- | --- | --- | --- | --- | --- | --- | --- | --- |
| Cholesterol | 1.00 ± 0.09 | 1.03±0.17 | 1.10±0.23 | 1.08±0.16 | 1.19±0.11 | 1.60±0.38 | 2.53±0.50 | 2.84±0.46 |
| Corticosterone | 1.00 ±0.07 | 0.98 ±0.12 | 1.05 ±0.12 | 1.24 ±0.08 | 1.68 ±0.21 | 2.61 ±0.40 | 3.97 ± 0.49 | 5.48 ±1.56 |
| 7α25OHC | 1.00 ±0.02 | 0.93 ±0.07 | 0.95 ±0.05 | 0.91 ±0.04 | 1.14 ±0.25 | 1.44 ±0.11 | 1.88 ±0.18 | 2.35 ±0.34 |
