# Supplementary material for: AcNR1F orchestrates epidermal remodeling to enable host adaptation in Angiostrongylus cantonensis
Source: PLoS Pathog. 2026 Oct 12;22(10):e1014691. doi: 10.1371/journal.ppat.1014691 (PMC13657317; doi:10.1371/journal.ppat.1014691)
Supplement: Supplementary file 2 [file ppat.1014691.s002.docx]

**S1_Raw_Images**

**Figure 1. Identification and analysis of expression pattern of *Acnr1f.***

**Raw image A. This is the original gel electrophoresis image for Figure 1B.**

**Figure 3. Lentivirus-mediated RNAi of *Acnr1f* inhibits the development and reproduction of *A. cantonensis*.**

**Raw image B. The right image is the original gel electrophoresis image for Figure 3C.**

**Raw image C. This is the original gel electrophoresis image for Figure 3D.**

**Figure A. Gel electrophoresis of PCR fragment of *Acnr1f* gene**

M: DNA marker; 1. Amplification of *Acnr1f* CDS fragment PCR.

**Raw image D. This is the original gel electrophoresis image for Figure A.**

**Figure C Gel electrophoresis of PCR fragments of collagen gene promoter region**

M: DNA marker; (A) *Accol-12*-promotor; (B) *Accol-144*-promotor.

**Raw image E. This is the original gel electrophoresis image for Figure CA.**

**Raw image F. This is the original gel electrophoresis image for Figure CB.**

**Figure D Gel electrophoresis of CDS region of *Acnr1f* and pcDNA3.1 vector after *Bam*H I and *Eco*R I digestion**

M: DNA marker; 1. CDS region of *Acnr1f*; 2. pcDNA3.1 vector.

**Raw image G. This is the original gel electrophoresis image for Figure D.**

**Figure E Gel electrophoresis of PCR fragment of the ligand-binding domain of *Ac*NR1F**

M: DNA marker; 1. PCR amplification of the ligand-binding domain of *Ac*NR1F.

**Raw image H. This is the original gel electrophoresis image for Figure E.**
